# A new advanced *in silico* drug discovery method for novel coronavirus (SARS-CoV-2) with tensor decomposition-based unsupervised feature extraction

**DOI:** 10.1371/journal.pone.0238907

**Published:** 2020-09-11

**Authors:** Y-h. Taguchi, Turki Turki

**Affiliations:** 1 Department of Physics, Chuo University, Tokyo, Japan; 2 Department of Computer Science, King Abdulaziz University, Jeddah, Saudi Arabia; University of Alabama at Birmingham, UNITED STATES

## Abstract

Background: COVID-19 is a critical pandemic that has affected human communities worldwide, and there is an urgent need to develop effective drugs. Although there are a large number of candidate drug compounds that may be useful for treating COVID-19, the evaluation of these drugs is time-consuming and costly. Thus, screening to identify potentially effective drugs prior to experimental validation is necessary. Method: In this study, we applied the recently proposed method tensor decomposition (TD)-based unsupervised feature extraction (FE) to gene expression profiles of multiple lung cancer cell lines infected with severe acute respiratory syndrome coronavirus 2. We identified drug candidate compounds that significantly altered the expression of the 163 genes selected by TD-based unsupervised FE. Results: Numerous drugs were successfully screened, including many known antiviral drug compounds such as C646, chelerythrine chloride, canertinib, BX-795, sorafenib, sorafenib, QL-X-138, radicicol, A-443654, CGP-60474, alvocidib, mitoxantrone, QL-XII-47, geldanamycin, fluticasone, atorvastatin, quercetin, motexafin gadolinium, trovafloxacin, doxycycline, meloxicam, gentamicin, and dibromochloromethane. The screen also identified ivermectin, which was first identified as an anti-parasite drug and recently the drug was included in clinical trials for SARS-CoV-2. Conclusions: The drugs screened using our strategy may be effective candidates for treating patients with COVID-19.

## 1 Introduction

Coronavirus 2019 (COVID-19) is an infectious disease that has created a pandemic worldwide [1]. Thus, it is urgent to identify effective drugs to combat this disease. Numerous studies related to identifying effective therapeutics have been reported; *in slico* drug discovery is a useful approach because very large numbers (up to millions) of drug candidate compounds can be screened, which is not possible using experimental approaches. There are two main methods used for *in slico* drug discovery: ligand-based drug discovery (LBDD) and structure-based drug discovery (SBDD), which have various advantages and disadvantages. LBDD can effectively predict “hit” compounds, but cannot find new drug candidate compounds lacking similarity to known drug compounds. In contrast, although SBDD can find drug candidate compounds without similarity to known drugs, it requires massive computational resources for docking simulation between compounds and proteins. When no experimentally confirmed protein tertiary structures are available, these structures must also be predicted, potentially decreasing the accuracy of the predicted affinity of compounds with proteins. As in [2–5], if gene expression profiles altered by new drug candidate compounds are coincident with those of known drug compounds, these new drug candidate compounds are regarded as promising. Although this approach can identify promising drug candidate compounds even when they lack similarity with known drugs, as required by LBDD, and massive computational resources are not needed, as required by SBDD, it remains difficult to identify drug candidate compounds for proteins and diseases when no effective drug compounds are known.

To overcome these limitations, we propose an unsupervised method that can predict drug candidate compounds without knowledge of known compounds using a different formulation of the recently proposed tensor decomposition (TD)-based unsupervised feature extraction (FE) [5]. TD-based unsupervised FE was applied to the gene expression profiles of multiple lung cancer cell lines infected with severe acute respiratory syndrome coronavirus 2 (SARS-CoV-2) [6]. The 163 genes identified as differentially expressed genes (DEGs) in SARS-CoV-2 infection were enriched in various SARS coronavirus-related terms. Drugs screened based on the coincidence of DEGs between drug treatments and SARS-CoV-2 infection were largely enriched with known antivirus drugs. This suggests that our strategy is effective and that the drugs screened in this study are promising candidates as antiviral drug for SARS-CoV-2.

## 2 Materials and methods

**Fig 1:**
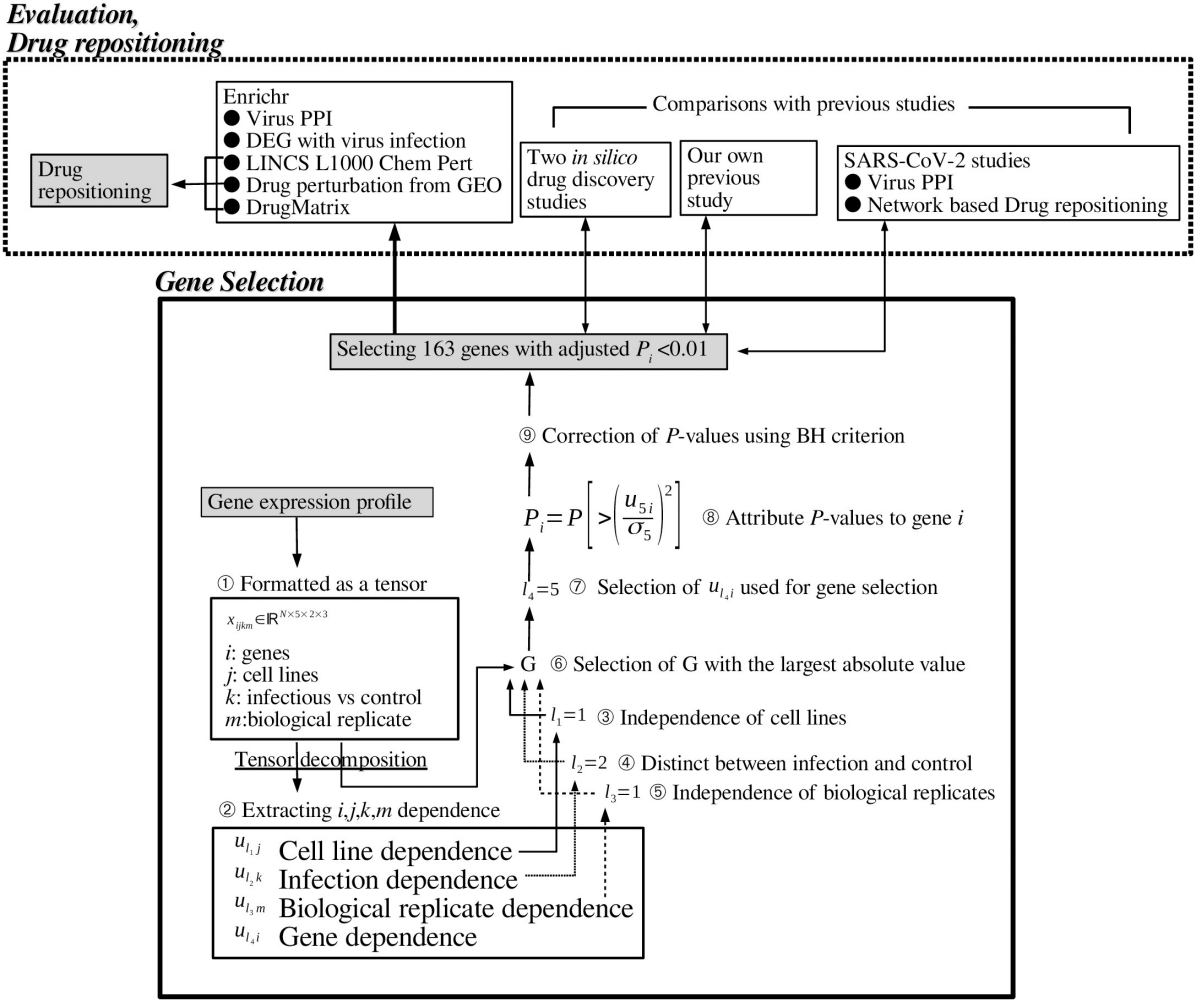
Overall design of this study.

Fig 1 shows the overall design of this study.

### 2.1 Gene expression profiles

Gene expression profiles used in this study were downloaded from the Gene Expression Omnibus (GEO) with GEO ID GSE147507. Specifically, the file used was GSE147507_RawReadCounts_Human.tsv.gz; it is composed of five cell lines (Calu3, NHBE, A549 Multiplicity of infection (MOI) 0.2, A549 MOI 2.0, and A549 ACE2 expressed), two treatments (Mock and SARS-CoV-2 infected), and three biological replicates for individual pairs of cell lines and treatments. Thus, in total, 5 × 2 × 3 = 30 samples were available.

### 2.2 TD-based unsupervised FE

The purpose of applying TD to gene expression was to identify genes simultaneously associated with or dependent on multiple experimental conditions, i.e., infection, cell lines, and biological replicates.

Gene expression profiles are formatted as tensor, $x_{ijkm}\in R^{N\times 5\times 2\times 3}$, which represents the *i*th gene expression of *j*th cell lines (*j* = 1: Calu3, *j* = 2: NHBE, *j* = 3: A549 MOI 0.2, *j* = 4: A549 MOI 2,0, *j* = 5: A549 ACE2 expressed) with *k*th treatment (*k* = 1: Mock and *k* = 2: SARS-CoV-2 infected) of the *m*th biological replicates.

*x*_*ijkm*_ was decomposed into TD

$$\begin{array}{c} x_{ijkm}=\;\sum_{\ell_{1}=1}^{5}\sum_{\ell_{2}=1}^{2}\sum_{\ell_{3}=1}^{3}\sum_{\ell_{4}=1}^{N}G\left(\ell_{1},\ell_{2},\ell_{3},\ell_{4},\ell_{5}\right)u_{\ell_{1}j}u_{\ell_{2}k}u_{\ell_{3}m}u_{\ell_{4}i} \end{array}$$ (1)

with a higher-order singular value decomposition (HOSVD) [5]. $u_{\ell_{1}j}\in R^{5\times 5},u_{\ell_{2}k}\in R^{2\times 2},u_{\ell_{3}m}\in R^{3\times 3},u_{\ell_{4}i}\in R^{N\times N}$ are singular value matrices which are orthogonal matrices. The tensor was normalized as ∑_*i*_ *x*_*ijkm*_ = 0 and $\sum_{i}x_{ijkm}^{2}=N$. $G\left(\ell_{1},\ell_{2},\ell_{3},\ell_{4}\right)\in R^{5\times 2\times 3\times N}$ is a core tensor that represents a weight of the combination of *ℓ*_1_, *ℓ*_2_, *ℓ*_3_, *ℓ*_4_.

TD assumes that a tensor can be expressed as a summation of series of product of four singular value vectors, *u*_*ℓ*_1_*j*_, *u*_*ℓ*_2_*k*_, *u*_*ℓ*_3_*m*_, and *u*_*ℓ*_4_*i*_, each of which represents the dependence upon *j*, *k*, *m*, and *i*, with the weight *G*. Generally, we cannot expect that these dependencies represent something biological, as it is purely a mathematical assumption. Thus, we need to seek the singular value vectors that represent the biological dependence. Only occasionally do we find biological singular value vectors, and then we can go further.

To identify *u*_*ℓ*_4_*i*_ which is used for gene selection, we need to identify *u*_*ℓ*_1_*j*_ whose values are independent of *j*, i.e. cell line-independent, *u*_*ℓ*_2_*m*_ whose values are independent of *m*, i.e., biological replicate-independent while *u*_*ℓ*_2_*k*_ whose values are distinct between *k* = 1 and *k* = 2, i.e., distinct between Mock infection and SARS-CoV-2. These requirements support the fact that the identified singular value vectors are biologically relevant.

The next step was to identify *G*(*ℓ*_1_, *ℓ*_2_, *ℓ*_3_, *ℓ*_4_) with the largest absolute values given *ℓ*_1_, *ℓ*_2_, *ℓ*_3_, since such *ℓ*_4_ should be associated with *u*_*ℓ*_4_*i*_ similar to gene expression having *j*, *k*, *m* dependence represented by selected *u*_*ℓ*_1_*j*_, *u*_*ℓ*_2_*k*_, *u*_*ℓ*_3_*m*_. This enabled selection of *u*_*ℓ*_4_*i*_ used for gene selection. P-values, *P*_*i*_s, are attributed to *i*th gene using the following formula under the null hypothesis that *u*_*ℓ*_4_*i*_ obeys Gaussian distribution:

$$\begin{array}{c} P_{i}=P_{\chi^{2}}[>{\left(\frac{u_{\ell_{4}i}}{\sigma_{\ell_{4}}}\right)}^{2}] \end{array}$$ (2)

where *P*_*χ*^2^_[> *x*] is cumulative distribution of the *χ*^2^ distribution where the argument is larger than *x* and $\sigma_{\ell_{4}}$ is the standard deviation. Next, *P*_*i*_s were adjusted by Benjamini and Hochberg criterion [5] and genes associated with adjusted *P*-values less than 0.01 were selected as those whose gene expression is significantly associated with the assumed dependence upon *j*, *k*, *m*.

### 2.3 Enrichment analysis

Gene symbols of genes selected by TD-based unsupervised FE with significantly altered expression due to SARS-CoV-2 infection were uploaded to Enricher [7], which is a popular enrichment analysis server that evaluates the biological properties of genes based on enrichment analysis.

### 2.4 Differential expressed genes identification

Differential expressed genes (DEG) were identified by *t* test, sam [8] and limma [9]. Given *k*, for individual *i*s, *x*_*i*1*km*_ and *x*_*i*2*km*_ were compared. For *t* test and sam, normalized *x*_*ijkm*_ were compared. For limma, logarithmic values of raw *x*_*ijkm*_ were compared with excluding *i*s having zero *x*_*ijkm*_, since logarithmic values cannot be computed for negative or zero values. Since there are as small as three biological replicates, three replicates of each pair are compared with each other. Obtained *P*-values are adjusted by BH criterion and *i*th gene having adjusted *P*-values less than 0.01 are selected.

## 3 Results

### 3.1 Gene selection

**Fig 2:**
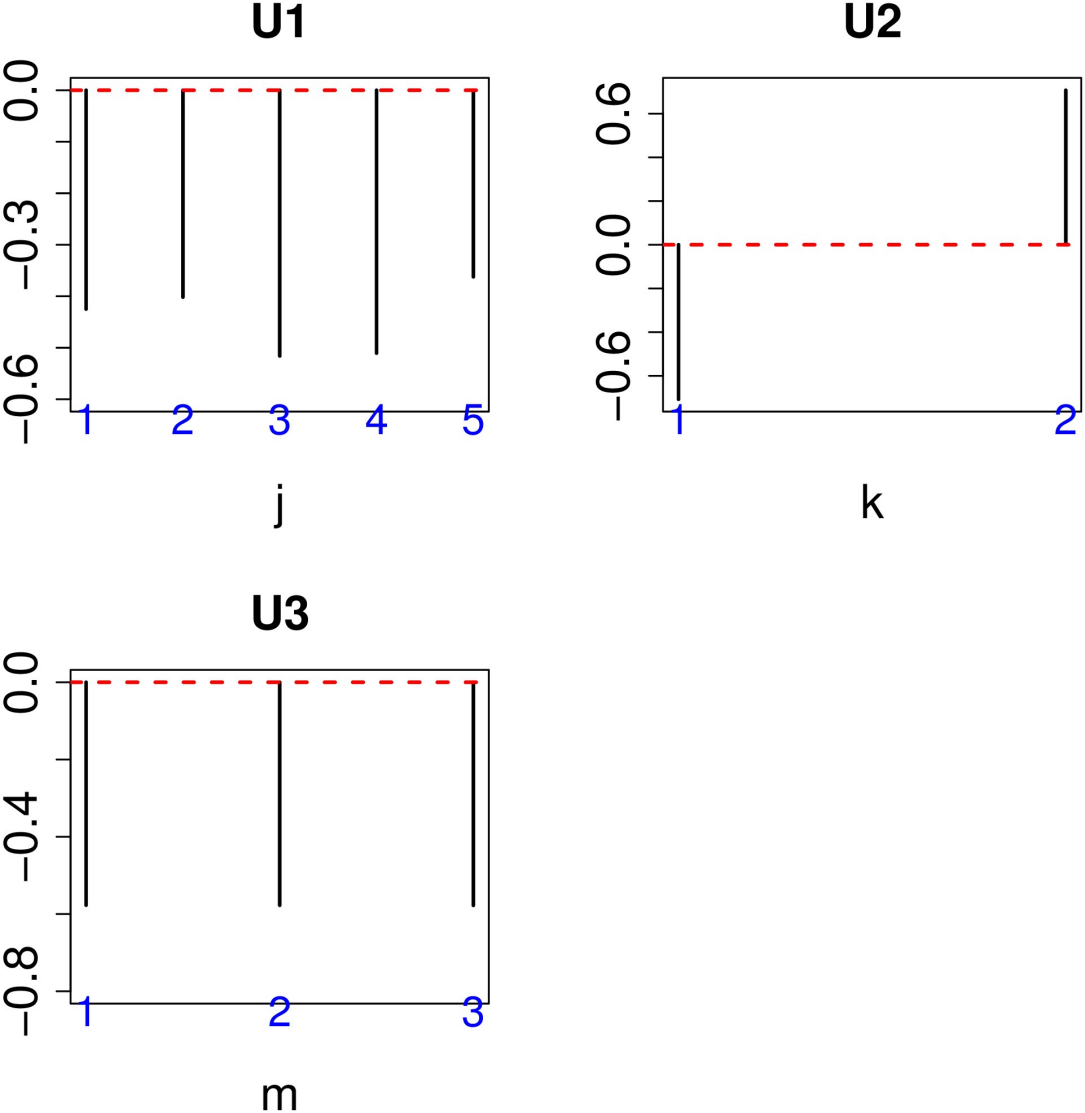
Singular value vectors obtained by the HOSVD algorithm. U1:*U*_1*j*_, U2:*U*_2*k*_, U3:*U*_1*m*_, See Materials and methods for the definitions of *j*, *k*, and *m*.

After identifying *ℓ*_1_ = 1, *ℓ*_2_ = 2, and *ℓ*_3_ = 1 based upon the criterion denoted in the Materials and Methods (Fig 2), we attempted to list *G*(1, 2, 1, *ℓ*_4_)s to select *ℓ*_4_ used for gene selection.

We found that *G*(1, 2, 1, 5) had the largest absolute value (Table 1). As a result, *u*_5*i*_ was employed to attribute *P*-values to gene *i* as shown in Eq (2). Finally, we selected 163 genes showing adjusted *P*-values less than 0.01 (Table 2).

**Table 1:** *G*(1, 2, 1, *ℓ*_4_)s computed by the HOSVD algorithm.

*ℓ*_4_	*G*(1, 2, 1, *ℓ*_4_)	*ℓ*_4_	*G*(1, 2, 1, *ℓ*_4_)
1	-21.409671	6	-12.388615
2	5.183297	7	8.437642
3	-21.426437	8	13.322888
4	10.030564	9	-1.850982
5	62.518121	10	9.211437

**Table 2:** One hundred and sixty-three genes selected by TD-based unsupervised FE.

ABCC3 ACE2 ACTB ACTG1 ACTN4 AHNAK AKAP12 AKR1B1 AKR1B10 AKR1C2 ALDH1A1 ALDH3A1 ALDOA AMIGO2 ANTXR1 ANXA2 ASNS ASPH ATF4 ATP1B1 C3 CALM2 CALR CD24 CFL1 CPLX2 CRIM1 CTGF CXCL5 CYP24A1 DCBLD2 DDIT4 DHCR24 EEF1A1 EEF2 EIF1 EIF4B EIF5A ENO1 ERBB2 EREG FADS2 FASN FDCSP FDPS FLNB FTH1 FTL G6PD GAPDH GAS5 GPX2 GSTP1 H1F0 HMGA1 HNRNPA2B1 HSP90AA1 HSP90AB1 HSPA8 ICAM1 IER3 IFIT2 IGFBP3 IGFBP4 ITGA2 ITGA3 ITGAV ITGB1 JUN KRT18 KRT19 KRT23 KRT5 KRT6A KRT7 KRT8 KRT81 LAMB3 LAMC2 LCN2 LDHA LIF LOXL2 MIEN1 MTHFD2 MYL6 NAMPT NAP1L1 NEAT1 NFKBIA NPM1 NQO1 OAS2 P4HB PABPC1 PFN1 PGK1 PKM PLAU PLOD2 PMEPA1 PPIA PPP1R15A PSAT1 PSMD3 PTMA RAI14 RNF213 RPL10 RPL12 RPL23 RPL26 RPL28 RPL3 RPL37 RPL4 RPL5 RPL7 RPL7A RPL9 RPS19 RPS20 RPS24 RPS27 RPS27A RPS3A RPS4X RPS6 S100A2 S100A6 SAT1 SCD SERPINA3 SERPINE1 SLC38A2 SLC7A11 SLC7A5 SPP1 SPTBN1 SQSTM1 STARD3 STAT1 STC2 TGFBI TGM2 TIPARP TMSB4X TNFAIP2 TOP2A TPI1 TPM1 TPT1 TRAM1 TUBA1B TUBB TUBB4B TXNIP TXNRD1 UBC VEGFA VIM YBX1 YWHAZ

### 3.2 Enrichment analysis

The selected 163 genes were uploaded to Enrichr (full list is available in S1 File) and we identified numerous enriched categories useful for follow-up analyses of the selected 163 genes and in *in silico* drug discovery as described below.

#### 3.2.1 Protein-protein interactions

The 163 selected proteins significantly interacted with numerous SARS-CoV virus proteins that play key roles in virus infection. Thus, our strategy successfully identified critical human genes associated with the coronavirus infection (S1 Table).

#### 3.2.2 Virus perturbations

Next, we examined whether the selected 163 genes significantly overlapped with genes whose expression was altered by infection with viruses other than SARS-CoV-2. We investigated “Virus Perturbations from GEO up” (S2 Table, full list is available in S1 File) and “Virus Perturbations from GEO down” (S3 Table, full list is available in S1 File). We found that SARS-CoV and SARS-BAtSRBD, which are coronaviruses mostly related to SARS-CoV-2, were highly enriched. This also suggests that our strategy is effective for identifying genes important in SARS-CoV-2 infection.

### 3.3 Drug discovery

Based upon the observations described above, we regarded the selected 163 proteins as representative of the SARS-CoV-2 infection process. Next, we evaluated drug candidate compounds by identifying those that significantly affected the expression of the selected 163 genes. For this, we investigated individual drug treatment-related categories in Enrichr.

#### 3.3.1 LINCS L1000 Chem Pert up/down

The first category investigated in Enrichr was “LINCS L1000 chem pert”. LINCS collected numerous cell lines treated with various drug compounds. Their altered expression profiles have been measured and stored in a public domain database. We found many drug compounds whose treatments significantly altered the expression of the selected 163 genes. Because the number of “hits” is too large to show here, tables are provided as supplementary tables. Selected drugs in this category are shown below. We identified many candidate drug compounds, indicating that our strategy is effective.

**C646**. C646 showed the second smallest (significant) *P*-value in “LINCS L1000 Chem Pert up” and had multiple hits (S4 Table). This agent was also reported to be a novel p300/CREB-binding protein-specific inhibitor of histone acetyltransferase which attenuates influenza A virus infection [10].

**Chelerythrine chloride**. Chelerythrine chloride exhibited the third and fifth smallest (significant) *P*-value in “LINCS L1000 Chem Pert up” and had multiple hits (S5 Table). It is known to exhibit pharmacological inhibition of protein kinase C reduces West Nile virus replication (See Fig,1 [11]).

**Canertinib**. Canertinib exhibited the sixth smallest (significant) *P*-value in “LINCS L1000 Chem Pert up” and had multiple hits (S6 and S7 Tables). It shows antiviral chemotherapy effects and controls poxvirus infections by inhibiting cellular signal transduction [12].

**BX-795**. BX-795 has the 11th smallest (significant) *P*-value in “LINCS L1000 Chem Pert up” and had multiple hits (S8 Table). BX-795 inhibits HSV-1 and HSV-2 replication by blocking the JNK/p38 pathways without interfering with PDK1 activity in host cells [13]. Su et al [13] also suggested SARS-CoV as a target of BX-795.

**Sorafenib**. Sorafenib showed the 12th smallest (significant) *P*-value in “LINCS L1000 Chem Pert up” and had multiple hits (S9 Table). Sorafenib impedes Rift Valley fever virus egress by inhibiting valosin-containing protein function in the cellular secretory pathway [14].

**QL-X-138**. QL-X-138 displayed the smallest (significant) *P*-value in “LINCS L1000 Chem Pert down” and had multiple hits (S10 and S11 Tables). QL-XII-138 inhibits Dengue virus (see Fig 3 [15]).

**Radicicol**. Radicicol showed the second smallest (significant) *P*-value in “LINCS L1000 Chem Pert down” and had multiple hits (S12 and S13 Tables). Antiviral activity and RNA polymerase of radicicol is degradation following Hsp90 inhibition in a range of negative-strand viruses [16]. Radicicol also preferentially reduces HCV release, although radicicol does not affect its infectivity [17]. Because other Hsp90 inhibitors are effective against coronavirus [18], radicidol is also thought to be effective for treating SARS-CoV-2.

**A-443654**. A-443654 showed the fourth smallest (significant) *P*-value in “LINCS L1000 Chem Pert down” and had multiple hits (S14 and S15 Tables). Jeong and Ahn found that viral replication of HBV in infected or transfected hepatoma cells was markedly inhibited by treatment with A-443654 [19], a specific inhibitor of Akt. As the SARS-CoV membrane protein also induces apoptosis by modulating the Akt survival pathway [20], A-443654 may be an effective drug for treating COVID-19. The “PI3K-Akt signaling pathway” was the fourth most significant pathway (adjusted *P* = 3.97×10^−7^, overlap is 17/354) in the “KEGG 2019 Human” category of Enrichr (full list is available in S1 File) to which the 163 selected genes were uploaded.

**CGP-60474**. CGP-60474 had the fifth smallest (significant) *P*-value in “LINCS L1000 Chem Pert down” and multiple hits (S16 and S17 Tables). CGP-60474 is also a repurposed drug that was used to treat lung injury in COVID-19 in an independent *in silico* study [21].

**Alvocidib**. Alvocidib showed the sixth smallest (significant) *P*-value in “LINCS L1000 Chem Pert down” and had multiple hits (S18 and S19 Tables). Alvocidib, a kinase inhibitor, was repurposing as an antiviral agent to control influenza A virus replication [22].

**Mitoxantrone**. Mitoxantrone exhibited the 20th smallest (significant) *P*-value in “LINCS L1000 Chem Pert down” and had multiple hits (S20 and S21 Tables). Mitoxantrone inhibits Vaccinia virus replication by blocking virion assembly [23].

**QL-XII-47**. QL-XII-47 showed the 22nd smallest (significant) *P*-value in “LINCS L1000 Chem Pert down” and had multiple hits (S22 and S23 Tables). QL-XII-47’s inhibition of Zika virus, West Nile virus, hepatitis C virus, and poliovirus have been reported previously [15].

**Geldanamycin**. Geldanamycin showed the 25th smallest (significant) *P*-value in “LINCS L1000 Chem Pert down” and had multiple hits (S24 and S25 Tables). Similar to radicicol as described above, the antiviral activity and RNA polymerase of radicicol involves degradation following Hsp90 inhibition in a range of negative-strand viruses [16]. These observations for radicicol are also applicable to geldanamycin.

#### 3.3.2 Drug perturbations from GEO

Although we successfully identified numerous drug candidate compounds, it would also be useful to identify more candidates in other categories to confirm the effectiveness of our strategy. Thus, we next investigate “Drug Perturbations from GEO up/down” categories. As described below, we found numerous drug candidate compounds within these data sets (S26 Table).

**Fluticasone**. Effect of fluticasone propionate on virus-induced airway inflammation and antiviral immune responses in mice [24].

**Atorvastatin**. Atorvastatin restricts the ability of influenza virus to generate lipid droplets and severely suppresses virus replication [25].

**Quercetin**. Quercetin was reported to inhibit the cell entry of SARS-CoV-2 [26] and was included in the list of candidate compounds for SARS-CoV-2 screened by an *in silico* method [27].

**Motexafin gadolinium**. Motexafin gadolinium was reported to selectively induce apoptosis in HIV-1-infected CD4+ T helper cells [28].

**Trovafloxacin**. Simian virus 40 large T antigen helicase activity was inhibited by fluoroquinolone, trovafloxacin [29].

**Doxycycline**. Antiviral activity of doxycycline against vesicular stomatitis virus was observed *in vitro* [30].

#### 3.3.3 Drug matrix

To further confirm the independency of our findings based on the data sets used, we also examined the “Drug Matrix” category (S27 Table, the full list is available in S1 File). As we found some hits, our method can robustly identify promising drug candidate compounds.

**Meloxicam**. Meloxicam is known to exert cytotoxic and antiproliferative activities towards virus-transformed tumor cells [31], including myelocytomatosis virus and Rous sarcoma virus. Myelocytomatosis virus is a retrovirus, which is an enveloped, negative-sense, single-stranded RNA virus, whereas Rous sarcoma virus is an enveloped, positive-sense, single-stranded RNA virus.

**Gentamicin**. Although gentamicin is known to be a bactericidal antibiotic, it also exhibits antiviral activity (Table 3 [32]).

**Dibromochloromethane**. Dibromochloromethane was announced as a possible antiviral drug by the Agency for Toxic Substances and Disease Registry (PUBLIC HEALTH STATEMENT Bromoform and Dibromochloromethane CAS#: 75-25-2 and 124-48-1, 2005).

### 3.4 Comparison with *in silico* drug discovery

Finally, we compared our results with those of other drugs identified *in silico*. As expected, some overlap was observed.

#### 3.4.1 Comparison with Wu et al. [33]

We found multiple hits, which are summarized in S28 Table; Wu et al. [33] identified 29 potential PLpro inhibitors, 27 potential 3CLpro inhibitors, and 20 potential RdRp inhibitors from the ZINC drug database, and identified 13 potential PLpro inhibitors, 26 potential 3Clpro inhibitors, and 20 Potential RdRp inhibitors from their in-house natural product database. Doxycycline was among both the potential PLpro and 3CLpro inhibitors; ascorbic acid and isotretinoin were among the potential PLpro inhibitors; pioglitazone was among the potential 3CLpro inhibitors; and cortisone and tibolone were included as potential RdRp inhibitors from the ZINC drug database. These multiple hits further support the suitability of our strategy.

#### 3.4.2 Comparison with Ubani et al. [27]

Ubani et al. [27] screened a library of 22 phytochemicals with antiviral activity obtained from the PubChem database for activity against the spike envelope glycoprotein and main protease of SARS-CoV-2. Among these, we found only one hit that overlapped with our screened out drugs, which was quercetin (S29 Table).

## 4 Discussion and conclusion

In this study, we proposed an advanced unsupervised learning method working in 4D tensors for identifying numerous promising drug candidate compounds for treating COVID-19 infection. The proposed method works by applying TD-based unsupervised FE to gene expression profiles of multiple lung cancer cell lines infected by SARS-CoV-2. We successfully identified 163 human genes predicted to be involved in the SARS-CoV-2 infection process. By uploading these selected 163 genes to Enrichr, we found that numerous drug compounds significantly altered expression of the genes.

Various analyses demonstrated that our results are robust. First, in a previous study [34] in which we employed a similar strategy to understand the infectious process of mouse hepatitis virus, a well-studied model CoV, we also identifies numerous drug candidate compounds in “DrugMatrix” and “Drug Pert from GEO up/down” categories in Enrichr. Although these drug compounds identified in the previous study were not always identified as top-ranked categories in this study (S26 and S27 Tables), most were significant. For example, in the “Drug Matrix” category, the identified drugs in the previous study were primaquine, meloxicam, cytarabine, pyrogallol, catechol, and neomycin. Among these six drugs, none, except for meloxicam, were ranked within the top ten (S27 Table), but still significantly affected the expression of the selected 163 genes in this study (S30 Table). In the “Drug Pert from GEO up/down” category, the identified drugs in the previous study were fenretinide, pioglitazone, quercetin, decitabine, troglitazone, and motexafin gadolinium. Among these, only quercetin and motexafin gadolinium were identified in the present study (S26 Table), but other four drugs still significantly affected the expression of the selected 163 genes (S31 Table). Additionally, doxycycline, ascorbic acid, isotretinoin, pioglitazone, cortisone, tibolone, and quercetin were identified in the comparison with two other *in slico* studies. These drugs were also identified in the comparison between the present study and other *in slico* studies (S28 and S29 Tables). These overlapping results with the previous study suggest that our strategy is quite robust.

These results are also thought to be biologically sound. For example, Although A-443654 is inhibitor of Akt, which is important for SARS-CoV infection (see above). Radicicol and geldanamycin inhibit Hsp90. The importance of inhibition of Hsp90 was reported for treating patients with COVID-19 has been reported previously [35]. Although we could not identify all biological meanings of the identified drugs, these two examples suggest that the results are biologically sound.

One may wonder if the detection of PPI in SARS-CoV reported in S1 Table is meaningful, as SARS-CoV does differ from SARS-CoV-2. In order to confirm if our identified 163 genes also significantly overlapped with PPI in SARS-CoV-2, we compared the genes with those identified to be interacting with SARS-CoV-2 proteins [36] (S32 Table). The 163 genes identified in this study turned out to be highly coincident with human genes reported to be interacting with SARS-CoV-2 proteins (S33 Table). *P*-values reported in S33 Table were computed by Fisher exact test between 163 genes and human genes reported to be interacting with SARS-CoV-2 proteins in S32 Table. It is obvious that the identified 163 genes are significantly overlapping with genes reported to be interacting with SARS-CoV-2 proteins. Thus, the PPI detected in this study (S1 Table) is not accidental but reliable.

Next we compared our drug repositioning proposals based on DrugMatrix, GEO and LINCS in Enrichr (provided as S1 File) with the drugs identified for SARS-CoV-2 in another study [37]. Among 142 drugs identified by Zhou et al [37], as many as 43 drugs were found to significantly affect 163 genes in at least one experiment within either DrugMatrix, GEO, or LINCS in Enrichr (S34 Table). Thus, our proposal of drug repositioning is also reliable.

This study might be considered to be purely incremental, as the methods employed in this study other than TD based unsupervised FE are simply comparisons with other studies and databases. However, we believe it is the opposite. Using our methods, although we could identify very limited number of genes (163 genes), the small number of identified genes widely overlapped with at least three categories (DrugMatrix, GEO, and LINCS) in Enricher, two *in silico* studies [27, 33] as well as two very recent studies that specifically targeted SARS-CoV-2 [36, 37]. Comparisons with external researches rarely give good results. Therefore, the result that our small number of 163 genes was coincident with a large number of independent research suggests the superiority of our strategy. To our knowledge, no other strategies can identify such small number of genes that are significantly coincident with large number of studies.

One might also ask why we did not employ simpler approaches like identification of gene expressed distinctly between mock and infected cells (DEG). Nevertheless, this kind of approach forced us to identify DEGs in each cell line and allowed us to select intersections between those identified in each of as many as five cell lines. Considering that intersection might decrease the number of DEGs or might result in no intersections, if our integrated approach works well, there are no reasons to seek DEGs in five cell lines one by one.

Another possible concern might be that we did not distinguish between upregluation and downregulation when we selected genes, but simply considered overlaps of genes associated with altered expression between SARS-CoV-2 infection and drug treatment. In this sense, there could be a possibility that some selected drugs are not opposed to infection but rather accelerate it. However, the tissues and cell lines that were treated with the drugs showed a wide range and sometimes upregulation and downregulation differ between distinct tissues and cell lines. The purpose of this study was to screen candidate compounds, and we did not focus on strict coincidence between upreguation and downregulation, as too strict a criterion might overlook a useful candidate drug compound.

Our strategy has some advantages over LBDD and SBDD. We do not need any list of drugs known to be effective to SARS-CoV-2. As we presently do not have any known effective drugs for SARS-CoV-2, LBDD strategy can be hardly performed. In contrast to SBDD, which requires massive computational resources like supercomputer, our method is light weighted and can be performed with a standard computational server that can be purchased even in a small laboratory. Thus, we believe that our strategy is superior to both LBDD and SBDD for drug repositioning.

We noticed that ivermectin is included in the hits in DrugMatrix category in Enrichr (Table 3). Ivermectin was recently reported to inhibit the replication of SARS-CoV-2 *in vitro* [38]. As ivermectin was first invented as anti-parasite drug, no previous supervised *in silico* approach considered it. To our knowledge, this is the first report of an *in silico* approach that can detect ivermectin as a possible SARS-CoV-2 drug. This suggests the effectiveness of our unsupervised approach.

**Table 3:** Ivermectin detected in DrugMatrix category in Enrichr.

Term	Overlap	P-value	Adjusted P-value
Ivermectin-7.5 mg/kg in CMC-Rat-Liver-1d-dn	12/277	2.98E-06	9.93E-06
Ivermectin-7.5 mg/kg in CMC-Rat-Liver-5d-dn	12/289	4.60E-06	1.44E-05
Ivermectin-7.5 mg/kg in CMC-Rat-Liver-3d-dn	11/285	2.29E-05	5.56E-05
Ivermectin-7.5 mg/kg in CMC-Rat-Liver-1d-up	10/323	3.28E-04	5.39E-04
Ivermectin-7.5 mg/kg in CMC-Rat-Liver-5d-up	8/311	4.06E-03	5.10E-03
Ivermectin-7.5 mg/kg in CMC-Rat-Liver-3d-up	8/315	4.38E-03	5.46E-03

Finally, we would like to explain why our method (1) is applicable in drug discovery and (2) outperforms other conventional methods. At first, most of gene expression based *in silico* drug discovery methods are supervised methods [39, 40] that require known target-drug relations or drug-disease relations, which are not available for SARS-CoV-2. Thus, no supervised methods can be applicable to the present study. On the other hand, for other unsupervised approaches [41, 42], the earlier studies selected genes specific to diseases as key features. They also selected drugs that affect the selected genes. Thus, the basic strategy is similar to ours. The question remained whether we can select limited number of genes whose expression is altered because of SARS-CoV-2 infection. To see superiority of TD based unsupervised FE that can select as few as 163 genes effective to selected drugs, we applied *t* test, sam [8], and limma [9] to pairwise comparisons between individual control and infected cell lines (Table 4). Notably, none of these three methods were effective. The *t* test selected less than or equal to one gene for three out of five cell lines. While no gene was selected by SAM for all of five cell lines, limma identified almost all genes as DEG. As long as performance of other unsupervised methods depends upon the successful selection of DEG as disease signature, other unsupervised methods that did not employ TD based unsupervised FE are unlikely to identify effective drugs better than the present study. Thus, based on our results, we can conclude that the employment of TD based unsupervised FE for selecting genes is instrumental for a successful unsupervised gene expression based drug discovery.

**Table 4:** DEG identifications between control and infectious cell lines using *t* test, SAM, and limma. Genes associated with adjusted *P*-values less than 0.01 are selected as DEG.

	*t* test		SAM		limma	
	*P* ≥ 0.01	*P* < 0.01	*P* ≥ 0.01	*P* < 0.01	*P* ≥ 0.01	*P* < 0.01
Calu3	21754	43	21797	0	42	13380
NHBE	21797	0	21797	0	41	13328
A549						
MOI 0.2	21797	0	21797	0	50	13867
MOI 2.0	21472	325	21797	0	15	13823
ACE2 expressed	21796	1	21797	0	111	11403

## Supporting information

S1 Table Virus protein-protein interaction. Virus proteins that significantly interact with the 163 genes selected by TD based unsupervised FE and enriched by “Virus-Host PPI P-HIPSTer 2020” in Enrichr.
(PDF)

S2 Table Upregulated genes due to SARS-CoV-2 infection. Genes whose expression is altered by SARS-CoV-2-related viruses that significantly interact with the 163 genes selected by TD based unsupervised FE and enriched by “Virus Perturbations from GEO up” in Enrichr.
(PDF)

S3 Table Downregulated genes due to SARS-CoC-2 infection. Genes whose expression was altered by SARS-CoV-2-related viruses that significantly interact with the 163 genes selected by TD-based unsupervised FE and enriched by “Virus Perturbations from GEO down” in Enrichr.
(PDF)

S4 Table C646 in “LINCS L1000 Chem Pert up/down”. C646 significantly affects the expression of the selected 163 genes as evident in the “LINCS L1000 Chem Pert up/down” category in Enrichr. The last number after the—is dose density.
(PDF)

S5 Table Chelerythrine chlorid in “LINCS L1000 Chem Pert up/down”. Chelerythrine chlorid significantly affects the expression of the selected 163 genes as evident in the “LINCS L1000 Chem Pert up/down” category in Enrichr. The last number after the—is dose density.
(PDF)

S6 Table Canertinib in “LINCS L1000 Chem Pert up”. Canertinib significantly affects the expression of the selected 163 genes as evident in the “LINCS L1000 Chem Pert up” category in Enrichr. The last number after the—is dose density.
(PDF)

S7 Table Canertinib in “LINCS L1000 Chem Pert down”. Canertinib significantly affects the expression of the selected 163 genes as evident in the “LINCS L1000 Chem Pert down” category in Enrichr. The last number after the—is dose density.
(PDF)

S8 Table BX-795 in “LINCS L1000 Chem Pert up/down”. BX-795 significantly affects the expression of the selected 163 genes as evident in the “LINCS L1000 Chem Pert up/down” category in Enrichr. The last number after the—is dose density.
(PDF)

S9 Table Sorafenib in “LINCS L1000 Chem Pert up”. Sorafenib significantly affects the expression of the selected 163 genes as evident in the “LINCS L1000 Chem Pert up” category in Enrichr. The last number after the—is dose density.
(PDF)

S10 Table QL-X-138 in “LINCS L1000 Chem Pert up”. QL-X-138 significantly affects the expression of the selected 163 genes as evident in the “LINCS L1000 Chem Pert up” category in Enrichr. The last number after the—is dose density.
(PDF)

S11 Table QL-X-138 in “LINCS L1000 Chem Pert down”. QL-X-138 significantly affects the expression of the selected 163 genes as evident in the “LINCS L1000 Chem Pert down” category in Enrichr. The last number after the—is dose density.
(PDF)

S12 Table Radicicol in “LINCS L1000 Chem Pert up”. Radicicol significantly affects the expression of the selected 163 genes due to “LINCS L1000 Chem Pert up” category in Enrichr. The last number after the—is dose density.
(PDF)

S13 Table Radicicol in “LINCS L1000 Chem Pert down”. Radicicol significantly affects the expression of the selected 163 genes due to “LINCS L1000 Chem Pert up” category in Enrichr. The last number after the—is dose density.
(PDF)

S14 Table A-443654 in “LINCS L1000 Chem Pert up”. A-443654 significantly affects the expression of the selected 163 genes as evident in the “LINCS L1000 Chem Pert up” category in Enrichr. The last number after the—is dose density.
(PDF)

S15 Table A-443654 in “LINCS L1000 Chem Pert up”. A-443654 significantly affects the expression of the selected 163 genes as evident in the “LINCS L1000 Chem Pert up” category in Enrichr. The last number after the—is dose density.
(PDF)

S16 Table CGP-60474 in “LINCS L1000 Chem Pert up”. CGP-60474 significantly affects the expression of the selected 163 genes due to “LINCS L1000 Chem Pert up” category in Enrichr. The last number after the—is dose density.
(PDF)

S17 Table CGP-60474 in “LINCS L1000 Chem Pert down”. CGP-60474 significantly affects the expression of the selected 163 genes due to “LINCS L1000 Chem Pert down” category in Enrichr. The last number after the—is dose density.
(PDF)

S18 Table Alvocidib in “LINCS L1000 Chem Pert up”. Alvocidib significantly affects the expression of the selected 163 genes as evident in the “LINCS L1000 Chem Pert up” category in Enrichr. The last number after the—is dose density.
(PDF)

S19 Table Alvocidib in “LINCS L1000 Chem Pert down”. Alvocidib significantly affects the expression of the selected 163 genes as evident in the “LINCS L1000 Chem Pert down” category in Enrichr. The last number after the—is dose density.
(PDF)

S20 Table Mitoxantrone in “LINCS L1000 Chem Pert up”. Mitoxantrone significantly affects the expression of the selected 163 genes as evident in the “LINCS L1000 Chem Pert up” category in Enrichr. The last number after the—is dose density.
(PDF)

S21 Table Mitoxantrone in “LINCS L1000 Chem Pert down”. Mitoxantrone significantly affects the expression of the selected 163 genes as evident in the “LINCS L1000 Chem Pert down” category in Enrichr. The last number after the—is dose density.
(PDF)

S22 Table QL-XII-47 in “LINCS L1000 Chem Pert up”. QL-XII-47 significantly affects the expression of the selected 163 genes as evident in the “LINCS L1000 Chem Pert up” category in Enrichr. The last number after the—is dose density.
(PDF)

S23 Table QL-XII-47 in “LINCS L1000 Chem Pert down”. QL-XII-47 significantly affects the expression of the selected 163 genes as evident in the “LINCS L1000 Chem Pert down” category in Enrichr. The last number after the—is dose density.
(PDF)

S24 Table Geldanamycin in “LINCS L1000 Chem Pert up”. Geldanamycin significantly affects the expression of the selected 163 genes as evident in the “LINCS L1000 Chem Pert up” category in Enrichr. The last number after the—is dose density.
(PDF)

S25 Table Geldanamycin in “LINCS L1000 Chem Pert down”. Geldanamycin significantly affects the expression of the selected 163 genes as evident in the “LINCS L1000 Chem Pert down” category in Enrichr. The last number after the—is dose density.
(PDF)

S26 Table Enrichment in “Drug Perturbations from GEO up/down”. Genes whose expression is altered by SARS-CoV-2-related viruses that significantly interact with the 163 genes selected by TD-based unsupervised FE and enriched by “Drug Perturbations from GEO up/down” in Enrichr.
(PDF)

S27 Table Enrichment in “Drug Matrix”. Genes whose expression is altered by SARS-CoV-2-related viruses that significantly interact with the 163 genes selected by TD-based unsupervised FE and enriched by “Drug Matrix” in Enrichr.
(PDF)

S28 Table Comparison with *in silico*: I. List of *in silico* screened drugs [33] whose target genes were also enriched in the 163 genes selected by TD-based unsupervised FE.
(PDF)

S29 Table Comparison with *in silico*: II. List of *in silico* screened drugs [27] whose target genes are also among the 163 genes selected by TD based unsupervised FE.
(PDF)

S30 Table Comparison with “DrugMatrix” in the previous study. Five Drugs ranked within top 10 in the previous study but not in the present study in “DrugMatrix” category in Enrichr. They were still significantly enriched for the selected 163 genes. If there were more than ten hits, they were omitted.
(PDF)

S31 Table Comparison with “Drug Pert from GEO up/down” in the previous study. Four Drugs ranked within top 10 in the previous study but not in the present study in “Drug Pert from GEO up/down” category in Enrichr. They were still significantly enriched toward the selected 163 genes.
(PDF)

S32 Table Protein protein interaction with SARS-CoV-2. The number of human proteins reported to interact with listed SARS-CoV-2 proteins [36].
(PDF)

S33 Table Coincidence with SARS-CoV-2 PPI. Coincidence between 163 genes and human proteins whose numbers are reported in S32 Table.
(PDF)

S34 Table Comparison with drugs previously annotated for SARS-CoV-2. Number of experiments associated with adjusted *P*-values in various Enrichr categories for the drugs identified in another study [37].
(PDF)

S1 File Results of Enrichr. Full list of various enrichment analyses available in supplementary tables.
(XLSX)
