# Supplementary material for: A new advanced in silico drug discovery method for novel coronavirus (SARS-CoV-2) with tensor decomposition-based unsupervised feature extraction
Source: PLoS One. 2020 Sep 11;15(9):e0238907. doi: 10.1371/journal.pone.0238907 (PMC7485840; doi:10.1371/journal.pone.0238907)
Supplement: S1 Table — Virus proteins that significantly interact with the 163 genes selected by TD based unsupervised FE and enriched by “Virus-Host PPI P-HIPSTer 2020” in Enrichr. (PDF) [file pone.0238907.s001.pdf]

S1 Table: Virus proteins that significantly interact with the 163 genes selected by TD based unsupervised FE and enriched by "Virus-Host PPI P-HIPSTer 2020" in Enrichr

Term	Overlap	P-value	Adjusted P-value
SARS coronavirus excised_polyprotein 1..4369 (gene: orf1ab)	12/194	6.67×10^{-8}	2.38×10^{-6}
SARS coronavirus P2 full_polyprotein 1..4382	12/198	8.35×10^{-8}	2.76×10^{-6}
SARS coronavirus hypothetical protein sars9b	4/17	9.31×10^{-6}	7.57×10^{-5}
SARS coronavirus P2 hypothetical protein sars9b	4/17	9.31×10^{-6}	7.562×10^{-5}
SARS coronavirus Tor2 Orf13	4/17	9.31×10^{-6}	7.55×10^{-5}
SARS coronavirus nsp7-pp1a/pp1ab (gene: orf1ab)	5/36	1.038×10^{-5}	8.18×10^{-5}
SARS coronavirus 3C-like proteinase (gene: orf1ab)	4/19	1.49×10^{-5}	1.10×10^{-4}
SARS coronavirus nucleocapsid protein (gene: N)	4/29	8.61×10^{-5}	4.23×10^{-4}
SARS coronavirus P2 nucleocapsid protein	4/29	8.61×10^{-5}	4.23×10^{-4}
SARS coronavirus Tor2 nucleocapsid protein	4/29	8.61×10^{-5}	4.22×10^{-4}
SARS coronavirus nsp4-pp1a/pp1ab (gene: orf1ab)	3/16	2.75×10^{-4}	9.89×10^{-4}
SARS coronavirus formerly known as growth-factor-like protein (gene: orf1ab)	3/17	3.32×10^{-4}	1.14×10^{-3}
SARS coronavirus nsp8-pp1a/pp1ab (gene: orf1ab)	4/45	4.88×10^{-4}	1.50×10^{-3}
SARS coronavirus leader protein (gene: orf1ab)	3/20	5.47×10^{-4}	1.61×10^{-3}
SARS coronavirus RNA-dependent RNA polymerase (gene: orf1ab)	2/9	2.28×10^{-3}	5.26×10^{-3}
SARS coronavirus P2 spike glycoprotein precursor	4/71	2.70×10^{-3}	6.08×10^{-3}
SARS coronavirus nsp3-pp1a/pp1ab (gene: orf1ab)	5/118	2.82×10^{-3}	6.34×10^{-3}
SARS coronavirus E2 glycoprotein precursor (gene: S)	4/72	2.84×10^{-3}	6.38×10^{-3}
SARS coronavirus Tor2 spike glycoprotein	4/72	2.84×10^{-3}	6.38×10^{-3}
SARS coronavirus 2-O-ribose methyltransferase (2-o-MT) (gene: orf1ab)	2/11	3.45×10^{-3}	7.26×10^{-3}
SARS coronavirus hypothetical protein sars7a	3/38	3.63×10^{-3}	7.59×10^{-3}
SARS coronavirus P2 hypothetical protein sars7a	3/38	3.63×10^{-3}	7.58×10^{-3}
SARS coronavirus Tor2 Orf8	3/38	3.63×10^{-3}	7.58×10^{-3}
SARS coronavirus nsp9-pp1a/pp1ab (gene: orf1ab)	2/13	4.85×10^{-3}	9.45×10^{-3}
SARS coronavirus nsp13-pp1ab (ZD, NTPase/HEL; RNA (gene: orf1ab)	2/14	5.63×10^{-3}	1.06×10^{-2}
SARS coronavirus Tor2 replicase 1AB	4/108	1.18×10^{-2}	1.94×10^{-2}
SARS coronavirus P2 full_polyprotein 1..7073	4/109	1.22×10^{-2}	2.00×10^{-2}
