# Supplementary material for: A new advanced in silico drug discovery method for novel coronavirus (SARS-CoV-2) with tensor decomposition-based unsupervised feature extraction
Source: PLoS One. 2020 Sep 11;15(9):e0238907. doi: 10.1371/journal.pone.0238907 (PMC7485840; doi:10.1371/journal.pone.0238907)
Supplement: S2 Table — Genes whose expression is altered by SARS-CoV-2-related viruses that significantly interact with the 163 genes selected by TD based unsupervised FE and enriched by “Virus Perturbations from GEO up” in Enrichr. (PDF) [file pone.0238907.s002.pdf]

S2 Table: Genes whose expression is altered by SARS-CoV-2-related viruses that significantly interact with the 163 genes selected by TD based unsupervised FE and enriched by "Virus Perturbations from GEO up" in Enrichr

Term	Overlap	P-value	Adjusted P-value
SARS-BatSRBD 48Hour GSE47960	11/300	3.66×10^{-5}	1.48×10^{-3}
SARS-CoV 12Hour GSE17400	11/300	3.66×10^{-5}	1.31×10^{-3}
SARS-CoV 48Hour GSE47961	11/300	3.66×10^{-5}	1.18×10^{-3}
icSARS CoV 54Hour GSE37827	11/300	3.66×10^{-5}	1.08×10^{-3}
SARS-CoV MA15 Day2 GSE49263	10/300	1.82×10^{-4}	3.45×10^{-3}
SARS-CoV 60Hour GSE47960	10/300	1.82×10^{-4}	3.26×10^{-3}
SARS-CoV 96Hour GSE47961	10/300	1.82×10^{-4}	3.09×10^{-3}
SARS-ddORF6 24Hour GSE47961	10/300	1.82×10^{-4}	2.93×10^{-3}
SARS-BatSRBD 36Hour GSE47960	9/300	8.14×10^{-4}	1.05×10^{-2}
SARS-CoV MA15 Day4-C57BL-6 GSE40824	9/300	8.14×10^{-4}	1.01×10^{-2}
SARS-dORF6 72Hour GSE47960	9/300	8.14×10^{-4}	9.73×10^{-3}
SARS-ddORF6 72Hour GSE47961	9/300	8.14×10^{-4}	9.39×10^{-3}
SARS-BatSRBD 84Hour GSE47961	8/300	3.27×10^{-3}	2.46×10^{-2}
SARS-CoV MA15 Day2 GSE49262	8/300	3.27×10^{-3}	2.40×10^{-2}
SARS-CoV MA15 Day4-PFU-10^5 GSE33266	8/300	3.27×10^{-3}	2.35×10^{-2}
SARS-dORF6 84Hour GSE47962	8/300	3.27×10^{-3}	2.30×10^{-2}
cSARS Bat SRBD 24Hour GSE37827	8/300	3.27×10^{-3}	2.25×10^{-2}
cSARS Bat SRBD 60Hour GSE37827	8/300	3.27×10^{-3}	2.20×10^{-2}
icSARS CoV 0Hour GSE37827	8/300	3.27×10^{-3}	2.16×10^{-2}
icSARS CoV 48Hour GSE37827	8/300	3.27×10^{-3}	2.11×10^{-2}
