# Supplementary material for: A new advanced in silico drug discovery method for novel coronavirus (SARS-CoV-2) with tensor decomposition-based unsupervised feature extraction
Source: PLoS One. 2020 Sep 11;15(9):e0238907. doi: 10.1371/journal.pone.0238907 (PMC7485840; doi:10.1371/journal.pone.0238907)
Supplement: S3 Table — Genes whose expression was altered by SARS-CoV-2-related viruses that significantly interact with the 163 genes selected by TD-based unsupervised FE and enriched by “Virus Perturbations from GEO down” in Enrichr. (PDF) [file pone.0238907.s003.pdf]

S3 Table: Genes whose expression was altered by SARS-CoV-2-related viruses that significantly interact with the 163 genes selected by TD-based unsupervised FE and enriched by “Virus Perturbations from GEO down” in Enrichr

Term	Overlap	P-value	Adjusted P-value
SARS-CoV 0Hour GSE47961	14/300	1.76×10^{-7}	1.42×10^{-5}
SARS-ddORF6 0Hour GSE47961	10/300	1.82×10^{-4}	4.51×10^{-3}
SARS-BatSRBD 96Hour GSE47960	9/300	8.14×10^{-4}	1.38×10^{-2}
SARS-CoV 24Hour GSE17400	9/300	8.14×10^{-4}	1.31×10^{-2}
cSARS Bat SRBD 60Hour GSE37827	9/300	8.14×10^{-4}	1.25×10^{-2}
icSARS CoV 48Hour GSE37827	9/300	8.14×10^{-4}	1.19×10^{-2}
SARS-CoV MA15 Day4-PFU-10^2 GSE33266	8/300	3.27×10^{-3}	3.11×10^{-2}
