# Supplementary material for: A new advanced in silico drug discovery method for novel coronavirus (SARS-CoV-2) with tensor decomposition-based unsupervised feature extraction
Source: PLoS One. 2020 Sep 11;15(9):e0238907. doi: 10.1371/journal.pone.0238907 (PMC7485840; doi:10.1371/journal.pone.0238907)
Supplement: S4 Table — C646 significantly affects the expression of the selected 163 genes as evident in the “LINCS L1000 Chem Pert up/down” category in Enrichr. The last number after the—is dose density. (PDF) [file pone.0238907.s004.pdf]

S4 Table: C646 significantly affects the expression of the selected 163 genes as evident in the "LINCS L1000 Chem Pert up/down" category in Enrichr. The last number after the - is dose density.

Term	Overlap	P-value	Adjusted P-value
LINCS L1000 Chem Pert up			
LJP008 HEPG2 24H-C646-10	21/104	1.04×10^{-23}	1.72×10^{-19}
LJP008 PC3 24H-C646-10	11/141	2.22×10^{-8}	9.95×10^{-7}
LJP008 HCC515 24H-C646-10	8/63	4.34×10^{-8}	1.75×10^{-6}
LJP008 A549 24H-C646-10	6/51	3.55×10^{-6}	7.05×10^{-5}
LJP008 MCF7 24H-C646-10	3/45	5.87×10^{-3}	3.24×10^{-2}
LINCS L1000 Chem Pert down			
LJP008 HA1E 24H-C646-10	7/96	1.36×10^{-5}	2.11×10^{-4}
LJP008 PC3 24H-C646-10	4/59	1.37×10^{-3}	1.01×10^{-2}
LJP008 A375 24H-C646-10	4/80	4.16×10^{-3}	2.53×10^{-2}
LJP008 HEPG2 24H-C646-10	4/81	4.35×10^{-3}	2.62×10^{-2}
