# Supplementary material for: A new advanced in silico drug discovery method for novel coronavirus (SARS-CoV-2) with tensor decomposition-based unsupervised feature extraction
Source: PLoS One. 2020 Sep 11;15(9):e0238907. doi: 10.1371/journal.pone.0238907 (PMC7485840; doi:10.1371/journal.pone.0238907)
Supplement: S5 Table — Chelerythrine chlorid significantly affects the expression of the selected 163 genes as evident in the “LINCS L1000 Chem Pert up/down” category in Enrichr. The last number after the—is dose density. (PDF) [file pone.0238907.s005.pdf]

S5 Table: Chelerythrine chlorid significantly affects the expression of the selected 163 genes as evident in the "LINCS L1000 Chem Pert up/down" category in Enrichr. The last number after the - is dose density.

Term	Overlap	P-value	Adjusted P-value
LINCS L1000 Chem Pert up			
LJP009 MCF7 24H-chelerythrine chloride-10	2.01×10^{-23}	2.22×10^{-19}	
LJP009 HEPG2 24H-chelerythrine chloride-3.33	16/50	6.92×10^{-22}	4.58×10^{-18}
LJP009 PC3 24H-chelerythrine chloride-10	14/149	2.04×10^{-11}	3.11×10^{-9}
LJP009 A375 24H-chelerythrine chloride-1.11	9/55	5.86×10^{-10}	4.84×10^{-8}
LJP009 A375 24H-chelerythrine chloride-3.33	8/57	1.93×10^{-8}	8.87×10^{-7}
LJP009 HT29 24H-chelerythrine chloride-10	9/94	7.48×10^{-8}	2.82×10^{-6}
LJP009 HCC515 24H-chelerythrine chloride-10	8/71	1.13×10^{-7}	4.00×10^{-6}
LJP009 HA1E 24H-chelerythrine chloride-3.33	5/33	6.67×10^{-6}	1.20×10^{-4}
LJP009 HCC515 24H-chelerythrine chloride-0.04	3/28	1.50×10^{-3}	1.08×10^{-2}
LJP009 PC3 24H-chelerythrine chloride-1.11	3/42	4.83×10^{-3}	2.80×10^{-2}
LINCS L1000 Chem Pert down			
LJP009 HEPG2 24H-chelerythrine chloride-10	14/137	6.46×10^{-12}	1.15×10^{-9}
LJP009 HCC515 24H-chelerythrine chloride-10	8/51	7.74×10^{-9}	3.90×10^{-7}
LJP009 A375 24H-chelerythrine chloride-1.11	5/45	3.17×10^{-5}	4.35×10^{-4}
LJP009 HEPG2 24H-chelerythrine chloride-3.33	3/26	1.20×10^{-3}	9.11×10^{-3}
LJP009 A375 24H-chelerythrine chloride-3.33	3/48	7.03×10^{-3}	3.83×10^{-2}
