# Supplementary material for: A new advanced in silico drug discovery method for novel coronavirus (SARS-CoV-2) with tensor decomposition-based unsupervised feature extraction
Source: PLoS One. 2020 Sep 11;15(9):e0238907. doi: 10.1371/journal.pone.0238907 (PMC7485840; doi:10.1371/journal.pone.0238907)
Supplement: S6 Table — Canertinib significantly affects the expression of the selected 163 genes as evident in the “LINCS L1000 Chem Pert up” category in Enrichr. The last number after the—is dose density. (PDF) [file pone.0238907.s006.pdf]

S6 Table: Canertinib significantly affects the expression of the selected 163 genes as evident in the "LINCS L1000 Chem Pert up" category in Enrichr. The last number after the - is dose density.

Term	Overlap	P-value	Adjusted P-value
LINCS L1000 Chem Pert up			
LJP006 HEPG2 24H-canertinib-10	19/96	2.29×10^{-21}	1.27×10^{-17}
LJP006 LNCAP 24H-canertinib-10	14/157	4.14×10^{-11}	5.70×10^{-9}
LJP006 HME1 24H-canertinib-0.12	12/113	1.39×10^{-10}	1.47×10^{-8}
LJP006 HCC515 24H-canertinib-10	11/91	2.02×10^{-10}	1.99×10^{-8}
LJP006 MDAMB231 24H-canertinib-10	11/97	4.08×10^{-10}	3.56×10^{-8}
LJP006 HME1 24H-canertinib-3.33	9/80	1.80×10^{-8}	8.37×10^{-7}
LJP006 MDAMB231 24H-canertinib-3.33	7/39	2.59×10^{-8}	1.14×10^{-6}
LJP006 HME1 24H-canertinib-0.37	10/114	3.22×10^{-8}	1.36×10^{-6}
LJP006 LNCAP 3H-canertinib-10	7/41	3.74×10^{-8}	1.56×10^{-6}
LJP006 HCC515 24H-canertinib-3.33	9/91	$5.63 \times 10^{-}8$	2.21×10^{-6}
LJP006 HCC515 24H-canertinib-1.11	8/77	2.14×10^{-7}	6.75×10^{-6}
LJP006 A375 24H-canertinib-1.11	7/53	2.36×10^{-7}	7.38×10^{-6}
LJP006 MCF10A 24H-canertinib-10	10/153	$5.17 \times 10^{-}7$	1.42×10^{-5}
LJP006 BT20 24H-canertinib-10	9/123	7.57×10^{-7}	1.97×10^{-5}
LJP006 MCF10A 24H-canertinib-0.04	10/163	9.26×10^{-7}	2.33×10^{-5}
LJP006 A375 24H-canertinib-3.33	6/53	4.47×10^{-6}	8.53×10^{-5}
LJP006 HME1 3H-canertinib-1.11	6/56	6.19×10^{-6}	1.13×10^{-4}
LJP006 BT20 24H-canertinib-1.11	7/86	6.57×10^{-6}	1.18×10^{-4}
LJP006 MCF10A 24H-canertinib-0.37	8/128	1.02×10^{-5}	1.70×10^{-4}
LJP006 HME1 3H-canertinib-3.33	6/64	1.36×10^{-5}	2.14×10^{-4}
LJP006 HA1E 24H-canertinib-3.33	5/41	2.00×10^{-5}	2.95×10^{-4}
LJP006 MCF10A 24H-canertinib-3.33	8/146	2.66×10^{-5}	3.79×10^{-4}
LJP006 MCF10A 24H-canertinib-0.12	8/156	4.28×10^{-5}	5.68×10^{-4}
LJP006 HME1 3H-canertinib-0.12	4/26	5.53×10^{-5}	7.03×10^{-4}
LJP006 MDAMB231 3H-canertinib-10	5/51	5.87×10^{-5}	7.36×10^{-4}
LJP006 HCC515 24H-canertinib-0.37	6/84	6.43×10^{-5}	7.95×10^{-4}
LJP006 MCF10A 24H-canertinib-1.11	7/127	8.27×10^{-5}	9.81×10^{-4}
LJP006 HME1 24H-canertinib-1.11	7/128	8.69×10^{-5}	1.02×10^{-3}
LJP006 HME1 3H-canertinib-0.37	5/59	1.19×10^{-4}	1.32×10^{-3}
LJP006 HME1 24H-canertinib-0.04	6/97	1.43×10^{-4}	1.55×10^{-3}
LJP006 PC3 24H-canertinib-10	4/35	1.83×10^{-4}	1.89×10^{-3}
LJP006 MCF7 3H-canertinib-10	4/37	2.28×10^{-4}	2.27×10^{-3}
LJP006 BT20 3H-canertinib-10	4/41	3.40×10^{-4}	3.19×10^{-3}
LJP006 MCF7 24H-canertinib-10	5/75	3.68×10^{-4}	3.41×10^{-3}
LJP006 HCC515 24H-canertinib-0.04	5/76	3.92×10^{-4}	3.59×10^{-3}
LJP006 HEPG2 24H-canertinib-0.04	4/49	6.77×10^{-4}	5.68×10^{-3}
LJP006 HCC515 24H-canertinib-0.12	4/53	9.12×10^{-4}	7.25×10^{-3}
LJP006 SKBR3 24H-canertinib-3.33	5/94	1.04×10^{-3}	8.06×10^{-3}
LJP006 HA1E 24H-canertinib-10	6/152	1.56×10^{-3}	1.12×10^{-2}
LJP006 SKBR3 24H-canertinib-10	5/110	2.08×10^{-3}	1.42×10^{-2}
LJP006 MCF10A 3H-canertinib-3.33	4/70	2.57×10^{-3}	1.70×10^{-2}
LJP006 BT20 24H-canertinib-0.12	4/71	2.70×10^{-3}	1.77×10^{-2}
LJP006 SKBR3 24H-canertinib-0.37	4/78	3.80×10^{-3}	2.31×10^{-2}
LJP006 HS578T 24H-canertinib-10	5/156	9.11×10^{-3}	4.58×10^{-2}
LJP006 BT20 24H-canertinib-3.33	4/102	9.74×10^{-3}	4.81×10^{-2}
