# Supplementary material for: A new advanced in silico drug discovery method for novel coronavirus (SARS-CoV-2) with tensor decomposition-based unsupervised feature extraction
Source: PLoS One. 2020 Sep 11;15(9):e0238907. doi: 10.1371/journal.pone.0238907 (PMC7485840; doi:10.1371/journal.pone.0238907)
Supplement: S7 Table — Canertinib significantly affects the expression of the selected 163 genes as evident in the “LINCS L1000 Chem Pert down” category in Enrichr. The last number after the—is dose density. (PDF) [file pone.0238907.s007.pdf]

S7 Table: Canertinib significantly affects the expression of the selected 163 genes as evident in the "LINCS L1000 Chem Pert down" category in Enrichr. The last number after the - is dose density.

Term	Overlap	P-value	Adjusted P-value
LINCS L1000 Chem Pert down			
LJP006 HME1 24H-canertinib-10	13/89	3.55×10^{-13}	1.12×10^{-10}
LJP006 HME1 3H-canertinib-10	15/139	4.94×10^{-13}	1.46×10^{-10}
LJP006 BT20 24H-canertinib-10	14/128	2.53×10^{-12}	5.44×10^{-10}
LJP006 HS578T 24H-canertinib-10	12/92	1.18×10^{-11}	1.79×10^{-9}
LJP006 BT20 24H-canertinib-3.33	11/81	5.56×10^{-11}	6.53×10^{-9}
LJP006 HA1E 24H-canertinib-10	11/93	2.57×10^{-10}	2.29×10^{-8}
LJP006 SKBR3 24H-canertinib-3.33	10/83	1.43×10^{-9}	9.39×10^{-8}
LJP006 SKBR3 24H-canertinib-0.37	9/65	2.75×10^{-9}	1.62×10^{-7}
LJP006 MCF10A 3H-canertinib-1.11	10/98	7.41×10^{-9}	3.75×10^{-7}
LJP006 HME1 3H-canertinib-1.11	9/82	2.24×10^{-8}	9.31×10^{-7}
LJP006 BT20 24H-canertinib-0.37	8/63	4.34×10^{-8}	1.66×10^{-6}
LJP006 HS578T 3H-canertinib-10	7/54	2.69×10^{-7}	7.75×10^{-6}
LJP006 MCF10A 3H-canertinib-0.37	8/85	4.63×10^{-7}	1.20×10^{-5}
LJP006 MCF10A 24H-canertinib-3.33	11/193	5.44×10^{-7}	1.38×10^{-5}
LJP006 HME1 3H-canertinib-3.33	8/89	6.61×10^{-7}	1.62×10^{-5}
LJP006 BT20 24H-canertinib-0.04	6/46	1.91×10^{-6}	3.98×10^{-5}
LJP006 HS578T 3H-canertinib-0.04	6/49	2.80×10^{-6}	5.46×10^{-5}
LJP006 HCC515 24H-canertinib-10	7/79	3.72×10^{-6}	6.95×10^{-5}
LJP006 BT20 24H-canertinib-1.11	6/52	3.99×10^{-6}	7.42×10^{-5}
LJP006 SKBR3 24H-canertinib-1.11	7/80	4.05×10^{-6}	7.48×10^{-5}
LJP006 SKBR3 24H-canertinib-10	7/85	6.08×10^{-6}	1.06×10^{-4}
LJP006 MCF10A 24H-canertinib-0.12	9/170	1.09×10^{-5}	1.75×10^{-4}
LJP006 MCF10A 3H-canertinib-3.33	7/94	1.19×10^{-5}	1.87×10^{-4}
LJP006 SKBR3 24H-canertinib-0.12	7/94	1.19×10^{-5}	1.87×10^{-4}
LJP006 A375 24H-canertinib-10	7/100	1.78×10^{-5}	2.62×10^{-4}
LJP006 MCF10A 3H-canertinib-0.12	7/100	1.78×10^{-5}	2.62×10^{-4}
LJP006 HA1E 24H-canertinib-3.33	6/71	2.47×10^{-5}	3.51×10^{-4}
LJP006 HCC515 24H-canertinib-3.33	6/72	2.68×10^{-5}	3.77×10^{-4}
LJP006 MCF10A 3H-canertinib-10	5/46	3.54×10^{-5}	4.75×10^{-4}
LJP006 MCF7 3H-canertinib-10	4/28	7.47×10^{-5}	8.87×10^{-4}
LJP006 HME1 3H-canertinib-0.12	5/61	1.39×10^{-4}	1.49×10^{-3}
LJP006 BT20 3H-canertinib-10	4/33	1.45×10^{-4}	1.53×10^{-3}
LJP006 HME1 24H-canertinib-0.12	5/64	1.75×10^{-4}	1.81×10^{-3}
LJP006 HME1 24H-canertinib-0.04	5/72	3.05×10^{-4}	2.90×10^{-3}
LJP006 HME1 24H-canertinib-0.37	5/73	3.25×10^{-4}	3.06×10^{-3}
LJP006 HME1 3H-canertinib-0.37	5/73	3.25×10^{-4}	3.06×10^{-3}
LJP006 MDAMB231 24H-canertinib-10	5/76	3.92×10^{-4}	3.56×10^{-3}
LJP006 SKBR3 3H-canertinib-0.37	3/19	4.68×10^{-4}	4.14×10^{-3}
LJP006 A375 24H-canertinib-3.33	4/45	4.88×10^{-4}	4.29×10^{-3}
LJP006 HME1 24H-canertinib-1.11	5/82	5.56×10^{-4}	4.77×10^{-3}
LJP006 MCF7 24H-canertinib-10	5/83	5.88×10^{-4}	4.98×10^{-3}
LJP006 A375 24H-canertinib-1.11	4/51	7.89×10^{-4}	6.40×10^{-3}
LJP006 MCF10A 3H-canertinib-0.04	4/51	7.89×10^{-4}	6.39×10^{-3}
LJP006 BT20 3H-canertinib-0.12	3/24	9.48×10^{-4}	7.48×10^{-3}
LJP006 SKBR3 24H-canertinib-0.04	5/93	9.86×10^{-4}	7.71×10^{-3}
LJP006 HCC515 24H-canertinib-0.04	3/25	1.07×10^{-3}	8.26×10^{-3}
LJP006 HME1 3H-canertinib-0.04	4/57	$1.20 \times 10^{-}3$	9.10×10^{-3}
LJP006 SKBR3 3H-canertinib-10	3/26	1.20×10^{-3}	9.09×10^{-3}
LJP006 MDAMB231 3H-canertinib-10	4/62	1.64×10^{-3}	1.18×10^{-2}
LJP006 BT20 24H-canertinib-0.12	3/32	2.22×10^{-3}	1.51×10^{-2}
LJP006 MCF7 24H-canertinib-3.33	3/37	3.37×10^{-3}	2.13×10^{-2}
LJP006 HCC515 24H-canertinib-0.37	3/39	3.92×10^{-3}	2.41×10^{-2}
LJP006 MCF10A 24H-canertinib-0.04	6/186	4.25×10^{-3}	2.57×10^{-2}
LJP006 A549 24H-canertinib-10	3/43	5.16×10^{-3}	3.00×10^{-2}
LJP006 MDAMB231 24H-canertinib-3.33	2/17	8.29×10^{-3}	4.36×10^{-2}
