# Supplementary material for: A new advanced in silico drug discovery method for novel coronavirus (SARS-CoV-2) with tensor decomposition-based unsupervised feature extraction
Source: PLoS One. 2020 Sep 11;15(9):e0238907. doi: 10.1371/journal.pone.0238907 (PMC7485840; doi:10.1371/journal.pone.0238907)
Supplement: S8 Table — BX-795 significantly affects the expression of the selected 163 genes as evident in the “LINCS L1000 Chem Pert up/down” category in Enrichr. The last number after the—is dose density. (PDF) [file pone.0238907.s008.pdf]

S8 Table: BX-795 significantly affects the expression of the selected 163 genes as evident in the "LINCS L1000 Chem Pert up/down" category in Enrichr. The last number after the - is dose density.

Term	Overlap	P-value	Adjusted P-value
LINCS L1000 Chem Pert up			
LJP009 PC3 24H-BX-795-10	18/92	3.37×10^{-20}	1.01×10^{-16}
LJP009 HT29 24H-BX-795-10	14/102	1.04×10^{-13}	3.95×10^{-11}
LJP009 HCC515 24H-BX-795-10	9/101	1.40×10^{-7}	4.80×10^{-6}
LJP009 HEPG2 24H-BX-795-10	8/81	3.18×10^{-7}	9.42×10^{-6}
LJP009 MCF7 24H-BX-795-10	9/113	3.68×10^{-7}	1.07×10^{-5}
LJP009 MCF7 24H-BX-795-3.33	5/58	1.09×10^{-4}	1.24×10^{-3}
LJP009 A549 24H-BX-795-10	4/40	3.09×10^{-4}	2.94×10^{-3}
LJP009 HA1E 24H-BX-795-10	3/34	2.64×10^{-3}	1.74×10^{-2}
LJP009 A375 24H-BX-795-1.11	3/51	8.31×10^{-3}	4.26×10^{-2}
LINCS L1000 Chem Pert down			
LJP009 HA1E 24H-BX-795-10	6/43	1.27×10^{-6}	2.84×10^{-5}
LJP009 HA1E 24H-BX-795-3.33	4/37	$2.28 \times 10-4$	2.27×10^{-3}
LJP009 HEPG2 24H-BX-795-3.33	3/36	$3.11 \times 10-3$	2.01×10^{-2}
LJP009 HEPG2 24H-BX-795-10	4/94	7.35×10^{-3}	3.97×10^{-2}
