# Supplementary material for: A new advanced in silico drug discovery method for novel coronavirus (SARS-CoV-2) with tensor decomposition-based unsupervised feature extraction
Source: PLoS One. 2020 Sep 11;15(9):e0238907. doi: 10.1371/journal.pone.0238907 (PMC7485840; doi:10.1371/journal.pone.0238907)
Supplement: S9 Table — Sorafenib significantly affects the expression of the selected 163 genes as evident in the “LINCS L1000 Chem Pert up” category in Enrichr. The last number after the—is dose density. (PDF) [file pone.0238907.s009.pdf]

S9 Table: Sorafenib significantly affects the expression of the selected 163 genes as evident in the "LINCS L1000 Chem Pert up" category in Enrichr. The last number after the - is dose density.

Term	Overlap	P-value	Adjusted P-value
LINCS L1000 Chem Pert up			
LJP006 SKBR3 3H-sorafenib-10	15/56	2.94×10^{-19}	8.10×10^{-16}
LJP006 A549 24H-sorafenib-10	14/146	1.55×10^{-11}	2.50×10^{-9}
LJP006 HEPG2 24H-sorafenib-10	10/74	4.49×10^{-10}	3.87×10^{-8}
LJP006 LNCAP 24H-sorafenib-10	13/165	9.86×10^{-10}	7.44×10^{-8}
LJP006 MCF7 24H-sorafenib-10	8/59	2.55×10^{-8}	1.13×10^{-6}
LJP006 HME1 3H-sorafenib-3.33	7/44	6.24×10^{-8}	2.43×10^{-6}
LJP006 HME1 24H-sorafenib-3.33	9/107	2.30×10^{-7}	7.23×10^{-6}
LJP006 PC3 24H-sorafenib-10	8/78	2.37×10^{-7}	7.36×10^{-6}
LJP006 SKBR3 24H-sorafenib-10	9/111	3.16×10^{-7}	9.36×10^{-6}
LJP006 HT29 24H-sorafenib-10	7/57	3.94×10^{-7}	1.13×10^{-5}
LJP006 HCC515 24H-sorafenib-10	9/117	4.95×10^{-7}	1.38×10^{-5}
LJP006 BT20 24H-sorafenib-10	8/86	5.07×10^{-7}	1.40×10^{-5}
LJP006 HA1E 24H-sorafenib-10	9/119	5.72×10^{-7}	1.55×10^{-5}
LJP006 HME1 24H-sorafenib-10	8/111	3.55×10^{-6}	7.05×10^{-5}
LJP006 BT20 3H-sorafenib-10	4/20	1.86×10^{-5}	2.78×10^{-4}
LJP006 HME1 3H-sorafenib-10	7/114	4.16×10^{-5}	5.55×10^{-4}
LJP006 A375 24H-sorafenib-10	6/84	6.43×10^{-5}	7.96×10^{-4}
LJP006 HME1 24H-sorafenib-1.11	5/78	4.42×10^{-4}	3.96×10^{-3}
LJP006 HEPG2 24H-sorafenib-3.33	4/53	9.12×10^{-4}	7.24×10^{-3}
LINCS L1000 Chem Pert down			
LJP006 HA1E 24H-sorafenib-10	12/92	1.18×10^{-11}	1.80×10^{-9}
LJP006 HME1 24H-sorafenib-10	10/95	5.46×10^{-9}	2.92×10^{-7}
LJP006 HEPG2 24H-sorafenib-10	9/91	5.63×10^{-8}	2.07×10^{-6}
LJP006 HS578T 24H-sorafenib-10	7/63	7.91×10^{-7}	1.89×10^{-5}
LJP006 HEPG2 24H-sorafenib-3.33	6/51	3.55×10^{-6}	6.65×10^{-5}
LJP006 HS578T 3H-sorafenib-10	5/41	2.00×10^{-5}	2.92×10^{-4}
LJP006 HME1 3H-sorafenib-10	6/74	3.13×10^{-5}	4.30×10^{-4}
LJP006 BT20 24H-sorafenib-10	6/79	4.54×10^{-5}	5.84×10^{-4}
LJP006 MDAMB231 3H-sorafenib-10	5/58	1.09×10^{-4}	1.21×10^{-3}
LJP006 SKBR3 24H-sorafenib-10	5/125	3.62×10^{-3}	2.27×10^{-2}
LJP006 HME1 24H-sorafenib-3.33	4/88	5.83×10^{-3}	3.31×10^{-2}
LJP006 MDAMB231 3H-sorafenib-1.11	3/48	7.03×10^{-3}	3.82×10^{-2}
