# Supplementary material for: A new advanced in silico drug discovery method for novel coronavirus (SARS-CoV-2) with tensor decomposition-based unsupervised feature extraction
Source: PLoS One. 2020 Sep 11;15(9):e0238907. doi: 10.1371/journal.pone.0238907 (PMC7485840; doi:10.1371/journal.pone.0238907)
Supplement: S10 Table — QL-X-138 significantly affects the expression of the selected 163 genes as evident in the “LINCS L1000 Chem Pert up” category in Enrichr. The last number after the—is dose density. (PDF) [file pone.0238907.s010.pdf]

S10 Table: QL-X-138 significantly affects the expression of the selected 163 genes as evident in the "LINCS L1000 Chem Pert up" category in Enrichr. The last number after the - is dose density.

Term	Overlap	P-value	Adjusted P-value
LINCS L1000 Chem Pert up			
LJP006 MCF10A 24H-QL-X-138-3.33	11/105	9.66×10^{-10}	7.34×10^{-8}
LJP006 HCC515 24H-QL-X-138-10	13/169	1.32×10^{-9}	9.25×10^{-8}
LJP006 HME1 3H-QL-X-138-3.33	11/111	1.76×10^{-9}	1.17×10^{-7}
LJP006 MDAMB231 24H-QL-X-138-1.11	6/25	4.16×10^{-8}	1.70×10^{-6}
LJP006 HME1 24H-QL-X-138-3.33	11/150	4.21×10^{-8}	1.70×10^{-6}
LJP006 PC3 24H-QL-X-138-3.33	8/68	8.00×10^{-8}	2.98×10^{-6}
LJP006 HME1 24H-QL-X-138-1.11	9/111	3.16×10^{-7}	9.39×10^{-6}
LJP006 HEPG2 24H-QL-X-138-10	9/127	9.91×10^{-7}	2.45×10^{-5}
LJP006 HME1 3H-QL-X-138-0.37	6/48	2.47×10^{-6}	5.22×10^{-5}
LJP006 A375 24H-QL-X-138-1.11	6/50	3.16×10^{-6}	6.37×10^{-5}
LJP006 HEPG2 24H-QL-X-138-1.11	6/51	3.55×10^{-6}	7.02×10^{-5}
LJP006 MCF10A 3H-QL-X-138-1.11	5/30	4.09×10^{-6}	7.86×10^{-5}
LJP006 A375 24H-QL-X-138-10	9/164	8.19×10^{-6}	1.41×10^{-4}
LJP006 HEPG2 24H-QL-X-138-3.33	7/92	1.03×10^{-5}	1.69×10^{-4}
LJP006 MCF7 24H-QL-X-138-3.33	6/65	1.48×10^{-5}	2.30×10^{-4}
LJP006 A549 24H-QL-X-138-10	8/149	3.08×10^{-5}	4.31×10^{-4}
LJP006 HT29 24H-QL-X-138-10	5/46	3.54×10^{-5}	4.83×10^{-4}
LJP006 HCC515 24H-QL-X-138-3.33	5/53	7.07×10^{-5}	8.57×10^{-4}
LJP006 HS578T 24H-QL-X-138-0.37	5/54	7.75×10^{-5}	9.27×10^{-4}
LJP006 HME1 3H-QL-X-138-10	6/90	9.46×10^{-5}	1.10×10^{-3}
LJP006 SKBR3 24H-QL-X-138-0.37	5/62	1.50×10^{-4}	1.61×10^{-3}
LJP006 HME1 24H-QL-X-138-10	8/189	1.64×10^{-4}	1.72×10^{-3}
LJP006 LNCAP 24H-QL-X-138-3.33	5/65	1.88×10^{-4}	1.93×10^{-3}
LJP006 MCF7 24H-QL-X-138-0.12	4/42	3.74×10^{-4}	3.44×10^{-3}
LJP006 MCF10A 3H-QL-X-138-0.37	4/46	5.31×10^{-4}	4.63×10^{-3}
LJP006 SKBR3 24H-QL-X-138-1.11	5/83	5.88×10^{-4}	5.02×10^{-3}
LJP006 PC3 24H-QL-X-138-10	7/186	8.46×10^{-4}	6.83×10^{-3}
LJP006 MDAMB231 3H-QL-X-138-10	4/54	9.79×10^{-4}	7.68×10^{-3}
LJP006 PC3 24H-QL-X-138-1.11	4/55	1.05×10^{-3}	8.14×10^{-3}
LJP006 MDAMB231 24H-QL-X-138-3.33	3/25	1.07×10^{-3}	8.26×10^{-3}
LJP006 HS578T 24H-QL-X-138-0.12	4/56	1.12×10^{-3}	8.58×10^{-3}
LJP006 A375 24H-QL-X-138-3.33	5/97	1.19×10^{-3}	9.04×10^{-3}
LJP006 HS578T 24H-QL-X-138-1.11	4/58	1.28×10^{-3}	9.54×10^{-3}
LJP006 MCF7 24H-QL-X-138-1.11	4/59	1.37×10^{-3}	1.00×10^{-2}
LJP006 MCF7 24H-QL-X-138-10	5/101	1.43×10^{-3}	1.04×10^{-2}
LJP006 SKBR3 24H-QL-X-138-10	6/173	2.98×10^{-3}	1.91×10^{-2}
LJP006 BT20 24H-QL-X-138-10	6/177	3.34×10^{-3}	2.10×10^{-2}
LJP006 PC3 24H-QL-X-138-0.37	3/37	3.37×10^{-3}	2.11×10^{-2}
LJP006 HS578T 3H-QL-X-138-10	4/77	3.63×10^{-3}	2.24×10^{-2}
LJP006 MCF10A 24H-QL-X-138-10	6/188	4.48×10^{-3}	2.65×10^{-2}
LJP006 HS578T 3H-QL-X-138-3.33	3/42	4.83×10^{-3}	2.79×10^{-2}
LJP006 LNCAP 24H-QL-X-138-1.11	3/44	5.51×10^{-3}	3.09×10^{-2}
LJP006 BT20 24H-QL-X-138-3.33	4/91	6.56×10^{-3}	3.54×10^{-2}
LJP006 MCF7 3H-QL-X-138-10	3/51	8.31×10^{-3}	4.25×10^{-2}
