# Supplementary material for: A new advanced in silico drug discovery method for novel coronavirus (SARS-CoV-2) with tensor decomposition-based unsupervised feature extraction
Source: PLoS One. 2020 Sep 11;15(9):e0238907. doi: 10.1371/journal.pone.0238907 (PMC7485840; doi:10.1371/journal.pone.0238907)
Supplement: S11 Table — QL-X-138 significantly affects the expression of the selected 163 genes as evident in the “LINCS L1000 Chem Pert down” category in Enrichr. The last number after the—is dose density. (PDF) [file pone.0238907.s011.pdf]

S11 Table: QL-X-138 significantly affects the expression of the selected 163 genes as evident in the "LINCS L1000 Chem Pert down" category in Enrichr. The last number after the - is dose density.

Term	Overlap	P-value	Adjusted P-value
LINCS L1000 Chem Pert down			
LJP006 HA1E 24H-QL-X-138-10	33/219	1.76×10^{-32}	5.84×10^{-28}
LJP006 HA1E 24H-QL-X-138-3.33	21/201	1.62×10^{-17}	4.47×10^{-14}
LJP006 HS578T 24H-QL-X-138-10	22/230	1.77×10^{-17}	4.51×10^{-14}
LJP006 BT20 24H-QL-X-138-10	19/157	3.74×10^{-17}	7.29×10^{-14}
LJP006 HS578T 24H-QL-X-138-3.33	13/97	1.11×10^{-12}	2.86×10^{-10}
LJP006 HA1E 24H-QL-X-138-0.37	11/60	1.82×10^{-12}	4.22×10^{-10}
LJP006 HA1E 24H-QL-X-138-1.11	13/101	1.89×10^{-12}	4.27×10^{-10}
LJP006 SKBR3 24H-QL-X-138-10	15/153	2.04×10^{-12}	4.53×10^{-10}
LJP006 HME1 24H-QL-X-138-10	14/149	2.04×10^{-11}	2.81×10^{-9}
LJP006 BT20 24H-QL-X-138-3.33	10/71	2.95×10^{-10}	2.58×10^{-8}
LJP006 HCC515 24H-QL-X-138-10	11/124	5.74×10^{-9}	3.04×10^{-7}
LJP006 HEPG2 24H-QL-X-138-10	12/176	2.27×10^{-8}	9.40×10^{-7}
LJP006 BT20 24H-QL-X-138-1.11	9/98	1.08×10^{-7}	3.57×10^{-6}
LJP006 MDAMB231 24H-QL-X-138-3.33	6/29	1.09×10^{-7}	3.60×10^{-6}
LJP006 PC3 24H-QL-X-138-10	10/148	3.80×10^{-7}	1.02×10^{-5}
LJP006 HS578T 3H-QL-X-138-10	7/68	1.34×10^{-6}	2.95×10^{-5}
LJP006 A549 24H-QL-X-138-10	9/136	1.76×10^{-6}	3.71×10^{-5}
LJP006 LNCAP 24H-QL-X-138-1.11	8/105	2.34×10^{-6}	4.73×10^{-5}
LJP006 HS578T 24H-QL-X-138-0.37	6/52	3.99×10^{-6}	7.40×10^{-5}
LJP006 HME1 24H-QL-X-138-3.33	8/117	5.26×10^{-6}	9.38×10^{-5}
LJP006 A375 24H-QL-X-138-3.33	8/118	5.61×10^{-6}	9.92×10^{-5}
LJP006 HME1 3H-QL-X-138-3.33	6/56	6.19×10^{-6}	1.07×10^{-4}
LJP006 LNCAP 24H-QL-X-138-3.33	8/121	6.76×10^{-6}	1.16×10^{-4}
LJP006 MCF7 24H-QL-X-138-1.11	5/35	9.01×10^{-6}	1.48×10^{-4}
LJP006 HME1 24H-QL-X-138-1.11	7/92	1.03×10^{-5}	1.66×10^{-4}
LJP006 SKBR3 24H-QL-X-138-0.12	5/38	1.37×10^{-5}	2.10×10^{-4}
LJP006 HA1E 24H-QL-X-138-0.12	5/40	1.77×10^{-5}	2.63×10^{-4}
LJP006 HT29 24H-QL-X-138-10	5/40	1.77×10^{-5}	2.63×10^{-4}
LJP006 MCF7 24H-QL-X-138-0.12	6/77	3.93×10^{-5}	5.20×10^{-4}
LJP006 SKBR3 24H-QL-X-138-1.11	6/81	5.24×10^{-5}	6.60×10^{-4}
LJP006 BT20 3H-QL-X-138-3.33	4/29	8.61×10^{-5}	9.96×10^{-4}
LJP006 A375 24H-QL-X-138-10	8/176	1.00×10^{-4}	1.13×10^{-3}
LJP006 MCF10A 24H-QL-X-138-10	7/133	1.11×10^{-4}	1.22×10^{-3}
LJP006 BT20 3H-QL-X-138-10	4/35	1.83×10^{-4}	1.87×10^{-3}
LJP006 HS578T 24H-QL-X-138-1.11	5/68	2.33×10^{-4}	2.30×10^{-3}
LJP006 MCF7 24H-QL-X-138-10	5/82	5.56×10^{-4}	4.77×10^{-3}
LJP006 SKBR3 3H-QL-X-138-1.11	3/22	7.30×10^{-4}	6.00×10^{-3}
LJP006 PC3 24H-QL-X-138-0.12	3/23	8.35×10^{-4}	6.72×10^{-3}
LJP006 SKBR3 3H-QL-X-138-0.37	3/25	1.07×10^{-3}	8.25×10^{-3}
LJP006 MCF7 24H-QL-X-138-0.37	3/28	1.50×10^{-3}	1.09×10^{-2}
LJP006 HME1 3H-QL-X-138-0.37	3/32	2.22×10^{-3}	1.51×10^{-2}
LJP006 SKBR3 24H-QL-X-138-0.37	4/68	2.31×10^{-3}	1.56×10^{-2}
LJP006 A375 24H-QL-X-138-1.11	3/34	2.64×10^{-3}	1.75×10^{-2}
LJP006 BT20 24H-QL-X-138-0.37	3/36	3.11×10^{-3}	2.01×10^{-2}
LJP006 PC3 24H-QL-X-138-0.37	3/39	3.92×10^{-3}	2.40×10^{-2}
LJP006 MCF7 24H-QL-X-138-3.33	3/42	4.83×10^{-3}	2.84×10^{-2}
LJP006 SKBR3 3H-QL-X-138-3.33	3/45	5.87×10^{-3}	3.31×10^{-2}
LJP006 BT20 3H-QL-X-138-0.37	2/15	6.47×10^{-3}	3.57×10^{-2}
LJP006 SKBR3 24H-QL-X-138-3.33	5/144	6.56×10^{-3}	3.62×10^{-2}
LJP006 PC3 24H-QL-X-138-3.33	3/48	7.03×10^{-3}	3.82×10^{-2}
