# Supplementary material for: A new advanced in silico drug discovery method for novel coronavirus (SARS-CoV-2) with tensor decomposition-based unsupervised feature extraction
Source: PLoS One. 2020 Sep 11;15(9):e0238907. doi: 10.1371/journal.pone.0238907 (PMC7485840; doi:10.1371/journal.pone.0238907)
Supplement: S12 Table — Radicicol significantly affects the expression of the selected 163 genes due to “LINCS L1000 Chem Pert up” category in Enrichr. The last number after the—is dose density. (PDF) [file pone.0238907.s012.pdf]

S12 Table: Radicicol significantly affects the expression of the selected 163 genes due to “LINCS L1000 Chem Pert up” category in Enrichr. The last number after the - is dose density.

Term	Overlap	P-value	Adjusted P-value
LINCS L1000 Chem Pert up			
LJP006 MCF7 24H-radicicol-10	17/149	5.02×10^{-15}	3.39×10^{-12}
LJP006 SKBR3 3H-radicicol-10	13/74	2.94×10^{-14}	1.33×10^{-11}
LJP006 MCF7 24H-radicicol-3.33	15/123	7.94×10^{-14}	3.09×10^{-11}
LJP005 HS578T 3H-radicicol-10	10/50	7.59×10^{-12}	1.43×10^{-9}
LJP005 MCF10A 24H-radicicol-3.33	15/184	$2.95 \times 10^{-1}1$	4.18×10^{-9}
LJP005 MCF7 24H-radicicol-1.11	12/109	9.04×10^{-11}	1.05×10^{-8}
LJP005 HA1E 24H-radicicol-3.33	11/87	1.23×10^{-10}	1.34×10^{-8}
LJP006 MCF7 24H-radicicol-1.11	11/87	1.23×10^{-10}	1.33×10^{-8}
LJP006 HME1 3H-radicicol-3.33	11/92	2.28×10^{-10}	2.20×10^{-8}
LJP006 HME1 24H-radicicol-3.33	13/147	2.34×10^{-10}	2.24×10^{-8}
LJP006 HEPG2 24H-radicicol-10	14/179	2.40×10^{-10}	2.29×10^{-8}
LJP006 BT20 24H-radicicol-10	14/180	2.59×10^{-10}	2.43×10^{-8}
LJP005 BT20 3H-radicicol-10	10/71	2.95×10^{-10}	2.74×10^{-8}
LJP005 SKBR3 3H-radicicol-10	10/71	2.95×10^{-10}	2.74×10^{-8}
LJP005 MCF10A 3H-radicicol-3.33	11/97	4.08×10^{-10}	3.56×10^{-8}
LJP005 MCF7 24H-radicicol-10	13/159	6.23×10^{-10}	5.11×10^{-8}
LJP005 SKBR3 24H-radicicol-10	13/163	8.48×10^{-10}	6.58×10^{-8}
LJP005 MCF7 24H-radicicol-3.33	12/133	$9.36 \times 10^{-1}0$	7.16×10^{-8}
LJP005 SKBR3 3H-radicicol-0.04	7/26	1.21×10^{-9}	8.61×10^{-8}
LJP005 HEPG2 24H-radicicol-1.11	11/112	1.94×10^{-9}	1.27×10^{-7}
LJP005 MCF7 3H-radicicol-0.12	9/66	3.16×10^{-9}	1.90×10^{-7}
LJP005 MCF10A 24H-radicicol-10	14/219	3.37×10^{-9}	2.00×10^{-7}
LJP005 MCF10A 3H-radicicol-10	10/97	6.70×10^{-9}	3.67×10^{-7}
LJP005 MDAMB231 24H-radicicol-3.33	9/73	7.90×10^{-9}	4.23×10^{-7}
LJP006 HCC515 24H-radicicol-10	10/99	8.19×10^{-9}	4.35×10^{-7}
LJP006 HA1E 24H-radicicol-3.33	10/100	9.03×10^{-9}	4.75×10^{-7}
LJP006 SKBR3 24H-radicicol-10	13/203	1.23×10^{-8}	6.08×10^{-7}
LJP005 A375 24H-radicicol-3.33	10/104	1.32×10^{-8}	6.46×10^{-7}
LJP005 SKBR3 3H-radicicol-1.11	7/37	1.76×10^{-8}	8.22×10^{-7}
LJP006 HME1 24H-radicicol-0.37	11/145	2.97×10^{-8}	1.27×10^{-6}
LJP006 LNCAP 3H-radicicol-0.04	7/40	3.12×10^{-8}	1.33×10^{-6}
LJP006 MCF10A 24H-radicicol-0.37	12/186	4.19×10^{-8}	1.70×10^{-6}
LJP005 BT20 3H-radicicol-1.11	8/65	5.57×10^{-8}	2.20×10^{-6}
LJP006 BT20 3H-radicicol-10	7/45	7.34×10^{-8}	2.78×10^{-6}
LJP006 LNCAP 24H-radicicol-10	10/131	1.21×10^{-7}	4.24×10^{-6}
LJP005 PC3 24H-radicicol-10	8/72	1.26×10^{-7}	4.38×10^{-6}
LJP005 HS578T 24H-radicicol-1.11	7/49	1.35×10^{-7}	4.66×10^{-6}
LJP005 HCC515 24H-radicicol-10	9/101	1.40×10^{-7}	4.79×10^{-6}
LJP005 HEPG2 24H-radicicol-3.33	10/133	1.40×10^{-7}	4.78×10^{-6}
LJP006 PC3 24H-radicicol-10	10/139	2.12×10^{-7}	6.73×10^{-6}
LJP005 HA1E 24H-radicicol-10	11/177	2.29×10^{-7}	7.18×10^{-6}
LJP006 MCF10A 24H-radicicol-3.33	11/178	2.42×10^{-7}	7.51×10^{-6}
LJP005 BT20 3H-radicicol-0.04	5/18	2.66×10^{-7}	8.17×10^{-6}
LJP006 MCF10A 3H-radicicol-0.37	7/57	3.94×10^{-7}	1.13×10^{-5}
LJP006 MCF7 3H-radicicol-3.33	6/37	5.04×10^{-7}	1.39×10^{-5}
LJP006 SKBR3 3H-radicicol-3.33	8/87	5.54×10^{-7}	1.52×10^{-5}
LJP006 HEPG2 24H-radicicol-3.33	8/90	7.20×10^{-7}	1.89×10^{-5}
LJP006 MCF10A 24H-radicicol-0.12	9/123	7.57×10^{-7}	1.97×10^{-5}
LJP005 MCF10A 24H-radicicol-1.11	11/202	8.54×10^{-7}	2.17×10^{-5}
LJP005 HCC515 24H-radicicol-3.33	7/64	8.83×10^{-7}	2.23×10^{-5}
LJP006 HCC515 24H-radicicol-1.11	8/93	9.27×10^{-7}	2.33×10^{-5}
LJP005 MCF10A 3H-radicicol-0.04	6/42	1.10×10^{-6}	2.66×10^{-5}
LJP006 LNCAP 24H-radicicol-3.33	8/96	1.18×10^{-6}	2.84×10^{-5}

LJP005 SKBR3 3H-radicicol-3.33	7/67	1.21×10^{-6}	2.89×10^{-5}
LJP005 PC3 24H-radicicol-0.37	6/43	1.27×10^{-6}	3.02×10^{-5}
LJP006 MCF10A 24H-radicicol-10	11/212	1.37×10^{-6}	3.21×10^{-5}
LJP005 SKBR3 24H-radicicol-3.33	9/133	1.46×10^{-6}	3.40×10^{-5}
LJP005 HEPG2 24H-radicicol-10	10/177	1.96×10^{-6}	4.30×10^{-5}
LJP006 A375 24H-radicicol-3.33	7/74	2.39×10^{-6}	5.08×10^{-5}
LJP006 HS578T 3H-radicicol-1.11	6/49	2.80×10^{-6}	5.81×10^{-5}
LJP006 SKBR3 24H-radicicol-3.33	8/109	3.10×10^{-6}	6.32×10^{-5}
LJP005 BT20 24H-radicicol-1.11	6/50	3.16×10^{-6}	6.38×10^{-5}
LJP005 MCF10A 3H-radicicol-1.11	6/50	3.16×10^{-6}	6.38×10^{-5}
LJP006 MCF10A 3H-radicicol-10	8/110	3.32×10^{-6}	6.65×10^{-5}
LJP005 MCF10A 24H-radicicol-0.12	8/111	3.55×10^{-6}	7.06×10^{-5}
LJP006 MCF7 24H-radicicol-0.37	6/51	3.55×10^{-6}	7.02×10^{-5}
LJP006 HA1E 24H-radicicol-0.37	7/79	3.72×10^{-6}	7.30×10^{-5}
LJP006 PC3 24H-radicicol-0.37	5/31	4.84×10^{-6}	9.15×10^{-5}
LJP006 HME1 3H-radicicol-10	8/119	5.97×10^{-6}	1.09×10^{-4}
LJP006 HME1 3H-radicicol-1.11	7/86	6.57×10^{-6}	1.18×10^{-4}
LJP006 MCF10A 24H-radicicol-1.11	10/204	6.95×10^{-6}	1.24×10^{-4}
LJP006 HS578T 24H-radicicol-3.33	8/123	7.63×10^{-6}	1.33×10^{-4}
LJP005 MDAMB231 3H-radicicol-0.12	5/35	9.01×10^{-6}	1.53×10^{-4}
LJP006 MCF10A 3H-radicicol-0.12	6/60	9.30×10^{-6}	1.57×10^{-4}
LJP005 HA1E 24H-radicicol-0.37	6/64	1.36×10^{-5}	2.15×10^{-4}
LJP006 MDAMB231 24H-radicicol-10	7/96	1.36×10^{-5}	2.14×10^{-4}
LJP006 HME1 3H-radicicol-0.04	4/19	1.50×10^{-5}	2.32×10^{-4}
LJP006 MCF7 3H-radicicol-0.12	4/19	1.50×10^{-5}	2.32×10^{-4}
LJP005 MDAMB231 24H-radicicol-10	5/40	1.77×10^{-5}	2.67×10^{-4}
LJP006 HME1 3H-radicicol-0.12	5/41	2.00×10^{-5}	2.95×10^{-4}
LJP005 SKBR3 24H-radicicol-0.12	6/70	2.28×10^{-5}	3.31×10^{-4}
LJP005 MDAMB231 3H-radicicol-1.11	5/43	2.53×10^{-5}	3.63×10^{-4}
LJP005 HT29 24H-radicicol-10	6/72	2.68×10^{-5}	3.80×10^{-4}
LJP006 HME1 24H-radicicol-0.12	7/111	3.50×10^{-5}	4.80×10^{-4}
LJP006 LNCAP 3H-radicicol-3.33	5/46	3.54×10^{-5}	4.83×10^{-4}
LJP006 SKBR3 3H-radicicol-0.04	4/24	3.98×10^{-5}	5.32×10^{-4}
LJP006 MCF7 3H-radicicol-10	5/48	4.36×10^{-5}	5.77×10^{-4}
LJP006 HCC515 24H-radicicol-3.33	6/79	4.54×10^{-5}	5.97×10^{-4}
LJP006 MCF10A 3H-radicicol-3.33	6/79	4.54×10^{-5}	5.97×10^{-4}
LJP005 BT20 24H-radicicol-10	9/204	4.63×10^{-5}	6.06×10^{-4}
LJP006 PC3 24H-radicicol-0.12	4/25	4.71×10^{-5}	6.14×10^{-4}
LJP005 SKBR3 24H-radicicol-0.37	7/119	5.47×10^{-5}	6.99×10^{-4}
LJP005 MCF10A 24H-radicicol-0.37	8/162	5.59×10^{-5}	7.11×10^{-4}
LJP005 SKBR3 3H-radicicol-0.37	5/53	7.07×10^{-5}	8.58×10^{-4}
LJP006 MDAMB231 3H-radicicol-1.11	4/29	8.61×10^{-5}	1.01×10^{-3}
LJP006 SKBR3 24H-radicicol-0.37	6/90	9.46×10^{-5}	1.10×10^{-3}
LJP005 BT20 24H-radicicol-3.33	6/92	1.07×10^{-4}	1.21×10^{-3}
LJP006 LNCAP 3H-radicicol-0.37	4/32	1.28×10^{-4}	1.40×10^{-3}
LJP006 HT29 24H-radicicol-10	5/60	1.29×10^{-4}	1.41×10^{-3}
LJP006 A549 24H-radicicol-0.37	4/33	1.45×10^{-4}	1.56×10^{-3}
LJP006 HME1 3H-radicicol-0.37	5/63	1.62×10^{-4}	1.71×10^{-3}
LJP006 SKBR3 24H-radicicol-1.11	6/100	1.69×10^{-4}	1.77×10^{-3}
LJP005 HCC515 24H-radicicol-1.11	5/64	1.75×10^{-4}	1.82×10^{-3}
LJP006 HA1E 24H-radicicol-10	9/248	2.05×10^{-4}	2.06×10^{-3}
LJP006 LNCAP 3H-radicicol-0.12	4/37	2.28×10^{-4}	2.27×10^{-3}
LJP006 MDAMB231 3H-radicicol-10	4/39	2.80×10^{-4}	2.71×10^{-3}
LJP006 BT20 24H-radicicol-3.33	7/155	2.84×10^{-4}	2.74×10^{-3}
LJP006 MDAMB231 24H-radicicol-3.33	5/72	3.05×10^{-4}	2.90×10^{-3}
LJP005 MCF10A 3H-radicicol-0.37	4/40	3.09×10^{-4}	2.94×10^{-3}

S12 Table: (Continued)

LJP005 HS578T 3H-radicicol-1.11	4/41	3.40×10^{-4}	3.20×10^{-3}
LJP005 SKBR3 3H-radicicol-0.12	4/41	3.40×10^{-4}	3.19×10^{-3}
LJP006 BT20 24H-radicicol-0.12	4/42	3.74×10^{-4}	3.44×10^{-3}
LJP006 BT20 24H-radicicol-1.11	6/119	4.34×10^{-4}	3.91×10^{-3}
LJP006 MCF7 24H-radicicol-0.12	4/44	4.48×10^{-4}	4.00×10^{-3}
LJP006 PC3 24H-radicicol-3.33	5/80	4.97×10^{-4}	4.36×10^{-3}
LJP006 PC3 24H-radicicol-1.11	4/47	5.77×10^{-4}	4.95×10^{-3}
LJP005 A375 24H-radicicol-1.11	5/84	6.21×10^{-4}	5.28×10^{-3}
LJP006 HS578T 24H-radicicol-10	5/87	7.29×10^{-4}	6.05×10^{-3}
LJP005 BT20 3H-radicicol-0.12	3/22	7.30×10^{-4}	6.05×10^{-3}
LJP006 A375 24H-radicicol-10	6/132	7.51×10^{-4}	6.16×10^{-3}
LJP005 MCF7 3H-radicicol-0.37	4/52	8.49×10^{-4}	6.84×10^{-3}
LJP005 HA1E 24H-radicicol-0.12	4/53	9.12×10^{-4}	7.26×10^{-3}
LJP006 SKBR3 3H-radicicol-0.37	3/24	9.48×10^{-4}	7.49×10^{-3}
LJP005 BT20 3H-radicicol-3.33	4/54	9.79×10^{-4}	7.71×10^{-3}
LJP005 MDAMB231 3H-radicicol-10	4/54	9.79×10^{-4}	7.69×10^{-3}
LJP005 MDAMB231 24H-radicicol-0.37	3/25	1.07×10^{-3}	8.28×10^{-3}
LJP005 HS578T 24H-radicicol-10	5/96	1.14×10^{-3}	8.67×10^{-3}
LJP006 MDAMB231 3H-radicicol-0.04	3/26	1.20×10^{-3}	9.07×10^{-3}
LJP005 MCF7 3H-radicicol-10	4/61	1.55×10^{-3}	1.11×10^{-2}
LJP005 MDAMB231 3H-radicicol-3.33	3/29	1.66×10^{-3}	1.18×10^{-2}
LJP006 HME1 24H-radicicol-1.11	5/108	1.92×10^{-3}	1.33×10^{-2}
LJP005 HEPG2 24H-radicicol-0.37	4/67	2.19×10^{-3}	1.49×10^{-2}
LJP005 HS578T 3H-radicicol-0.12	3/32	2.22×10^{-3}	1.50×10^{-2}
LJP006 MDAMB231 3H-radicicol-0.37	3/32	2.22×10^{-3}	1.50×10^{-2}
LJP006 MDAMB231 24H-radicicol-1.11	4/68	2.31×10^{-3}	1.55×10^{-2}
LJP005 MDAMB231 24H-radicicol-1.11	3/34	2.64×10^{-3}	1.74×10^{-2}
LJP006 HS578T 3H-radicicol-0.12	3/34	2.64×10^{-3}	1.73×10^{-2}
LJP005 BT20 24H-radicicol-0.12	3/35	2.87×10^{-3}	1.86×10^{-2}
LJP005 HS578T 3H-radicicol-0.04	3/39	3.92×10^{-3}	2.38×10^{-2}
LJP006 HS578T 3H-radicicol-3.33	3/41	4.51×10^{-3}	2.66×10^{-2}
LJP006 LNCAP 3H-radicicol-1.11	3/41	4.51×10^{-3}	2.65×10^{-2}
LJP006 LNCAP 24H-radicicol-1.11	4/84	4.95×10^{-3}	2.84×10^{-2}
LJP005 HS578T 3H-radicicol-3.33	3/44	5.51×10^{-3}	3.09×10^{-2}
LJP006 HA1E 24H-radicicol-1.11	4/88	5.83×10^{-3}	3.24×10^{-2}
LJP006 A375 24H-radicicol-0.37	4/89	6.07×10^{-3}	3.33×10^{-2}
LJP005 HA1E 24H-radicicol-1.11	4/94	7.35×10^{-3}	3.89×10^{-2}
LJP005 HS578T 24H-radicicol-0.37	3/49	7.44×10^{-3}	3.92×10^{-2}
LJP005 MCF7 24H-radicicol-0.37	3/49	7.44×10^{-3}	3.92×10^{-2}
LJP006 BT20 24H-radicicol-0.37	3/50	7.87×10^{-3}	4.10×10^{-2}
LJP005 HCC515 24H-radicicol-0.37	3/53	9.24×10^{-3}	4.63×10^{-2}
LJP006 LNCAP 3H-radicicol-10	3/53	9.24×10^{-3}	4.62×10^{-2}
LJP006 HS578T 3H-radicicol-10	3/54	9.73×10^{-3}	4.82×10^{-2}
