# Supplementary material for: A new advanced in silico drug discovery method for novel coronavirus (SARS-CoV-2) with tensor decomposition-based unsupervised feature extraction
Source: PLoS One. 2020 Sep 11;15(9):e0238907. doi: 10.1371/journal.pone.0238907 (PMC7485840; doi:10.1371/journal.pone.0238907)
Supplement: S13 Table — Radicicol significantly affects the expression of the selected 163 genes due to “LINCS L1000 Chem Pert up” category in Enrichr. The last number after the—is dose density. (PDF) [file pone.0238907.s013.pdf]

S13 Table: Radicicol significantly affects the expression of the selected 163 genes as evident in the "LINCS L1000 Chem Pert down" category in Enrichr. The last number after the - is dose density.

Term	Overlap	P-value	Adjusted P-value
LINCS L1000 Chem Pert down			
LJP006 HA1E 24H-radicicol-3.33	22/160	6.04×10^{-21}	1.00×10^{-16}
LJP005 HS578T 24H-radicicol-10	16/109	5.56×10^{-16}	6.14×10^{-13}
LJP005 HS578T 24H-radicicol-3.33	15/98	2.46×10^{-15}	2.09×10^{-12}
LJP005 SKBR3 24H-radicicol-0.12	11/56	8.13×10^{-13}	2.23×10^{-10}
LJP005 SKBR3 24H-radicicol-0.37	13/106	3.57×10^{-12}	7.25×10^{-10}
LJP005 HA1E 24H-radicicol-3.33	14/132	3.87×10^{-12}	7.68×10^{-10}
LJP006 A375 24H-radicicol-0.37	14/133	4.30×10^{-12}	8.23×10^{-10}
LJP006 MDAMB231 24H-radicicol-10	13/108	4.55×10^{-12}	8.52×10^{-10}
LJP006 MCF10A 3H-radicicol-1.11	12/95	1.74×10^{-11}	2.48×10^{-9}
LJP006 HS578T 24H-radicicol-10	13/128	4.07×10^{-11}	5.13×10^{-9}
LJP006 MCF10A 24H-radicicol-10	15/190	4.67×10^{-11}	5.75×10^{-9}
LJP005 MDAMB231 24H-radicicol-3.33	12/107	7.25×10^{-11}	8.14×10^{-9}
LJP006 SKBR3 24H-radicicol-0.37	12/108	8.10×10^{-11}	8.71×10^{-9}
LJP006 HS578T 24H-radicicol-3.33	12/115	1.71×10^{-10}	1.61×10^{-8}
LJP006 HCC515 24H-radicicol-10	13/158	5.76×10^{-10}	4.45×10^{-8}
LJP005 PC3 24H-radicicol-1.11	9/58	9.61×10^{-10}	6.80×10^{-8}
LJP005 A375 24H-radicicol-1.11	12/134	1.02×10^{-9}	7.15×10^{-8}
LJP005 A549 24H-radicicol-10	12/142	1.99×10^{-9}	1.25×10^{-7}
LJP005 MCF10A 3H-radicicol-3.33	10/90	3.20×10^{-9}	1.85×10^{-7}
LJP006 MCF10A 3H-radicicol-0.12	9/71	6.14×10^{-9}	3.19×10^{-7}
LJP006 BT20 24H-radicicol-0.37	9/75	1.01×10^{-8}	4.86×10^{-7}
LJP005 SKBR3 24H-radicicol-1.11	10/103	1.21×10^{-8}	5.66×10^{-7}
LJP006 SKBR3 24H-radicicol-1.11	10/106	1.59×10^{-8}	7.07×10^{-7}
LJP006 SKBR3 24H-radicicol-0.12	8/56	1.67×10^{-8}	7.35×10^{-7}
LJP005 MCF10A 3H-radicicol-0.37	9/80	1.80×10^{-8}	7.82×10^{-7}
LJP006 HME1 24H-radicicol-3.33	12/177	2.42×10^{-8}	9.96×10^{-7}
LJP006 BT20 24H-radicicol-1.11	9/86	3.42×10^{-8}	1.34×10^{-6}
LJP006 SKBR3 24H-radicicol-3.33	10/122	6.17×10^{-8}	2.24×10^{-6}
LJP006 HS578T 3H-radicicol-0.37	7/44	6.24×10^{-8}	2.25×10^{-6}
LJP006 MCF10A 3H-radicicol-3.33	9/93	6.81×10^{-8}	2.42×10^{-6}
LJP006 HA1E 24H-radicicol-1.11	11/158	7.20×10^{-8}	2.53×10^{-6}
LJP005 PC3 24H-radicicol-0.37	7/46	8.60×10^{-8}	2.95×10^{-6}
LJP006 A549 24H-radicicol-10	10/131	1.21×10^{-7}	3.95×10^{-6}
LJP006 HME1 24H-radicicol-1.11	10/131	1.21×10^{-7}	3.94×10^{-6}
LJP005 MCF10A 24H-radicicol-10	12/212	1.76×10^{-7}	5.44×10^{-6}
LJP006 HS578T 24H-radicicol-1.11	9/106	2.12×10^{-7}	6.37×10^{-6}
LJP005 BT20 24H-radicicol-0.37	7/55	3.07×10^{-7}	8.59×10^{-6}
LJP006 MDAMB231 24H-radicicol-3.33	8/82	3.50×10^{-7}	9.56×10^{-6}
LJP005 HA1E 24H-radicicol-1.11	10/147	3.57×10^{-7}	9.68×10^{-6}
LJP006 HME1 3H-radicicol-0.37	7/57	3.94×10^{-7}	1.06×10^{-5}
LJP006 HA1E 24H-radicicol-10	9/114	3.97×10^{-7}	1.06×10^{-5}
LJP005 HCC515 24H-radicicol-10	10/154	5.49×10^{-7}	1.39×10^{-5}
LJP006 HS578T 3H-radicicol-3.33	6/44	1.46×10^{-6}	3.18×10^{-5}
LJP005 A375 24H-radicicol-0.37	8/99	1.50×10^{-6}	3.24×10^{-5}
LJP005 HA1E 24H-radicicol-0.37	7/71	1.80×10^{-6}	3.79×10^{-5}
LJP005 MCF10A 3H-radicicol-1.11	7/77	3.13×10^{-6}	6.01×10^{-5}
LJP006 BT20 24H-radicicol-3.33	8/110	3.32×10^{-6}	6.32×10^{-5}
LJP006 A375 24H-radicicol-0.12	7/78	3.41×10^{-6}	6.47×10^{-5}
LJP005 HS578T 3H-radicicol-3.33	5/32	5.70×10^{-6}	1.00×10^{-4}
LJP005 MCF10A 3H-radicicol-10	7/92	1.03×10^{-5}	1.66×10^{-4}
LJP006 MCF7 3H-radicicol-10	5/40	1.77×10^{-5}	2.63×10^{-4}
LJP006 MCF7 24H-radicicol-1.11	7/103	2.16×10^{-5}	3.12×10^{-4}
LJP006 MCF10A 3H-radicicol-0.37	6/70	2.28×10^{-5}	3.26×10^{-4}

S13 Table: (Continued)

LJP005 SKBR3 24H-radicicol-3.33	8/144	2.41×10^{-5}	3.44×10^{-4}
LJP005 PC3 24H-radicicol-10	7/105	2.45×10^{-5}	3.49×10^{-4}
LJP006 HS578T 3H-radicicol-10	6/72	2.68×10^{-5}	3.77×10^{-4}
LJP006 HS578T 3H-radicicol-0.12	4/22	2.77×10^{-5}	3.88×10^{-4}
LJP005 HCC515 24H-radicicol-1.11	6/75	3.38×10^{-5}	4.60×10^{-4}
LJP006 PC3 24H-radicicol-1.11	6/75	3.38×10^{-5}	4.58×10^{-4}
LJP005 MDAMB231 3H-radicicol-3.33	4/24	3.98×10^{-5}	5.21×10^{-4}
LJP006 PC3 24H-radicicol-3.33	6/78	4.23×10^{-5}	5.50×10^{-4}
LJP005 A375 24H-radicicol-10	5/48	4.36×10^{-5}	5.66×10^{-4}
LJP005 MCF10A 3H-radicicol-0.12	5/48	4.36×10^{-5}	5.66×10^{-4}
LJP005 HCC515 24H-radicicol-0.37	6/80	4.88×10^{-5}	6.21×10^{-4}
LJP006 MCF10A 3H-radicicol-10	6/82	5.61×10^{-5}	6.99×10^{-4}
LJP005 HA1E 24H-radicicol-10	6/85	6.87×10^{-5}	8.30×10^{-4}
LJP005 SKBR3 3H-radicicol-3.33	5/53	7.07×10^{-5}	8.49×10^{-4}
LJP005 HEPG2 24H-radicicol-1.11	8/168	7.23×10^{-5}	8.63×10^{-4}
LJP006 HEPG2 24H-radicicol-1.11	6/86	7.33×10^{-5}	8.74×10^{-4}
LJP005 MDAMB231 3H-radicicol-10	5/54	7.75×10^{-5}	9.16×10^{-4}
LJP006 A375 24H-radicicol-1.11	7/132	1.05×10^{-4}	1.18×10^{-3}
LJP006 HME1 24H-radicicol-0.37	7/132	1.05×10^{-4}	1.18×10^{-3}
LJP005 BT20 24H-radicicol-0.12	5/59	1.19×10^{-4}	1.30×10^{-3}
LJP006 MDAMB231 24H-radicicol-1.11	6/97	1.43×10^{-4}	1.52×10^{-3}
LJP006 HCC515 24H-radicicol-3.33	6/98	1.52×10^{-4}	1.59×10^{-3}
LJP006 HME1 3H-radicicol-1.11	5/64	1.75×10^{-4}	1.81×10^{-3}
LJP005 HS578T 3H-radicicol-10	4/35	1.83×10^{-4}	1.88×10^{-3}
LJP005 HS578T 3H-radicicol-1.11	4/36	2.04×10^{-4}	2.06×10^{-3}
LJP005 SKBR3 3H-radicicol-0.12	5/69	2.49×10^{-4}	2.45×10^{-3}
LJP005 MCF7 24H-radicicol-1.11	6/108	2.58×10^{-4}	2.51×10^{-3}
LJP005 SKBR3 24H-radicicol-0.04	5/71	2.85×10^{-4}	2.73×10^{-3}
LJP006 HCC515 24H-radicicol-0.37	4/40	3.09×10^{-4}	2.93×10^{-3}
LJP006 HS578T 3H-radicicol-1.11	4/40	3.09×10^{-4}	2.93×10^{-3}
LJP005 MCF7 24H-radicicol-0.12	4/41	3.40×10^{-4}	3.17×10^{-3}
LJP006 BT20 24H-radicicol-0.12	4/42	3.74×10^{-4}	3.42×10^{-3}
LJP006 MCF7 24H-radicicol-10	8/216	4.03×10^{-4}	3.65×10^{-3}
LJP006 MDAMB231 3H-radicicol-10	4/46	5.31×10^{-4}	4.59×10^{-3}
LJP006 SKBR3 3H-radicicol-0.37	3/20	5.47×10^{-4}	4.71×10^{-3}
LJP006 HEPG2 24H-radicicol-0.37	4/47	5.77×10^{-4}	4.92×10^{-3}
LJP005 MCF10A 24H-radicicol-3.33	7/175	5.90×10^{-4}	5.00×10^{-3}
LJP006 BT20 3H-radicicol-10	3/21	6.35×10^{-4}	5.31×10^{-3}
LJP006 A549 24H-radicicol-0.12	3/23	8.35×10^{-4}	6.72×10^{-3}
LJP005 SKBR3 3H-radicicol-1.11	4/52	8.49×10^{-4}	6.80×10^{-3}
LJP006 SKBR3 3H-radicicol-0.12	3/25	1.07×10^{-3}	8.26×10^{-3}
LJP006 HCC515 24H-radicicol-1.11	5/96	1.14×10^{-3}	8.70×10^{-3}
LJP005 BT20 24H-radicicol-10	6/149	1.41×10^{-3}	1.04×10^{-2}
LJP005 BT20 24H-radicicol-3.33	4/60	1.45×10^{-3}	1.07×10^{-2}
LJP006 MDAMB231 3H-radicicol-1.11	3/29	1.66×10^{-3}	1.19×10^{-2}
LJP005 A375 24H-radicicol-0.12	4/65	1.96×10^{-3}	1.37×10^{-2}
LJP006 HA1E 24H-radicicol-0.37	4/67	2.19×10^{-3}	1.50×10^{-2}
LJP005 HA1E 24H-radicicol-0.12	3/33	2.42×10^{-3}	1.63×10^{-2}
LJP006 A549 24H-radicicol-0.37	3/33	2.42×10^{-3}	1.63×10^{-2}
LJP006 HME1 24H-radicicol-10	5/114	2.43×10^{-3}	1.63×10^{-2}
LJP006 LNCAP 24H-radicicol-0.37	5/119	2.93×10^{-3}	1.90×10^{-2}
LJP006 HME1 3H-radicicol-0.12	3/36	3.11×10^{-3}	2.00×10^{-2}
LJP006 MDAMB231 3H-radicicol-3.33	3/36	3.11×10^{-3}	2.00×10^{-2}
LJP006 BT20 24H-radicicol-10	5/122	3.26×10^{-3}	2.08×10^{-2}
LJP005 PC3 24H-radicicol-3.33	4/77	3.63×10^{-3}	2.27×10^{-2}

S13 Table: (Continued)

LJP005 BT20 24H-radicicol-1.11	4/78	3.80×10^{-3}	2.36×10^{-2}
LJP005 MDAMB231 24H-radicicol-10	3/39	3.92×10^{-3}	2.41×10^{-2}
LJP005 HS578T 24H-radicicol-0.37	3/40	4.21×10^{-3}	2.55×10^{-2}
LJP005 HEPG2 24H-radicicol-0.37	4/81	4.35×10^{-3}	2.62×10^{-2}
LJP006 LNCAP 3H-radicicol-0.37	3/41	4.51×10^{-3}	2.70×10^{-2}
LJP006 HME1 3H-radicicol-10	4/85	5.16×10^{-3}	3.01×10^{-2}
LJP005 MDAMB231 3H-radicicol-0.12	3/44	5.51×10^{-3}	3.16×10^{-2}
LJP006 MCF10A 3H-radicicol-0.04	3/47	6.63×10^{-3}	3.64×10^{-2}
LJP005 SKBR3 3H-radicicol-0.04	3/50	7.87×10^{-3}	4.18×10^{-2}
LJP006 SKBR3 24H-radicicol-10	5/151	7.98×10^{-3}	4.23×10^{-2}
LJP005 HS578T 3H-radicicol-0.04	2/17	8.29×10^{-3}	4.37×10^{-2}
LJP005 MCF7 24H-radicicol-10	6/219	9.23×10^{-3}	4.76×10^{-2}
LJP005 HCC515 24H-radicicol-3.33	4/101	9.42×10^{-3}	4.82×10^{-2}
LJP006 HME1 24H-radicicol-0.12	4/101	9.42×10^{-3}	4.81×10^{-2}
