# Supplementary material for: A new advanced in silico drug discovery method for novel coronavirus (SARS-CoV-2) with tensor decomposition-based unsupervised feature extraction
Source: PLoS One. 2020 Sep 11;15(9):e0238907. doi: 10.1371/journal.pone.0238907 (PMC7485840; doi:10.1371/journal.pone.0238907)
Supplement: S14 Table — A-443654 significantly affects the expression of the selected 163 genes as evident in the “LINCS L1000 Chem Pert up” category in Enrichr. The last number after the—is dose density. (PDF) [file pone.0238907.s014.pdf]

S14 Table: A-443654 significantly affects the expression of the selected 163 genes as evident in the “LINCS L1000 Chem Pert up” category in Enrichr. The last number after the - is dose density.

| Term | Overlap | P-value | Adjusted P-value |
|---|---|---|---|
| LINCS L1000 Chem Pert up | | | |
| LJP006 HCC515 24H-A443654-0.37 | 9/145 | $2.99 \times 10^{-6}$ | $6.13 \times 10^{-5}$ |
| LJP006 HCC515 24H-A443654-0.12 | 7/88 | $7.66 \times 10^{-6}$ | $1.33 \times 10^{-4}$ |
| LJP006 MCF10A 3H-A443654-0.37 | 6/67 | $1.77 \times 10^{-5}$ | $2.66 \times 10^{-4}$ |
| LJP006 PC3 24H-A443654-1.11 | 9/200 | $3.97 \times 10^{-5}$ | $5.32 \times 10^{-4}$ |
| LJP006 HCC515 24H-A443654-10 | 9/203 | $4.45 \times 10^{-5}$ | $5.87 \times 10^{-4}$ |
| LJP006 HS578T 3H-A443654-1.11 | 5/54 | $7.75 \times 10^{-5}$ | $9.27 \times 10^{-4}$ |
| LJP006 HT29 24H-A443654-1.11 | 7/126 | $7.87 \times 10^{-5}$ | $9.36 \times 10^{-4}$ |
| LJP006 MCF7 3H-A443654-0.12 | 6/89 | $8.89 \times 10^{-5}$ | $1.04 \times 10^{-3}$ |
| LJP006 MCF7 3H-A443654-10 | 4/33 | $1.45 \times 10^{-4}$ | $1.56 \times 10^{-3}$ |
| LJP006 MCF7 3H-A443654-3.33 | 5/64 | $1.75 \times 10^{-4}$ | $1.82 \times 10^{-3}$ |
| LJP006 HT29 24H-A443654-0.37 | 4/41 | $3.40 \times 10^{-4}$ | $3.18 \times 10^{-3}$ |
| LJP006 SKBR3 3H-A443654-0.37 | 4/42 | $3.74 \times 10^{-4}$ | $3.44 \times 10^{-3}$ |
| LJP006 MCF7 24H-A443654-0.37 | 6/118 | $4.15 \times 10^{-4}$ | $3.76 \times 10^{-3}$ |
| LJP006 LNCAP 24H-A443654-3.33 | 6/119 | $4.34 \times 10^{-4}$ | $3.91 \times 10^{-3}$ |
| LJP006 A549 24H-A443654-10 | 7/169 | $4.80 \times 10^{-4}$ | $4.24 \times 10^{-3}$ |
| LJP006 MDAMB231 3H-A443654-0.37 | 4/47 | $5.77 \times 10^{-4}$ | $4.95 \times 10^{-3}$ |
| LJP006 PC3 24H-A443654-0.37 | 5/83 | $5.88 \times 10^{-4}$ | $5.02 \times 10^{-3}$ |
| LJP006 MCF7 24H-A443654-0.12 | 5/91 | $8.94 \times 10^{-4}$ | $7.15 \times 10^{-3}$ |
| LJP006 MCF7 24H-A443654-3.33 | 5/92 | $9.40 \times 10^{-4}$ | $7.45 \times 10^{-3}$ |
| LJP006 MCF7 24H-A443654-1.11 | 5/95 | $1.09 \times 10^{-3}$ | $8.35 \times 10^{-3}$ |
| LJP006 BT20 3H-A443654-0.12 | 4/56 | $1.12 \times 10^{-3}$ | $8.59 \times 10^{-3}$ |
| LJP006 A549 24H-A443654-1.11 | 6/150 | $1.46 \times 10^{-3}$ | $1.06 \times 10^{-2}$ |
| LJP006 MDAMB231 3H-A443654-0.12 | 3/30 | $1.84 \times 10^{-3}$ | $1.28 \times 10^{-2}$ |
| LJP006 LNCAP 24H-A443654-0.37 | 5/108 | $1.92 \times 10^{-3}$ | $1.33 \times 10^{-2}$ |
| LJP006 BT20 24H-A443654-0.37 | 6/164 | $2.29 \times 10^{-3}$ | $1.54 \times 10^{-2}$ |
| LJP006 BT20 24H-A443654-1.11 | 5/113 | $2.34 \times 10^{-3}$ | $1.56 \times 10^{-2}$ |
| LJP006 HME1 24H-A443654-0.04 | 4/70 | $2.57 \times 10^{-3}$ | $1.70 \times 10^{-2}$ |
| LJP006 SKBR3 24H-A443654-0.37 | 5/116 | $2.62 \times 10^{-3}$ | $1.73 \times 10^{-2}$ |
| LJP006 BT20 24H-A443654-3.33 | 5/117 | $2.72 \times 10^{-3}$ | $1.77 \times 10^{-2}$ |
| LJP006 SKBR3 24H-A443654-0.04 | 4/74 | $3.14 \times 10^{-3}$ | $1.99 \times 10^{-2}$ |
| LJP006 HEPG2 24H-A443654-0.37 | 4/81 | $4.35 \times 10^{-3}$ | $2.57 \times 10^{-2}$ |
| LJP006 MCF7 3H-A443654-0.37 | 3/42 | $4.83 \times 10^{-3}$ | $2.79 \times 10^{-2}$ |
| LJP006 SKBR3 3H-A443654-3.33 | 3/43 | $5.16 \times 10^{-3}$ | $2.93 \times 10^{-2}$ |
| LJP006 HCC515 24H-A443654-3.33 | 6/211 | $7.76 \times 10^{-3}$ | $4.06 \times 10^{-2}$ |
| LJP006 LNCAP 3H-A443654-10 | 3/50 | $7.87 \times 10^{-3}$ | $4.09 \times 10^{-2}$ |
| LJP006 HT29 24H-A443654-3.33 | 5/155 | $8.87 \times 10^{-3}$ | $4.48 \times 10^{-2}$ |
