# Supplementary material for: A new advanced in silico drug discovery method for novel coronavirus (SARS-CoV-2) with tensor decomposition-based unsupervised feature extraction
Source: PLoS One. 2020 Sep 11;15(9):e0238907. doi: 10.1371/journal.pone.0238907 (PMC7485840; doi:10.1371/journal.pone.0238907)
Supplement: S15 Table — A-443654 significantly affects the expression of the selected 163 genes as evident in the “LINCS L1000 Chem Pert up” category in Enrichr. The last number after the—is dose density. (PDF) [file pone.0238907.s015.pdf]

S15 Table: A-443654 significantly affects the expression of the selected 163 genes due to "LINCS L1000 Chem Pert down" category in Enrichr. The last number after the - is dose density.

Term	Overlap	P-value	Adjusted P-value
LINCS L1000 Chem Pert down			
LJP006 BT20 24H-A443654-1.11	19/117	1.22×10^{-19}	1.01×10^{-15}
LJP006 SKBR3 24H-A443654-3.33	16/114	1.16×10^{-15}	1.04×10^{-12}
LJP006 HEPG2 24H-A443654-10	14/102	1.04×10^{-13}	3.99×10^{-11}
LJP006 BT20 24H-A443654-3.33	13/92	5.52×10^{-13}	1.59×10^{-10}
LJP006 HCC515 24H-A443654-1.11	15/145	9.24×10^{-13}	2.49×10^{-10}
LJP006 SKBR3 24H-A443654-1.11	13/119	1.60×10^{-11}	2.31×10^{-9}
LJP006 HA1E 24H-A443654-0.04	13/139	1.16×10^{-10}	1.18×10^{-8}
LJP006 HA1E 24H-A443654-1.11	12/114	1.54×10^{-10}	1.49×10^{-8}
LJP006 HA1E 24H-A443654-0.37	13/147	2.34×10^{-10}	2.12×10^{-8}
LJP006 MCF10A 3H-A443654-10	11/101	6.34×10^{-10}	4.83×10^{-8}
LJP006 LNCAP 24H-A443654-1.11	12/132	8.57×10^{-10}	6.19×10^{-8}
LJP006 PC3 24H-A443654-1.11	13/175	2.03×10^{-9}	1.27×10^{-7}
LJP006 LNCAP 24H-A443654-0.12	10/86	2.04×10^{-9}	1.27×10^{-7}
LJP006 HCC515 24H-A443654-10	11/114	2.34×10^{-9}	1.42×10^{-7}
LJP006 HA1E 24H-A443654-0.12	11/125	6.25×10^{-9}	3.24×10^{-7}
LJP006 MCF10A 24H-A443654-0.37	10/107	1.75×10^{-8}	7.66×10^{-7}
LJP006 HCC515 24H-A443654-3.33	10/111	2.49×10^{-8}	1.02×10^{-6}
LJP006 BT20 24H-A443654-10	9/83	2.49×10^{-8}	1.02×10^{-6}
LJP006 HEPG2 24H-A443654-3.33	10/117	4.13×10^{-8}	1.59×10^{-6}
LJP006 MDAMB231 3H-A443654-10	10/118	4.49×10^{-8}	1.71×10^{-6}
LJP006 HA1E 24H-A443654-3.33	8/64	4.92×10^{-8}	1.85×10^{-6}
LJP006 A375 24H-A443654-1.11	8/68	8.00×10^{-8}	2.78×10^{-6}
LJP006 A549 24H-A443654-10	8/70	1.01×10^{-7}	3.36×10^{-6}
LJP006 MCF10A 3H-A443654-1.11	8/72	1.26×10^{-7}	4.08×10^{-6}
LJP006 BT20 24H-A443654-0.04	9/112	3.41×10^{-7}	9.39×10^{-6}
LJP006 HME1 24H-A443654-3.33	7/57	3.94×10^{-7}	1.06×10^{-5}
LJP006 HS578T 24H-A443654-0.12	8/85	4.63×10^{-7}	1.20×10^{-5}
LJP006 HME1 3H-A443654-0.12	6/37	5.04×10^{-7}	1.29×10^{-5}
LJP006 HME1 24H-A443654-1.11	7/61	6.33×10^{-7}	1.56×10^{-5}
LJP006 HME1 24H-A443654-10	6/39	6.98×10^{-7}	1.70×10^{-5}
LJP006 LNCAP 24H-A443654-3.33	7/63	7.91×10^{-7}	1.89×10^{-5}
LJP006 PC3 24H-A443654-3.33	9/124	8.10×10^{-7}	1.93×10^{-5}
LJP006 HME1 3H-A443654-3.33	8/93	9.27×10^{-7}	2.17×10^{-5}
LJP006 HME1 3H-A443654-1.11	9/142	2.52×10^{-6}	4.99×10^{-5}
LJP006 HME1 24H-A443654-0.04	7/75	2.62×10^{-6}	5.16×10^{-5}
LJP006 MDAMB231 3H-A443654-0.37	6/49	2.80×10^{-6}	5.46×10^{-5}
LJP006 SKBR3 24H-A443654-0.04	7/77	3.13×10^{-6}	6.00×10^{-5}
LJP006 HS578T 24H-A443654-0.04	6/52	3.99×10^{-6}	7.41×10^{-5}
LJP006 HS578T 24H-A443654-0.37	8/115	4.63×10^{-6}	8.43×10^{-5}
LJP006 BT20 3H-A443654-1.11	6/54	4.99×10^{-6}	8.98×10^{-5}
LJP006 SKBR3 3H-A443654-3.33	6/56	6.19×10^{-6}	1.07×10^{-4}
LJP006 SKBR3 3H-A443654-1.11	6/56	6.19×10^{-6}	1.07×10^{-4}
LJP006 LNCAP 3H-A443654-1.11	6/58	7.62×10^{-6}	1.29×10^{-4}
LJP006 SKBR3 24H-A443654-0.37	8/126	9.11×10^{-6}	1.49×10^{-4}
LJP006 MDAMB231 24H-A443654-1.11	6/64	1.36×10^{-5}	2.10×10^{-4}
LJP006 MCF7 3H-A443654-10	6/66	1.62×10^{-5}	2.44×10^{-4}
LJP006 A549 24H-A443654-0.12	7/99	1.67×10^{-5}	2.50×10^{-4}
LJP006 A549 24H-A443654-1.11	8/137	1.68×10^{-5}	2.51×10^{-4}
LJP006 HS578T 24H-A443654-1.11	6/69	2.10×10^{-5}	3.04×10^{-4}
LJP006 HCC515 24H-A443654-0.12	7/106	2.60×10^{-5}	3.68×10^{-4}
LJP006 HCC515 24H-A443654-0.37	7/106	2.60×10^{-5}	3.67×10^{-4}
LJP006 MDAMB231 24H-A443654-10	5/47	3.93×10^{-5}	5.16×10^{-4}
LJP006 HS578T 3H-A443654-10	7/114	4.16×10^{-5}	5.44×10^{-4}

S15 Table: (Continued)

LJP006 A549 24H-A443654-0.37	8/157	4.48×10^{-5}	5.77×10^{-4}
LJP006 MDAMB231 3H-A443654-1.11	6/80	4.88×10^{-5}	6.20×10^{-4}
LJP006 A375 24H-A443654-0.12	7/120	5.77×10^{-5}	7.18×10^{-4}
LJP006 SKBR3 3H-A443654-10	6/83	6.01×10^{-5}	7.42×10^{-4}
LJP006 BT20 24H-A443654-0.37	7/124	7.11×10^{-5}	8.51×10^{-4}
LJP006 HS578T 3H-A443654-3.33	7/128	8.69×10^{-5}	9.99×10^{-4}
LJP006 MDAMB231 24H-A443654-0.37	6/89	8.89×10^{-5}	1.02×10^{-3}
LJP006 SKBR3 24H-A443654-0.12	7/129	9.12×10^{-5}	1.04×10^{-3}
LJP006 PC3 24H-A443654-10	6/90	9.46×10^{-5}	1.07×10^{-3}
LJP006 BT20 3H-A443654-3.33	4/31	1.13×10^{-4}	1.24×10^{-3}
LJP006 HT29 24H-A443654-1.11	7/134	1.16×10^{-4}	1.27×10^{-3}
LJP006 HME1 24H-A443654-0.37	6/94	1.20×10^{-4}	1.31×10^{-3}
LJP006 LNCAP 24H-A443654-0.37	7/135	1.21×10^{-4}	1.32×10^{-3}
LJP006 SKBR3 3H-A443654-0.12	4/32	1.28×10^{-4}	1.39×10^{-3}
LJP006 HME1 3H-A443654-10	5/60	1.29×10^{-4}	1.39×10^{-3}
LJP006 A375 24H-A443654-3.33	4/35	1.83×10^{-4}	1.87×10^{-3}
LJP006 MCF10A 3H-A443654-0.04	4/36	2.04×10^{-4}	2.05×10^{-3}
LJP006 HS578T 3H-A443654-0.37	5/68	2.33×10^{-4}	2.30×10^{-3}
LJP006 MCF7 24H-A443654-0.12	6/108	2.58×10^{-4}	2.51×10^{-3}
LJP006 BT20 24H-A443654-0.12	6/110	2.85×10^{-4}	2.73×10^{-3}
LJP006 MCF10A 3H-A443654-3.33	6/110	2.85×10^{-4}	2.73×10^{-3}
LJP006 HT29 24H-A443654-10	5/71	2.85×10^{-4}	2.73×10^{-3}
LJP006 MDAMB231 24H-A443654-3.33	5/71	2.85×10^{-4}	2.73×10^{-3}
LJP006 HS578T 3H-A443654-1.11	6/113	3.29×10^{-4}	3.08×10^{-3}
LJP006 MCF7 24H-A443654-10	4/43	4.10×10^{-4}	3.68×10^{-3}
LJP006 HT29 24H-A443654-3.33	5/78	4.42×10^{-4}	3.93×10^{-3}
LJP006 A375 24H-A443654-10	4/44	4.48×10^{-4}	3.98×10^{-3}
LJP006 BT20 3H-A443654-10	4/45	4.88×10^{-4}	4.29×10^{-3}
LJP006 MDAMB231 3H-A443654-3.33	6/123	5.18×10^{-4}	4.51×10^{-3}
LJP006 SKBR3 3H-A443654-0.37	3/20	5.47×10^{-4}	4.71×10^{-3}
LJP006 LNCAP 3H-A443654-10	5/82	5.56×10^{-4}	4.77×10^{-3}
LJP006 MDAMB231 24H-A443654-0.04	4/48	6.26×10^{-4}	5.26×10^{-3}
LJP006 MCF7 24H-A443654-1.11	4/49	6.77×10^{-4}	5.61×10^{-3}
LJP006 LNCAP 3H-A443654-3.33	5/89	8.09×10^{-4}	6.54×10^{-3}
LJP006 MCF7 24H-A443654-0.37	5/91	8.94×10^{-4}	7.14×10^{-3}
LJP006 MCF7 3H-A443654-1.11	5/91	8.94×10^{-4}	7.13×10^{-3}
LJP006 BT20 3H-A443654-0.37	3/25	1.07×10^{-3}	8.27×10^{-3}
LJP006 BT20 3H-A443654-0.04	3/26	1.20×10^{-3}	9.09×10^{-3}
LJP006 MCF10A 3H-A443654-0.37	4/62	1.64×10^{-3}	1.18×10^{-2}
LJP006 MCF7 3H-A443654-3.33	4/66	2.07×10^{-3}	1.43×10^{-2}
LJP006 MDAMB231 24H-A443654-0.12	4/70	2.57×10^{-3}	1.71×10^{-2}
LJP006 HEPG2 24H-A443654-0.37	5/116	2.62×10^{-3}	1.74×10^{-2}
LJP006 LNCAP 3H-A443654-0.37	3/34	2.64×10^{-3}	1.75×10^{-2}
LJP006 HEPG2 24H-A443654-1.11	5/125	3.62×10^{-3}	2.27×10^{-2}
LJP006 HS578T 3H-A443654-0.12	3/39	3.92×10^{-3}	2.41×10^{-2}
LJP006 MCF7 24H-A443654-3.33	3/43	5.16×10^{-3}	2.99×10^{-2}
LJP006 HME1 3H-A443654-0.04	3/48	7.03×10^{-3}	3.82×10^{-2}
LJP006 LNCAP 24H-A443654-0.04	3/48	7.03×10^{-3}	3.82×10^{-2}
LJP006 HT29 24H-A443654-0.37	3/50	7.87×10^{-3}	4.18×10^{-2}
LJP006 LNCAP 3H-A443654-0.04	2/18	9.27×10^{-3}	4.75×10^{-2}
