# Supplementary material for: A new advanced in silico drug discovery method for novel coronavirus (SARS-CoV-2) with tensor decomposition-based unsupervised feature extraction
Source: PLoS One. 2020 Sep 11;15(9):e0238907. doi: 10.1371/journal.pone.0238907 (PMC7485840; doi:10.1371/journal.pone.0238907)
Supplement: S16 Table — CGP-60474 significantly affects the expression of the selected 163 genes due to “LINCS L1000 Chem Pert up” category in Enrichr. The last number after the—is dose density. (PDF) [file pone.0238907.s016.pdf]

S16 Table: CGP-60474 significantly affects the expression of the selected 163 genes due to "LINCS L1000 Chem Pert up" category in Enrichr. The last number after the - is dose density.

Term	Overlap	P-value	Adjusted P-value
LINCS L1000 Chem Pert up			
LJP008 HT29 24H-CGP-60474-0.04	10/117	4.13×10^{-8}	1.70×10^{-6}
LJP005 MCF7 3H-CGP-60474-10	8/69	8.98×10^{-8}	3.29×10^{-6}
LJP006 MCF7 3H-CGP-60474-0.12	7/61	6.33×10^{-7}	1.69×10^{-5}
LJP008 PC3 24H-CGP-60474-0.04	10/161	8.27×10^{-7}	2.11×10^{-5}
LJP008 MCF7 24H-CGP-60474-10	8/94	1.01×10^{-6}	2.48×10^{-5}
LJP005 MCF10A 24H-CGP-60474-0.04	6/42	1.10×10^{-6}	2.67×10^{-5}
LJP006 LNCAP 3H-CGP-60474-0.37	7/68	1.34×10^{-6}	3.14×10^{-5}
LJP007 HCC515 24H-CGP-60474-0.04	9/138	1.99×10^{-6}	4.33×10^{-5}
LJP005 SKBR3 24H-CGP-60474-0.12	8/106	2.51×10^{-6}	5.28×10^{-5}
LJP007 HT29 24H-CGP-60474-0.12	9/146	3.17×10^{-6}	6.38×10^{-5}
LJP005 SKBR3 24H-CGP-60474-0.37	8/115	4.63×10^{-6}	8.82×10^{-5}
LJP009 PC3 24H-CGP-60474-0.04	10/198	5.34×10^{-6}	9.96×10^{-5}
LJP006 HCC515 24H-CGP-60474-0.37	10/225	1.63×10^{-5}	2.49×10^{-4}
LJP009 MCF7 24H-CGP-60474-0.12	8/137	1.68×10^{-5}	2.55×10^{-4}
LJP006 MCF7 3H-CGP-60474-3.33	6/67	1.77×10^{-5}	2.66×10^{-4}
LJP007 MCF7 24H-CGP-60474-0.04	8/144	2.41×10^{-5}	3.48×10^{-4}
LJP007 PC3 24H-CGP-60474-10	7/122	6.41×10^{-5}	7.96×10^{-4}
LJP008 A375 24H-CGP-60474-0.37	8/170	7.85×10^{-5}	9.36×10^{-4}
LJP009 MCF7 24H-CGP-60474-10	7/126	7.87×10^{-5}	9.37×10^{-4}
LJP005 A549 24H-CGP-60474-0.37	8/171	8.18×10^{-5}	9.72×10^{-4}
LJP009 PC3 24H-CGP-60474-1.11	9/220	8.28×10^{-5}	9.82×10^{-4}
LJP009 PC3 24H-CGP-60474-10	8/176	1.00×10^{-4}	1.15×10^{-3}
LJP009 PC3 24H-CGP-60474-0.12	8/176	1.00×10^{-4}	1.15×10^{-3}
LJP007 PC3 24H-CGP-60474-3.33	7/131	1.01×10^{-4}	1.16×10^{-3}
LJP005 MCF7 3H-CGP-60474-3.33	5/58	1.09×10^{-4}	1.24×10^{-3}
LJP005 PC3 24H-CGP-60474-1.11	8/183	1.31×10^{-4}	1.43×10^{-3}
LJP009 HT29 24H-CGP-60474-1.11	6/99	1.60×10^{-4}	1.70×10^{-3}
LJP006 A375 24H-CGP-60474-0.37	7/144	1.81×10^{-4}	1.88×10^{-3}
LJP006 MCF10A 24H-CGP-60474-10	7/148	2.14×10^{-4}	2.16×10^{-3}
LJP006 MCF7 3H-CGP-60474-10	5/67	2.17×10^{-4}	2.18×10^{-3}
LJP006 A549 24H-CGP-60474-10	7/149	2.23×10^{-4}	2.23×10^{-3}
LJP008 A549 24H-CGP-60474-0.37	6/106	2.33×10^{-4}	2.31×10^{-3}
LJP005 MCF7 3H-CGP-60474-0.37	6/106	2.33×10^{-4}	2.31×10^{-3}
LJP006 BT20 24H-CGP-60474-3.33	6/110	2.85×10^{-4}	2.74×10^{-3}
LJP008 HCC515 24H-CGP-60474-3.33	7/157	3.08×10^{-4}	2.93×10^{-3}
LJP009 A375 24H-CGP-60474-0.12	6/112	3.14×10^{-4}	2.97×10^{-3}
LJP006 LNCAP 24H-CGP-60474-10	7/159	3.32×10^{-4}	3.13×10^{-3}
LJP007 PC3 24H-CGP-60474-0.04	6/114	3.45×10^{-4}	3.21×10^{-3}
LJP005 HCC515 24H-CGP-60474-0.12	5/75	3.68×10^{-4}	3.41×10^{-3}
LJP007 MCF7 24H-CGP-60474-10	6/117	3.97×10^{-4}	3.62×10^{-3}
LJP006 SKBR3 3H-CGP-60474-0.37	4/43	4.10×10^{-4}	3.71×10^{-3}
LJP009 MCF7 24H-CGP-60474-0.04	6/119	4.34×10^{-4}	3.91×10^{-3}
LJP008 HT29 24H-CGP-60474-1.11	5/78	4.42×10^{-4}	3.97×10^{-3}
LJP006 SKBR3 24H-CGP-60474-3.33	6/120	4.54×10^{-4}	4.05×10^{-3}
LJP008 MCF7 24H-CGP-60474-1.11	6/121	4.75×10^{-4}	4.21×10^{-3}
LJP008 HEPG2 24H-CGP-60474-0.37	6/122	4.96×10^{-4}	4.36×10^{-3}
LJP005 PC3 24H-CGP-60474-0.37	7/171	5.14×10^{-4}	4.51×10^{-3}
LJP007 HCC515 24H-CGP-60474-0.12	7/179	6.75×10^{-4}	5.67×10^{-3}
LJP007 PC3 24H-CGP-60474-0.12	5/87	7.29×10^{-4}	6.05×10^{-3}
LJP009 HCC515 24H-CGP-60474-0.12	5/90	8.51×10^{-4}	6.83×10^{-3}
LJP008 MCF7 24H-CGP-60474-0.37	5/91	8.94×10^{-4}	7.15×10^{-3}
LJP006 PC3 24H-CGP-60474-1.11	6/137	9.12×10^{-4}	7.28×10^{-3}
LJP005 HS578T 3H-CGP-60474-0.04	4/53	9.12×10^{-4}	7.26×10^{-3}
LJP005 MCF10A 3H-CGP-60474-0.04	4/53	9.12×10^{-4}	7.25×10^{-3}

S16 Table: (Continued)

LJP006 LNCAP 3H-CGP-60474-1.11	4/53	9.12×10^{-4}	7.24×10^{-3}
LJP006 MCF10A 24H-CGP-60474-1.11	6/138	9.47×10^{-4}	7.50×10^{-3}
LJP008 HT29 24H-CGP-60474-0.12	4/55	1.05×10^{-3}	8.15×10^{-3}
LJP006 HCC515 24H-CGP-60474-1.11	7/194	1.08×10^{-3}	8.34×10^{-3}
LJP005 HCC515 24H-CGP-60474-1.11	6/142	1.10×10^{-3}	8.44×10^{-3}
LJP007 MCF7 24H-CGP-60474-0.37	6/142	1.10×10^{-3}	8.43×10^{-3}
LJP006 HME1 3H-CGP-60474-1.11	4/57	1.20×10^{-3}	9.08×10^{-3}
LJP007 MCF7 24H-CGP-60474-1.11	6/145	1.22×10^{-3}	9.19×10^{-3}
LJP007 HT29 24H-CGP-60474-0.37	6/147	1.31×10^{-3}	9.74×10^{-3}
LJP006 SKBR3 24H-CGP-60474-1.11	5/100	1.37×10^{-3}	1.01×10^{-2}
LJP005 MCF7 3H-CGP-60474-0.12	4/60	1.45×10^{-3}	1.06×10^{-2}
LJP006 A375 24H-CGP-60474-1.11	6/156	1.78×10^{-3}	1.25×10^{-2}
LJP006 MCF10A 24H-CGP-60474-3.33	6/161	2.08×10^{-3}	1.42×10^{-2}
LJP005 HS578T 3H-CGP-60474-10	4/67	2.19×10^{-3}	1.48×10^{-2}
LJP006 PC3 24H-CGP-60474-0.37	7/221	2.27×10^{-3}	1.53×10^{-2}
LJP006 MDAMB231 3H-CGP-60474-0.37	4/68	2.31×10^{-3}	1.55×10^{-2}
LJP008 MCF7 24H-CGP-60474-0.12	5/115	2.52×10^{-3}	1.67×10^{-2}
LJP006 A375 24H-CGP-60474-10	5/118	2.82×10^{-3}	1.84×10^{-2}
LJP007 MCF7 24H-CGP-60474-0.12	5/119	2.93×10^{-3}	1.88×10^{-2}
LJP008 PC3 24H-CGP-60474-10	5/120	3.04×10^{-3}	1.94×10^{-2}
LJP005 A375 24H-CGP-60474-3.33	6/174	3.07×10^{-3}	1.96×10^{-2}
LJP006 SKBR3 3H-CGP-60474-1.11	3/36	3.11×10^{-3}	1.98×10^{-2}
LJP006 HS578T 3H-CGP-60474-3.33	4/74	3.14×10^{-3}	1.99×10^{-2}
LJP008 A375 24H-CGP-60474-0.12	6/175	3.16×10^{-3}	2.00×10^{-2}
LJP008 PC3 24H-CGP-60474-3.33	6/177	3.34×10^{-3}	2.10×10^{-2}
LJP005 HS578T 24H-CGP-60474-0.37	5/124	3.49×10^{-3}	2.17×10^{-2}
LJP008 A375 24H-CGP-60474-10	6/179	3.53×10^{-3}	2.19×10^{-2}
LJP005 MCF7 3H-CGP-60474-1.11	4/78	3.80×10^{-3}	2.32×10^{-2}
LJP006 A549 24H-CGP-60474-1.11	5/129	4.14×10^{-3}	2.48×10^{-2}
LJP006 BT20 24H-CGP-60474-0.12	5/129	4.14×10^{-3}	2.48×10^{-2}
LJP006 HME1 3H-CGP-60474-0.12	4/80	4.16×10^{-3}	2.49×10^{-2}
LJP005 MDAMB231 3H-CGP-60474-10	3/40	4.21×10^{-3}	2.51×10^{-2}
LJP006 MCF10A 3H-CGP-60474-1.11	3/40	4.21×10^{-3}	2.50×10^{-2}
LJP009 MCF7 24H-CGP-60474-3.33	5/131	4.41×10^{-3}	2.61×10^{-2}
LJP008 PC3 24H-CGP-60474-0.12	5/133	4.71×10^{-3}	2.74×10^{-2}
LJP005 PC3 24H-CGP-60474-3.33	5/134	4.86×10^{-3}	2.80×10^{-2}
LJP006 SKBR3 24H-CGP-60474-0.04	5/134	4.86×10^{-3}	2.80×10^{-2}
LJP006 HS578T 3H-CGP-60474-0.04	3/44	5.51×10^{-3}	3.09×10^{-2}
LJP009 PC3 24H-CGP-60474-0.37	6/200	6.03×10^{-3}	3.31×10^{-2}
LJP006 HME1 24H-CGP-60474-0.12	5/142	6.19×10^{-3}	3.39×10^{-2}
LJP005 HT29 24H-CGP-60474-1.11	5/143	6.37×10^{-3}	3.46×10^{-2}
LJP008 A549 24H-CGP-60474-0.04	4/91	6.56×10^{-3}	3.55×10^{-2}
LJP009 HCC515 24H-CGP-60474-0.04	4/93	7.08×10^{-3}	3.76×10^{-2}
LJP005 BT20 24H-CGP-60474-0.12	3/49	7.44×10^{-3}	3.93×10^{-2}
LJP008 A375 24H-CGP-60474-1.11	5/151	7.98×10^{-3}	4.12×10^{-2}
LJP006 HS578T 24H-CGP-60474-1.11	5/151	7.98×10^{-3}	4.12×10^{-2}
LJP008 A375 24H-CGP-60474-3.33	5/152	8.19×10^{-3}	4.22×10^{-2}
LJP005 A549 24H-CGP-60474-0.04	2/17	8.29×10^{-3}	4.26×10^{-2}
LJP007 A549 24H-CGP-60474-0.04	3/51	8.31×10^{-3}	4.25×10^{-2}
LJP008 A549 24H-CGP-60474-10	5/153	8.42×10^{-3}	4.30×10^{-2}
LJP009 PC3 24H-CGP-60474-3.33	6/217	8.84×10^{-3}	4.46×10^{-2}
LJP009 HT29 24H-CGP-60474-0.04	4/100	9.10×10^{-3}	4.58×10^{-2}
LJP007 HT29 24H-CGP-60474-10	5/156	9.11×10^{-3}	4.58×10^{-2}
LJP008 PC3 24H-CGP-60474-1.11	5/157	9.35×10^{-3}	4.66×10^{-2}
LJP006 HCC515 24H-CGP-60474-10	6/221	9.62×10^{-3}	4.78×10^{-2}
LJP009 MCF7 24H-CGP-60474-1.11	5/159	9.84×10^{-3}	4.86×10^{-2}
LJP008 PC3 24H-CGP-60474-0.37	5/160	1.01×10^{-2}	4.96×10^{-2}
