# Supplementary material for: A new advanced in silico drug discovery method for novel coronavirus (SARS-CoV-2) with tensor decomposition-based unsupervised feature extraction
Source: PLoS One. 2020 Sep 11;15(9):e0238907. doi: 10.1371/journal.pone.0238907 (PMC7485840; doi:10.1371/journal.pone.0238907)
Supplement: S17 Table — CGP-60474 significantly affects the expression of the selected 163 genes due to “LINCS L1000 Chem Pert down” category in Enrichr. The last number after the—is dose density. (PDF) [file pone.0238907.s017.pdf]

S17 Table: CGP-60474 significantly affects the expression of the selected 163 genes as evident in the “LINCS L1000 Chem Pert down” category in Enrichr. The last number after the - is dose density.

Term	Overlap	P-value	Adjusted P-value
LINCS L1000 Chem Pert down			
LJP006 SKBR3 24H-CGP-60474-1.11	18/106	5.03×10^{-19}	3.33×10^{-15}
LJP006 SKBR3 24H-CGP-60474-10	18/118	3.76×10^{-18}	1.56×10^{-14}
LJP007 A549 24H-CGP-60474-0.37	12/30	4.34×10^{-18}	1.60×10^{-14}
LJP005 HS578T 24H-CGP-60474-3.33	17/131	5.56×10^{-16}	5.94×10^{-13}
LJP006 SKBR3 24H-CGP-60474-0.04	16/113	1.01×10^{-15}	9.51×10^{-13}
LJP006 SKBR3 24H-CGP-60474-0.37	15/104	6.17×10^{-15}	4.44×10^{-12}
LJP009 A549 24H-CGP-60474-0.04	13/67	7.47×10^{-15}	4.95×10^{-12}
LJP006 SKBR3 24H-CGP-60474-3.33	15/130	1.82×10^{-13}	6.42×10^{-11}
LJP006 MCF10A 24H-CGP-60474-0.12	13/88	3.05×10^{-13}	9.91×10^{-11}
LJP006 HME1 3H-CGP-60474-1.11	16/165	4.30×10^{-13}	1.31×10^{-10}
LJP005 BT20 24H-CGP-60474-10	13/93	6.37×10^{-13}	1.80×10^{-10}
LJP008 HCC515 24H-CGP-60474-0.12	13/101	1.89×10^{-12}	4.30×10^{-10}
LJP007 HCC515 24H-CGP-60474-10	12/95	1.74×10^{-11}	2.46×10^{-9}
LJP006 MCF10A 3H-CGP-60474-0.37	13/127	3.68×10^{-11}	4.67×10^{-9}
LJP006 MCF10A 24H-CGP-60474-3.33	12/102	4.09×10^{-11}	5.12×10^{-9}
LJP006 BT20 24H-CGP-60474-10	13/134	7.29×10^{-11}	8.16×10^{-9}
LJP009 A549 24H-CGP-60474-0.12	10/63	8.63×10^{-11}	9.25×10^{-9}
LJP006 HEPG2 24H-CGP-60474-10	12/109	9.04×10^{-11}	9.54×10^{-9}
LJP006 BT20 24H-CGP-60474-1.11	10/65	1.19×10^{-10}	1.21×10^{-8}
LJP005 BT20 24H-CGP-60474-3.33	10/66	1.40×10^{-10}	1.38×10^{-8}
LJP005 HS578T 24H-CGP-60474-10	12/114	1.54×10^{-10}	1.50×10^{-8}
LJP005 SKBR3 24H-CGP-60474-3.33	12/114	1.54×10^{-10}	1.49×10^{-8}
LJP008 A549 24H-CGP-60474-0.12	10/67	1.63×10^{-10}	1.56×10^{-8}
LJP005 A549 24H-CGP-60474-0.12	15/211	2.06×10^{-10}	1.90×10^{-8}
LJP008 HEPG2 24H-CGP-60474-10	12/121	3.11×10^{-10}	2.69×10^{-8}
LJP006 SKBR3 24H-CGP-60474-0.12	11/103	7.84×10^{-10}	5.76×10^{-8}
LJP006 A549 24H-CGP-60474-1.11	9/57	8.18×10^{-10}	5.94×10^{-8}
LJP007 HT29 24H-CGP-60474-0.37	11/105	9.66×10^{-10}	6.82×10^{-8}
LJP008 HEPG2 24H-CGP-60474-0.12	12/134	1.02×10^{-9}	7.13×10^{-8}
LJP009 HT29 24H-CGP-60474-0.37	9/59	1.13×10^{-9}	7.77×10^{-8}
LJP007 A549 24H-CGP-60474-1.11	8/41	1.25×10^{-9}	8.45×10^{-8}
LJP005 A549 24H-CGP-60474-0.37	10/82	1.27×10^{-9}	8.54×10^{-8}
LJP009 A549 24H-CGP-60474-10	9/61	1.53×10^{-9}	9.96×10^{-8}
LJP005 BT20 24H-CGP-60474-0.12	10/84	1.61×10^{-9}	1.03×10^{-7}
LJP006 LNCAP 24H-CGP-60474-0.04	12/140	1.69×10^{-9}	1.07×10^{-7}
LJP006 MCF10A 24H-CGP-60474-10	10/86	2.04×10^{-9}	1.26×10^{-7}
LJP006 SKBR3 3H-CGP-60474-1.11	10/87	2.29×10^{-9}	1.40×10^{-7}
LJP005 SKBR3 3H-CGP-60474-1.11	10/89	2.86×10^{-9}	1.68×10^{-7}
LJP005 SKBR3 24H-CGP-60474-0.37	10/91	3.57×10^{-9}	2.03×10^{-7}
LJP006 MCF10A 24H-CGP-60474-1.11	10/91	3.57×10^{-9}	2.02×10^{-7}
LJP007 HEPG2 24H-CGP-60474-1.11	10/91	3.57×10^{-9}	2.02×10^{-7}
LJP007 HT29 24H-CGP-60474-1.11	10/91	3.57×10^{-9}	2.02×10^{-7}
LJP006 HS578T 3H-CGP-60474-10	12/150	3.73×10^{-9}	2.09×10^{-7}
LJP006 BT20 24H-CGP-60474-3.33	10/93	4.43×10^{-9}	2.43×10^{-7}
LJP007 A549 24H-CGP-60474-0.12	7/32	5.95×10^{-9}	3.13×10^{-7}
LJP005 HCC515 24H-CGP-60474-1.11	8/52	9.09×10^{-9}	4.45×10^{-7}
LJP009 A549 24H-CGP-60474-1.11	9/76	1.13×10^{-8}	5.40×10^{-7}
LJP005 MCF10A 24H-CGP-60474-1.11	10/103	1.21×10^{-8}	5.67×10^{-7}
LJP006 HME1 3H-CGP-60474-0.04	12/167	1.26×10^{-8}	5.86×10^{-7}
LJP006 SKBR3 3H-CGP-60474-0.12	9/77	1.28×10^{-8}	5.91×10^{-7}
LJP008 HEPG2 24H-CGP-60474-0.04	11/134	1.30×10^{-8}	6.00×10^{-7}
LJP005 MCF7 24H-CGP-60474-3.33	10/104	1.32×10^{-8}	6.07×10^{-7}
LJP006 HEPG2 24H-CGP-60474-1.11	10/104	1.32×10^{-8}	6.03×10^{-7}
LJP009 HCC515 24H-CGP-60474-0.04	10/108	1.91×10^{-8}	8.22×10^{-7}

S17 Table: (Continued)

LJP005 HT29 24H-CGP-60474-3.33	10/108	1.91×10^{-8}	8.20×10^{-7}
LJP007 HEPG2 24H-CGP-60474-10	9/81	2.01×10^{-8}	8.49×10^{-7}
LJP006 SKBR3 3H-CGP-60474-3.33	9/82	2.24×10^{-8}	9.28×10^{-7}
LJP009 HT29 24H-CGP-60474-3.33	8/61	3.34×10^{-8}	1.32×10^{-6}
LJP006 HS578T 3H-CGP-60474-0.37	10/117	4.13×10^{-8}	1.59×10^{-6}
LJP006 MCF10A 3H-CGP-60474-10	11/151	4.51×10^{-8}	1.71×10^{-6}
LJP005 MCF7 24H-CGP-60474-0.12	9/89	4.63×10^{-8}	1.75×10^{-6}
LJP009 A549 24H-CGP-60474-3.33	9/90	5.11×10^{-8}	1.92×10^{-6}
LJP007 HEPG2 24H-CGP-60474-0.37	9/90	5.11×10^{-8}	1.90×10^{-6}
LJP005 HS578T 24H-CGP-60474-0.12	9/91	5.63×10^{-8}	2.07×10^{-6}
LJP008 PC3 24H-CGP-60474-10	10/121	5.70×10^{-8}	2.09×10^{-6}
LJP006 HME1 3H-CGP-60474-10	10/121	5.70×10^{-8}	2.08×10^{-6}
LJP005 HS578T 3H-CGP-60474-0.37	11/155	5.91×10^{-8}	2.16×10^{-6}
LJP005 BT20 3H-CGP-60474-1.11	7/45	7.34×10^{-8}	2.58×10^{-6}
LJP006 MCF10A 24H-CGP-60474-0.37	9/95	8.20×10^{-8}	2.83×10^{-6}
LJP005 A549 24H-CGP-60474-1.11	9/96	8.99×10^{-8}	3.05×10^{-6}
LJP008 PC3 24H-CGP-60474-1.11	10/128	9.74×10^{-8}	3.28×10^{-6}
LJP008 HCC515 24H-CGP-60474-10	9/97	9.84×10^{-8}	3.32×10^{-6}
LJP005 MCF7 24H-CGP-60474-0.37	9/97	9.84×10^{-8}	3.31×10^{-6}
LJP005 HA1E 24H-CGP-60474-10	8/72	1.26×10^{-7}	4.09×10^{-6}
LJP006 BT20 24H-CGP-60474-0.37	8/73	1.40×10^{-7}	4.46×10^{-6}
LJP006 BT20 24H-CGP-60474-0.04	9/104	1.80×10^{-7}	5.54×10^{-6}
LJP005 HT29 24H-CGP-60474-0.37	8/76	1.93×10^{-7}	5.91×10^{-6}
LJP008 HEPG2 24H-CGP-60474-3.33	9/105	1.96×10^{-7}	5.96×10^{-6}
LJP006 HS578T 24H-CGP-60474-0.37	9/106	2.12×10^{-7}	6.36×10^{-6}
LJP007 HT29 24H-CGP-60474-10	9/106	2.12×10^{-7}	6.36×10^{-6}
LJP008 MCF7 24H-CGP-60474-0.12	9/109	2.70×10^{-7}	7.74×10^{-6}
LJP005 MCF10A 3H-CGP-60474-10	9/109	2.70×10^{-7}	7.73×10^{-6}
LJP006 MCF10A 3H-CGP-60474-0.04	10/143	2.76×10^{-7}	7.86×10^{-6}
LJP007 HT29 24H-CGP-60474-0.04	8/80	2.89×10^{-7}	8.16×10^{-6}
LJP007 A549 24H-CGP-60474-0.04	6/34	2.97×10^{-7}	8.35×10^{-6}
LJP006 LNCAP 3H-CGP-60474-0.12	11/186	3.76×10^{-7}	1.02×10^{-5}
LJP006 HCC515 24H-CGP-60474-10	9/115	4.27×10^{-7}	1.13×10^{-5}
LJP005 MCF10A 3H-CGP-60474-1.11	9/116	4.60×10^{-7}	1.20×10^{-5}
LJP008 A549 24H-CGP-60474-1.11	8/85	4.63×10^{-7}	1.21×10^{-5}
LJP008 HEPG2 24H-CGP-60474-1.11	8/85	4.63×10^{-7}	1.21×10^{-5}
LJP008 HCC515 24H-CGP-60474-1.11	8/85	4.63×10^{-7}	1.20×10^{-5}
LJP006 HME1 3H-CGP-60474-0.12	11/192	5.16×10^{-7}	1.31×10^{-5}
LJP008 HCC515 24H-CGP-60474-3.33	9/118	5.32×10^{-7}	1.35×10^{-5}
LJP005 HA1E 24H-CGP-60474-1.11	8/87	5.54×10^{-7}	1.40×10^{-5}
LJP005 MDAMB231 3H-CGP-60474-1.11	8/87	5.54×10^{-7}	1.39×10^{-5}
LJP006 BT20 24H-CGP-60474-0.12	9/121	6.59×10^{-7}	1.62×10^{-5}
LJP009 HCC515 24H-CGP-60474-3.33	8/89	6.61×10^{-7}	1.62×10^{-5}
LJP008 PC3 24H-CGP-60474-0.37	9/122	7.06×10^{-7}	1.72×10^{-5}
LJP006 MCF7 24H-CGP-60474-0.04	7/62	7.08×10^{-7}	1.71×10^{-5}
LJP008 HA1E 24H-CGP-60474-0.37	8/90	7.20×10^{-7}	1.74×10^{-5}
LJP006 MDAMB231 24H-CGP-60474-10	8/90	7.20×10^{-7}	1.73×10^{-5}
LJP005 BT20 3H-CGP-60474-3.33	8/91	7.84×10^{-7}	1.88×10^{-5}
LJP006 HME1 24H-CGP-60474-10	8/91	7.84×10^{-7}	1.88×10^{-5}
LJP006 SKBR3 3H-CGP-60474-0.37	8/91	7.84×10^{-7}	1.88×10^{-5}
LJP008 A375 24H-CGP-60474-1.11	8/92	8.53×10^{-7}	2.02×10^{-5}
LJP009 HCC515 24H-CGP-60474-0.12	8/93	9.27×10^{-7}	2.18×10^{-5}
LJP007 A375 24H-CGP-60474-3.33	8/93	9.27×10^{-7}	2.17×10^{-5}
LJP006 HT29 24H-CGP-60474-10	8/94	1.01×10^{-6}	2.32×10^{-5}
LJP005 MCF7 24H-CGP-60474-1.11	8/96	1.18×10^{-6}	2.67×10^{-5}
LJP006 LNCAP 3H-CGP-60474-1.11	8/97	1.28×10^{-6}	2.85×10^{-5}
LJP006 HA1E 24H-CGP-60474-3.33	7/68	1.34×10^{-6}	2.95×10^{-5}

S17 Table: (Continued)

LJP006 MDAMB231 3H-CGP-60474-3.33	9/132	1.37×10^{-6}	3.01×10^{-5}
LJP007 MCF7 24H-CGP-60474-0.12	9/133	1.46×10^{-6}	3.19×10^{-5}
LJP005 HS578T 3H-CGP-60474-3.33	10/173	1.59×10^{-6}	3.43×10^{-5}
LJP007 HA1E 24H-CGP-60474-10	8/101	1.74×10^{-6}	3.68×10^{-5}
LJP009 A375 24H-CGP-60474-0.04	8/102	1.88×10^{-6}	3.92×10^{-5}
LJP006 HME1 24H-CGP-60474-1.11	7/72	1.98×10^{-6}	4.09×10^{-5}
LJP008 A375 24H-CGP-60474-0.04	9/138	1.99×10^{-6}	4.09×10^{-5}
LJP005 PC3 24H-CGP-60474-0.12	8/103	2.02×10^{-6}	4.16×10^{-5}
LJP007 A375 24H-CGP-60474-0.04	8/105	2.34×10^{-6}	4.72×10^{-5}
LJP005 HA1E 24H-CGP-60474-3.33	7/74	2.39×10^{-6}	4.79×10^{-5}
LJP005 HT29 24H-CGP-60474-1.11	8/106	2.51×10^{-6}	4.99×10^{-5}
LJP006 HCC515 24H-CGP-60474-0.37	8/106	2.51×10^{-6}	4.99×10^{-5}
LJP005 MCF7 3H-CGP-60474-10	8/107	2.69×10^{-6}	5.30×10^{-5}
LJP008 HA1E 24H-CGP-60474-3.33	7/76	2.86×10^{-6}	5.57×10^{-5}
LJP009 A549 24H-CGP-60474-0.37	7/76	2.86×10^{-6}	5.57×10^{-5}
LJP005 HCC515 24H-CGP-60474-0.37	8/108	2.89×10^{-6}	5.60×10^{-5}
LJP005 MCF10A 24H-CGP-60474-0.12	7/77	3.13×10^{-6}	6.01×10^{-5}
LJP008 A549 24H-CGP-60474-10	8/110	3.32×10^{-6}	6.33×10^{-5}
LJP005 SKBR3 3H-CGP-60474-10	7/78	3.41×10^{-6}	6.48×10^{-5}
LJP006 HS578T 24H-CGP-60474-10	7/78	3.41×10^{-6}	6.46×10^{-5}
LJP006 MCF7 24H-CGP-60474-3.33	5/29	3.43×10^{-6}	6.46×10^{-5}
LJP007 MCF7 24H-CGP-60474-0.04	10/189	3.53×10^{-6}	6.65×10^{-5}
LJP005 MCF7 3H-CGP-60474-1.11	8/111	3.55×10^{-6}	6.68×10^{-5}
LJP006 LNCAP 24H-CGP-60474-3.33	8/111	3.55×10^{-6}	6.67×10^{-5}
LJP006 HS578T 24H-CGP-60474-0.04	7/79	3.72×10^{-6}	6.95×10^{-5}
LJP009 HEPG2 24H-CGP-60474-1.11	8/112	3.80×10^{-6}	7.08×10^{-5}
LJP008 A549 24H-CGP-60474-0.37	7/80	4.05×10^{-6}	7.50×10^{-5}
LJP006 MCF10A 3H-CGP-60474-0.12	9/151	4.18×10^{-6}	7.69×10^{-5}
LJP006 HS578T 3H-CGP-60474-0.04	8/115	4.63×10^{-6}	8.43×10^{-5}
LJP006 MCF10A 3H-CGP-60474-1.11	8/115	4.63×10^{-6}	8.42×10^{-5}
LJP005 A549 24H-CGP-60474-3.33	8/116	4.94×10^{-6}	8.93×10^{-5}
LJP006 HS578T 24H-CGP-60474-0.12	7/83	5.18×10^{-6}	9.27×10^{-5}
LJP009 HEPG2 24H-CGP-60474-0.12	8/117	5.26×10^{-6}	9.39×10^{-5}
LJP005 HA1E 24H-CGP-60474-0.04	8/118	5.61×10^{-6}	9.93×10^{-5}
LJP008 PC3 24H-CGP-60474-0.12	8/118	5.61×10^{-6}	9.92×10^{-5}
LJP005 SKBR3 3H-CGP-60474-0.37	7/87	7.10×10^{-6}	1.21×10^{-4}
LJP006 HCC515 24H-CGP-60474-1.11	7/87	7.10×10^{-6}	1.21×10^{-4}
LJP007 HT29 24H-CGP-60474-3.33	7/87	7.10×10^{-6}	1.20×10^{-4}
LJP006 HME1 3H-CGP-60474-0.37	9/162	7.41×10^{-6}	1.26×10^{-4}
LJP005 HEPG2 24H-CGP-60474-1.11	6/59	8.42×10^{-6}	1.40×10^{-4}
LJP005 MDAMB231 3H-CGP-60474-3.33	8/125	8.59×10^{-6}	1.42×10^{-4}
LJP008 HCC515 24H-CGP-60474-0.04	7/91	9.57×10^{-6}	1.56×10^{-4}
LJP006 HME1 24H-CGP-60474-0.12	7/91	9.57×10^{-6}	1.56×10^{-4}
LJP006 HS578T 3H-CGP-60474-1.11	8/127	9.66×10^{-6}	1.57×10^{-4}
LJP006 MDAMB231 24H-CGP-60474-3.33	7/92	1.03×10^{-5}	1.66×10^{-4}
LJP007 HCC515 24H-CGP-60474-0.37	7/92	1.03×10^{-5}	1.65×10^{-4}
LJP007 HT29 24H-CGP-60474-0.12	7/92	1.03×10^{-5}	1.65×10^{-4}
LJP007 HEPG2 24H-CGP-60474-0.04	6/62	1.13×10^{-5}	1.79×10^{-4}
LJP008 MCF7 24H-CGP-60474-0.04	7/94	1.19×10^{-5}	1.88×10^{-4}
LJP006 BT20 3H-CGP-60474-0.04	5/37	1.19×10^{-5}	1.88×10^{-4}
LJP009 HEPG2 24H-CGP-60474-0.04	8/133	1.35×10^{-5}	2.11×10^{-4}
LJP005 HEPG2 24H-CGP-60474-3.33	6/64	1.36×10^{-5}	2.11×10^{-4}
LJP008 HT29 24H-CGP-60474-0.37	6/64	1.36×10^{-5}	2.11×10^{-4}
LJP009 HEPG2 24H-CGP-60474-3.33	7/97	1.46×10^{-5}	2.24×10^{-4}
LJP005 MCF10A 24H-CGP-60474-0.04	6/65	1.48×10^{-5}	2.27×10^{-4}
LJP009 HT29 24H-CGP-60474-1.11	6/66	1.62×10^{-5}	2.45×10^{-4}
LJP006 HS578T 3H-CGP-60474-3.33	7/99	1.67×10^{-5}	2.49×10^{-4}

S17 Table: (Continued)

LJP009 HT29 24H-CGP-60474-0.04	6/67	1.77×10^{-5}	2.62×10^{-4}
LJP006 A549 24H-CGP-60474-0.37	6/67	1.77×10^{-5}	2.62×10^{-4}
LJP006 MDAMB231 3H-CGP-60474-1.11	8/138	1.77×10^{-5}	2.61×10^{-4}
LJP007 MCF7 24H-CGP-60474-3.33	7/101	1.90×10^{-5}	2.79×10^{-4}
LJP006 HME1 3H-CGP-60474-3.33	8/141	2.07×10^{-5}	3.01×10^{-4}
LJP006 MDAMB231 3H-CGP-60474-0.04	6/69	2.10×10^{-5}	3.04×10^{-4}
LJP005 HS578T 24H-CGP-60474-0.37	7/103	2.16×10^{-5}	3.13×10^{-4}
LJP009 MCF7 24H-CGP-60474-0.37	8/142	2.18×10^{-5}	3.15×10^{-4}
LJP005 MCF10A 3H-CGP-60474-0.12	7/104	2.30×10^{-5}	3.28×10^{-4}
LJP006 MDAMB231 3H-CGP-60474-0.37	7/105	2.45×10^{-5}	3.49×10^{-4}
LJP008 PC3 24H-CGP-60474-3.33	8/145	2.54×10^{-5}	3.59×10^{-4}
LJP007 HA1E 24H-CGP-60474-1.11	6/72	2.68×10^{-5}	3.76×10^{-4}
LJP006 HS578T 3H-CGP-60474-0.12	7/107	2.76×10^{-5}	3.88×10^{-4}
LJP006 HME1 24H-CGP-60474-3.33	6/73	2.90×10^{-5}	4.01×10^{-4}
LJP008 A549 24H-CGP-60474-3.33	6/74	3.13×10^{-5}	4.31×10^{-4}
LJP006 LNCAP 3H-CGP-60474-3.33	7/110	3.31×10^{-5}	4.51×10^{-4}
LJP007 A375 24H-CGP-60474-10	7/110	3.31×10^{-5}	4.50×10^{-4}
LJP006 MCF7 24H-CGP-60474-10	6/75	3.38×10^{-5}	4.58×10^{-4}
LJP006 HA1E 24H-CGP-60474-0.37	6/76	3.65×10^{-5}	4.88×10^{-4}
LJP007 PC3 24H-CGP-60474-0.04	6/77	3.93×10^{-5}	5.18×10^{-4}
LJP006 A549 24H-CGP-60474-10	6/79	4.54×10^{-5}	5.85×10^{-4}
LJP005 MCF10A 24H-CGP-60474-0.37	6/80	4.88×10^{-5}	6.21×10^{-4}
LJP006 MCF7 24H-CGP-60474-1.11	6/80	4.88×10^{-5}	6.20×10^{-4}
LJP008 A375 24H-CGP-60474-0.37	6/81	5.24×10^{-5}	6.62×10^{-4}
LJP008 A375 24H-CGP-60474-0.12	6/82	5.61×10^{-5}	7.01×10^{-4}
LJP005 HEPG2 24H-CGP-60474-0.37	6/82	5.61×10^{-5}	7.01×10^{-4}
LJP005 MDAMB231 3H-CGP-60474-0.12	6/82	5.61×10^{-5}	7.00×10^{-4}
LJP007 HCC515 24H-CGP-60474-1.11	6/82	5.61×10^{-5}	6.98×10^{-4}
LJP006 MCF7 3H-CGP-60474-3.33	7/120	5.77×10^{-5}	7.17×10^{-4}
LJP007 HCC515 24H-CGP-60474-3.33	6/83	6.01×10^{-5}	7.41×10^{-4}
LJP009 A375 24H-CGP-60474-0.37	7/121	6.09×10^{-5}	7.50×10^{-4}
LJP005 MDAMB231 24H-CGP-60474-0.12	4/27	6.45×10^{-5}	7.86×10^{-4}
LJP005 MDAMB231 3H-CGP-60474-10	7/123	6.75×10^{-5}	8.17×10^{-4}
LJP008 HT29 24H-CGP-60474-3.33	6/85	6.87×10^{-5}	8.29×10^{-4}
LJP006 MDAMB231 3H-CGP-60474-0.12	7/125	7.48×10^{-5}	8.86×10^{-4}
LJP006 HS578T 24H-CGP-60474-1.11	6/87	7.83×10^{-5}	9.18×10^{-4}
LJP007 HCC515 24H-CGP-60474-0.12	6/87	7.83×10^{-5}	9.17×10^{-4}
LJP005 MDAMB231 3H-CGP-60474-0.37	7/129	9.12×10^{-5}	1.04×10^{-3}
LJP006 LNCAP 24H-CGP-60474-1.11	7/129	9.12×10^{-5}	1.04×10^{-3}
LJP005 HS578T 3H-CGP-60474-0.04	5/56	9.23×10^{-5}	1.05×10^{-3}
LJP006 LNCAP 24H-CGP-60474-0.12	7/130	9.58×10^{-5}	1.08×10^{-3}
LJP006 PC3 24H-CGP-60474-10	5/57	1.01×10^{-4}	1.13×10^{-3}
LJP005 PC3 24H-CGP-60474-3.33	6/92	1.07×10^{-4}	1.19×10^{-3}
LJP006 HA1E 24H-CGP-60474-10	5/60	1.29×10^{-4}	1.39×10^{-3}
LJP009 HCC515 24H-CGP-60474-10	6/96	1.35×10^{-4}	1.46×10^{-3}
LJP005 HA1E 24H-CGP-60474-0.12	6/96	1.35×10^{-4}	1.46×10^{-3}
LJP007 HCC515 24H-CGP-60474-0.04	6/96	1.35×10^{-4}	1.45×10^{-3}
LJP008 HA1E 24H-CGP-60474-1.11	6/97	1.43×10^{-4}	1.53×10^{-3}
LJP005 SKBR3 24H-CGP-60474-0.12	6/97	1.43×10^{-4}	1.53×10^{-3}
LJP006 HME1 24H-CGP-60474-0.37	6/97	1.43×10^{-4}	1.53×10^{-3}
LJP007 A549 24H-CGP-60474-3.33	4/33	1.45×10^{-4}	1.53×10^{-3}
LJP005 BT20 3H-CGP-60474-0.04	4/34	1.63×10^{-4}	1.70×10^{-3}
LJP007 HA1E 24H-CGP-60474-0.37	6/100	1.69×10^{-4}	1.75×10^{-3}
LJP006 LNCAP 24H-CGP-60474-0.37	7/144	1.81×10^{-4}	1.86×10^{-3}
LJP006 BT20 3H-CGP-60474-0.12	4/35	1.83×10^{-4}	1.87×10^{-3}
LJP009 HA1E 24H-CGP-60474-3.33	6/107	2.45×10^{-4}	2.41×10^{-3}
LJP006 MCF10A 24H-CGP-60474-0.04	5/69	2.49×10^{-4}	2.45×10^{-3}

S17 Table: (Continued)

LJP006 A375 24H-CGP-60474-10	6/108	2.58×10^{-4}	2.51×10^{-3}
LJP005 MCF10A 3H-CGP-60474-0.37	7/154	2.73×10^{-4}	2.65×10^{-3}
LJP008 MCF7 24H-CGP-60474-10	5/71	2.85×10^{-4}	2.73×10^{-3}
LJP009 A375 24H-CGP-60474-1.11	6/112	3.14×10^{-4}	2.96×10^{-3}
LJP005 MCF10A 3H-CGP-60474-3.33	5/73	3.25×10^{-4}	3.06×10^{-3}
LJP006 HEPG2 24H-CGP-60474-0.37	5/73	3.25×10^{-4}	3.06×10^{-3}
LJP006 PC3 24H-CGP-60474-3.33	4/41	3.40×10^{-4}	3.16×10^{-3}
LJP009 HA1E 24H-CGP-60474-1.11	5/76	3.92×10^{-4}	3.57×10^{-3}
LJP006 HA1E 24H-CGP-60474-0.04	5/76	3.92×10^{-4}	3.57×10^{-3}
LJP008 HEPG2 24H-CGP-60474-0.37	6/118	4.15×10^{-4}	3.73×10^{-3}
LJP006 LNCAP 3H-CGP-60474-0.37	6/119	4.34×10^{-4}	3.88×10^{-3}
LJP006 SKBR3 3H-CGP-60474-10	6/119	4.34×10^{-4}	3.88×10^{-3}
LJP005 HCC515 24H-CGP-60474-0.12	5/78	4.42×10^{-4}	3.94×10^{-3}
LJP006 MCF7 3H-CGP-60474-0.37	7/167	4.46×10^{-4}	3.97×10^{-3}
LJP006 HT29 24H-CGP-60474-1.11	5/80	4.97×10^{-4}	4.34×10^{-3}
LJP006 MDAMB231 3H-CGP-60474-10	7/170	4.97×10^{-4}	4.34×10^{-3}
LJP005 HS578T 3H-CGP-60474-0.12	5/81	5.26×10^{-4}	4.58×10^{-3}
LJP007 A375 24H-CGP-60474-1.11	5/81	5.26×10^{-4}	4.57×10^{-3}
LJP005 A375 24H-CGP-60474-1.11	5/82	5.56×10^{-4}	4.78×10^{-3}
LJP006 MCF7 3H-CGP-60474-1.11	6/125	5.64×10^{-4}	4.83×10^{-3}
LJP006 BT20 3H-CGP-60474-10	4/48	6.26×10^{-4}	5.26×10^{-3}
LJP005 HA1E 24H-CGP-60474-0.37	5/86	6.92×10^{-4}	5.73×10^{-3}
LJP008 A375 24H-CGP-60474-10	5/88	7.68×10^{-4}	6.27×10^{-3}
LJP008 A549 24H-CGP-60474-0.04	6/133	7.82×10^{-4}	6.37×10^{-3}
LJP009 MCF7 24H-CGP-60474-10	6/134	8.13×10^{-4}	6.57×10^{-3}
LJP005 MCF7 3H-CGP-60474-3.33	5/90	8.51×10^{-4}	6.80×10^{-3}
LJP009 MCF7 24H-CGP-60474-1.11	6/136	8.78×10^{-4}	7.01×10^{-3}
LJP005 SKBR3 3H-CGP-60474-0.12	4/54	9.79×10^{-4}	7.70×10^{-3}
LJP006 LNCAP 3H-CGP-60474-0.04	5/93	9.86×10^{-4}	7.71×10^{-3}
LJP006 BT20 3H-CGP-60474-3.33	4/56	1.12×10^{-3}	8.61×10^{-3}
LJP006 HA1E 24H-CGP-60474-0.12	5/97	1.19×10^{-3}	9.08×10^{-3}
LJP006 HT29 24H-CGP-60474-0.37	5/97	1.19×10^{-3}	9.07×10^{-3}
LJP005 A375 24H-CGP-60474-0.04	4/57	1.20×10^{-3}	9.12×10^{-3}
LJP005 BT20 3H-CGP-60474-10	4/57	1.20×10^{-3}	9.11×10^{-3}
LJP008 PC3 24H-CGP-60474-0.04	6/145	1.22×10^{-3}	9.23×10^{-3}
LJP007 HEPG2 24H-CGP-60474-0.12	4/60	1.45×10^{-3}	1.07×10^{-2}
LJP007 MCF7 24H-CGP-60474-10	5/103	1.56×10^{-3}	1.13×10^{-2}
LJP005 MCF7 3H-CGP-60474-0.37	6/152	1.56×10^{-3}	1.13×10^{-2}
LJP006 HA1E 24H-CGP-60474-1.11	4/62	1.64×10^{-3}	1.18×10^{-2}
LJP007 A549 24H-CGP-60474-10	3/29	1.66×10^{-3}	1.19×10^{-2}
LJP005 A549 24H-CGP-60474-0.04	4/64	1.85×10^{-3}	1.30×10^{-2}
LJP006 A375 24H-CGP-60474-1.11	4/64	1.85×10^{-3}	1.30×10^{-2}
LJP006 MCF7 3H-CGP-60474-0.04	4/64	1.85×10^{-3}	1.30×10^{-2}
LJP009 MCF7 24H-CGP-60474-0.12	6/158	1.89×10^{-3}	1.33×10^{-2}
LJP006 PC3 24H-CGP-60474-1.11	4/66	2.07×10^{-3}	1.43×10^{-2}
LJP009 PC3 24H-CGP-60474-10	5/110	2.08×10^{-3}	1.43×10^{-2}
LJP005 HS578T 24H-CGP-60474-0.04	4/67	2.19×10^{-3}	1.50×10^{-2}
LJP007 HA1E 24H-CGP-60474-3.33	4/70	2.57×10^{-3}	1.71×10^{-2}
LJP008 A375 24H-CGP-60474-3.33	4/72	2.84×10^{-3}	1.87×10^{-2}
LJP006 MCF7 3H-CGP-60474-10	5/119	2.93×10^{-3}	1.90×10^{-2}
LJP006 LNCAP 24H-CGP-60474-10	5/122	3.26×10^{-3}	2.08×10^{-2}
LJP006 MCF7 24H-CGP-60474-0.37	4/76	3.46×10^{-3}	2.18×10^{-2}
LJP005 HS578T 3H-CGP-60474-10	5/125	3.62×10^{-3}	2.27×10^{-2}
LJP005 HT29 24H-CGP-60474-0.12	4/78	3.80×10^{-3}	2.35×10^{-2}
LJP008 HT29 24H-CGP-60474-0.04	4/79	3.98×10^{-3}	2.43×10^{-2}
LJP006 MDAMB231 24H-CGP-60474-0.12	4/79	3.98×10^{-3}	2.43×10^{-2}
LJP007 HA1E 24H-CGP-60474-0.12	5/138	5.49×10^{-3}	3.17×10^{-2}

S17 Table: (Continued)

LJP005 MDAMB231 24H-CGP-60474-0.37	3/44	5.51×10^{-3}	3.16×10^{-2}
LJP007 PC3 24H-CGP-60474-3.33	4/87	5.60×10^{-3}	3.20×10^{-2}
LJP007 MCF7 24H-CGP-60474-1.11	5/139	5.66×10^{-3}	3.22×10^{-2}
LJP005 A375 24H-CGP-60474-3.33	4/88	5.83×10^{-3}	3.31×10^{-2}
LJP007 PC3 24H-CGP-60474-10	4/88	5.83×10^{-3}	3.31×10^{-2}
LJP006 BT20 3H-CGP-60474-0.37	3/49	7.44×10^{-3}	3.99×10^{-2}
LJP007 MCF7 24H-CGP-60474-0.37	5/149	7.55×10^{-3}	4.04×10^{-2}
LJP009 MCF7 24H-CGP-60474-3.33	5/151	7.98×10^{-3}	4.23×10^{-2}
LJP008 MCF7 24H-CGP-60474-1.11	4/98	8.49×10^{-3}	4.44×10^{-2}
LJP005 SKBR3 3H-CGP-60474-3.33	4/98	8.49×10^{-3}	4.43×10^{-2}
LJP005 HS578T 3H-CGP-60474-1.11	5/159	9.84×10^{-3}	5.00×10^{-2}
