# Supplementary material for: A new advanced in silico drug discovery method for novel coronavirus (SARS-CoV-2) with tensor decomposition-based unsupervised feature extraction
Source: PLoS One. 2020 Sep 11;15(9):e0238907. doi: 10.1371/journal.pone.0238907 (PMC7485840; doi:10.1371/journal.pone.0238907)
Supplement: S18 Table — Alvocidib significantly affects the expression of the selected 163 genes as evident in the “LINCS L1000 Chem Pert up” category in Enrichr. The last number after the—is dose density. (PDF) [file pone.0238907.s018.pdf]

S18 Table: Alvocidib significantly affects the expression of the selected 163 genes as evident in the "LINCS L1000 Chem Pert up" category in Enrichr. The last number after the - is dose density.

Term	Overlap	P-value	Adjusted P-value
LINCS L1000 Chem Pert up			
LJP006 MCF7 3H-alvocidib-10	9/87	3.79×10^{-8}	1.57×10^{-6}
LJP006 SKBR3 24H-alvocidib-1.11	9/116	4.60×10^{-7}	1.29×10^{-5}
LJP006 HME1 3H-alvocidib-0.12	7/82	4.78×10^{-6}	9.07×10^{-5}
LJP006 HS578T 3H-alvocidib-0.37	6/61	1.02×10^{-5}	1.69×10^{-4}
LJP006 BT20 24H-alvocidib-3.33	7/108	2.94×10^{-5}	4.11×10^{-4}
LJP006 HME1 3H-alvocidib-1.11	5/56	9.23×10^{-5}	1.07×10^{-3}
LJP006 BT20 24H-alvocidib-0.04	5/57	1.01×10^{-4}	1.15×10^{-3}
LJP006 SKBR3 3H-alvocidib-0.37	5/57	1.01×10^{-4}	1.15×10^{-3}
LJP006 BT20 24H-alvocidib-1.11	6/98	1.52×10^{-4}	1.62×10^{-3}
LJP006 MCF10A 24H-alvocidib-0.04	6/106	2.33×10^{-4}	2.31×10^{-3}
LJP006 MCF10A 24H-alvocidib-10	7/150	2.33×10^{-4}	2.31×10^{-3}
LJP006 SKBR3 3H-alvocidib-0.12	5/71	2.85×10^{-4}	2.74×10^{-3}
LJP006 BT20 24H-alvocidib-10	5/78	4.42×10^{-4}	3.96×10^{-3}
LJP006 LNCAP 24H-alvocidib-3.33	5/80	4.97×10^{-4}	4.36×10^{-3}
LJP006 HS578T 3H-alvocidib-0.12	5/85	6.56×10^{-4}	5.52×10^{-3}
LJP006 SKBR3 24H-alvocidib-3.33	6/129	6.66×10^{-4}	5.60×10^{-3}
LJP006 HME1 3H-alvocidib-0.37	4/51	7.89×10^{-4}	6.42×10^{-3}
LJP006 HEPG2 24H-alvocidib-10	5/91	8.94×10^{-4}	7.15×10^{-3}
LJP006 LNCAP 24H-alvocidib-0.04	6/140	1.02×10^{-3}	7.97×10^{-3}
LJP006 MCF10A 24H-alvocidib-3.33	6/146	1.27×10^{-3}	9.48×10^{-3}
LJP006 MCF7 3H-alvocidib-0.37	4/60	1.45×10^{-3}	1.06×10^{-2}
LJP006 PC3 24H-alvocidib-10	6/152	1.56×10^{-3}	1.12×10^{-2}
LJP006 HS578T 3H-alvocidib-10	4/63	1.74×10^{-3}	1.23×10^{-2}
LJP006 LNCAP 3H-alvocidib-0.37	4/63	1.74×10^{-3}	1.23×10^{-2}
LJP006 BT20 24H-alvocidib-0.12	5/106	1.77×10^{-3}	1.24×10^{-2}
LJP006 HS578T 3H-alvocidib-0.04	4/64	1.85×10^{-3}	1.28×10^{-2}
LJP006 MCF7 3H-alvocidib-3.33	4/67	2.19×10^{-3}	1.48×10^{-2}
LJP006 MDAMB231 3H-alvocidib-1.11	4/67	2.19×10^{-3}	1.48×10^{-2}
LJP006 SKBR3 3H-alvocidib-1.11	4/67	2.19×10^{-3}	1.48×10^{-2}
LJP006 HCC515 24H-alvocidib-10	7/221	2.27×10^{-3}	1.53×10^{-2}
LJP006 MCF7 3H-alvocidib-0.04	3/37	3.37×10^{-3}	2.11×10^{-2}
LJP006 A549 24H-alvocidib-10	6/180	3.62×10^{-3}	2.24×10^{-2}
LJP006 MCF7 24H-alvocidib-0.04	3/47	6.63×10^{-3}	3.57×10^{-2}
LJP006 MCF7 3H-alvocidib-0.12	3/47	6.63×10^{-3}	3.56×10^{-2}
LJP006 MCF10A 24H-alvocidib-0.12	5/146	6.94×10^{-3}	3.71×10^{-2}
LJP006 SKBR3 24H-alvocidib-0.37	4/93	7.08×10^{-3}	3.76×10^{-2}
LJP006 HA1E 24H-alvocidib-1.11	6/218	9.03×10^{-3}	4.56×10^{-2}
LJP006 MCF10A 3H-alvocidib-3.33	3/54	9.73×10^{-3}	4.82×10^{-2}
