# Supplementary material for: A new advanced in silico drug discovery method for novel coronavirus (SARS-CoV-2) with tensor decomposition-based unsupervised feature extraction
Source: PLoS One. 2020 Sep 11;15(9):e0238907. doi: 10.1371/journal.pone.0238907 (PMC7485840; doi:10.1371/journal.pone.0238907)
Supplement: S19 Table — Alvocidib significantly affects the expression of the selected 163 genes as evident in the “LINCS L1000 Chem Pert down” category in Enrichr. The last number after the—is dose density. (PDF) [file pone.0238907.s019.pdf]

S19 Table: Alvocidib significantly affects the expression of the selected 163 genes as evident in the "LINCS L1000 Chem Pert down" category in Enrichr. The last number after the - is dose density.

Term	Overlap	P-value	Adjusted P-value
LINCS L1000 Chem Pert down			
LJP006 SKBR3 24H-alvocidib-0.12	18/111	1.20×10^{-18}	6.61×10^{-15}
LJP006 MCF10A 24H-alvocidib-3.33	12/92	1.18×10^{-11}	1.78×10^{-9}
LJP006 SKBR3 24H-alvocidib-0.37	12/92	1.18×10^{-11}	1.78×10^{-9}
LJP006 SKBR3 24H-alvocidib-10	12/95	1.74×10^{-11}	2.47×10^{-9}
LJP006 BT20 24H-alvocidib-0.12	13/134	7.29×10^{-11}	8.13×10^{-9}
LJP006 HME1 3H-alvocidib-1.11	14/166	8.78×10^{-11}	9.35×10^{-9}
LJP006 MCF10A 24H-alvocidib-0.12	11/89	1.58×10^{-10}	1.52×10^{-8}
LJP006 SKBR3 24H-alvocidib-3.33	12/115	1.71×10^{-10}	1.61×10^{-8}
LJP006 HME1 3H-alvocidib-3.33	14/194	6.96×10^{-10}	5.20×10^{-8}
LJP006 MCF10A 3H-alvocidib-0.37	12/143	2.16×10^{-9}	1.33×10^{-7}
LJP006 HCC515 24H-alvocidib-10	11/118	3.39×10^{-9}	1.94×10^{-7}
LJP006 HME1 3H-alvocidib-0.37	13/194	7.11×10^{-9}	3.61×10^{-7}
LJP006 HS578T 3H-alvocidib-10	11/143	2.57×10^{-8}	1.04×10^{-6}
LJP006 HA1E 24H-alvocidib-0.04	12/188	4.72×10^{-8}	1.78×10^{-6}
LJP006 SKBR3 24H-alvocidib-1.11	9/93	6.81×10^{-8}	2.41×10^{-6}
LJP006 LNCAP 3H-alvocidib-10	10/131	1.21×10^{-7}	3.94×10^{-6}
LJP006 SKBR3 3H-alvocidib-1.11	9/104	1.80×10^{-7}	5.53×10^{-6}
LJP006 MCF10A 3H-alvocidib-10	10/146	3.35×10^{-7}	9.25×10^{-6}
LJP006 MCF7 24H-alvocidib-3.33	6/35	3.57×10^{-7}	9.71×10^{-6}
LJP006 HME1 3H-alvocidib-10	9/114	3.97×10^{-7}	1.06×10^{-5}
LJP006 BT20 24H-alvocidib-3.33	9/115	4.27×10^{-7}	1.13×10^{-5}
LJP006 SKBR3 3H-alvocidib-0.37	8/86	5.07×10^{-7}	1.29×10^{-5}
LJP006 A549 24H-alvocidib-10	8/94	1.01×10^{-6}	2.33×10^{-5}
LJP006 BT20 24H-alvocidib-10	8/94	1.01×10^{-6}	2.32×10^{-5}
LJP006 SKBR3 3H-alvocidib-10	8/97	1.28×10^{-6}	2.85×10^{-5}
LJP006 HME1 24H-alvocidib-0.12	8/99	1.50×10^{-6}	3.23×10^{-5}
LJP006 LNCAP 24H-alvocidib-10	8/101	1.74×10^{-6}	3.68×10^{-5}
LJP006 MCF10A 24H-alvocidib-10	7/71	1.80×10^{-6}	3.77×10^{-5}
LJP006 HA1E 24H-alvocidib-10	7/72	1.98×10^{-6}	4.09×10^{-5}
LJP006 MCF7 3H-alvocidib-1.11	7/74	2.39×10^{-6}	4.78×10^{-5}
LJP006 HME1 3H-alvocidib-0.12	9/146	3.17×10^{-6}	6.04×10^{-5}
LJP006 MDAMB231 24H-alvocidib-1.11	7/82	4.78×10^{-6}	8.68×10^{-5}
LJP006 MCF7 24H-alvocidib-0.37	5/31	4.84×10^{-6}	8.76×10^{-5}
LJP006 LNCAP 24H-alvocidib-1.11	8/116	4.94×10^{-6}	8.92×10^{-5}
LJP006 HA1E 24H-alvocidib-0.12	7/83	5.18×10^{-6}	9.29×10^{-5}
LJP006 HS578T 24H-alvocidib-3.33	7/83	5.18×10^{-6}	9.28×10^{-5}
LJP006 MCF10A 3H-alvocidib-0.12	8/120	6.35×10^{-6}	1.10×10^{-4}
LJP006 MCF10A 3H-alvocidib-1.11	8/122	7.18×10^{-6}	1.22×10^{-4}
LJP006 LNCAP 24H-alvocidib-3.33	6/58	7.62×10^{-6}	1.29×10^{-4}
LJP006 HA1E 24H-alvocidib-0.37	7/89	8.26×10^{-6}	1.37×10^{-4}
LJP006 MDAMB231 24H-alvocidib-10	6/61	1.02×10^{-5}	1.65×10^{-4}
LJP006 SKBR3 24H-alvocidib-0.04	7/99	1.67×10^{-5}	2.49×10^{-4}
LJP006 HT29 24H-alvocidib-10	6/67	1.77×10^{-5}	2.61×10^{-4}
LJP006 HME1 24H-alvocidib-0.37	7/103	2.16×10^{-5}	3.12×10^{-4}
LJP006 HS578T 3H-alvocidib-1.11	7/105	2.45×10^{-5}	3.49×10^{-4}
LJP006 HEPG2 24H-alvocidib-10	6/75	3.38×10^{-5}	4.58×10^{-4}
LJP006 LNCAP 3H-alvocidib-0.37	7/111	3.50×10^{-5}	4.73×10^{-4}
LJP006 LNCAP 24H-alvocidib-0.12	7/112	3.71×10^{-5}	4.94×10^{-4}
LJP006 MDAMB231 3H-alvocidib-3.33	7/114	4.16×10^{-5}	5.44×10^{-4}
LJP006 HS578T 3H-alvocidib-0.12	8/156	4.28×10^{-5}	5.56×10^{-4}
LJP006 MDAMB231 3H-alvocidib-0.12	8/156	4.28×10^{-5}	5.56×10^{-4}
LJP006 MDAMB231 3H-alvocidib-0.37	7/120	5.77×10^{-5}	7.17×10^{-4}
LJP006 MCF10A 3H-alvocidib-0.04	7/123	6.75×10^{-5}	8.16×10^{-4}
LJP006 BT20 24H-alvocidib-0.37	7/124	7.11×10^{-5}	8.51×10^{-4}

S19 Table: (Continued)

LJP006 HA1E 24H-alvocidib-3.33	6/86	7.33×10^{-5}	8.75×10^{-4}
LJP006 MCF7 3H-alvocidib-3.33	7/125	7.48×10^{-5}	8.86×10^{-4}
LJP006 HS578T 24H-alvocidib-0.37	6/89	8.89×10^{-5}	1.02×10^{-3}
LJP006 MCF7 24H-alvocidib-10	5/57	1.01×10^{-4}	1.13×10^{-3}
LJP006 HS578T 24H-alvocidib-1.11	6/91	1.01×10^{-4}	1.12×10^{-3}
LJP006 MCF7 3H-alvocidib-10	7/132	1.05×10^{-4}	1.18×10^{-3}
LJP006 BT20 3H-alvocidib-0.37	5/62	1.50×10^{-4}	1.59×10^{-3}
LJP006 LNCAP 24H-alvocidib-0.37	7/140	1.52×10^{-4}	1.60×10^{-3}
LJP006 MCF10A 3H-alvocidib-3.33	7/143	1.74×10^{-4}	1.79×10^{-3}
LJP006 HS578T 3H-alvocidib-3.33	7/148	2.14×10^{-4}	2.15×10^{-3}
LJP006 MDAMB231 3H-alvocidib-1.11	7/149	2.23×10^{-4}	2.23×10^{-3}
LJP006 MCF7 3H-alvocidib-0.37	6/108	2.58×10^{-4}	2.51×10^{-3}
LJP006 LNCAP 3H-alvocidib-1.11	6/112	3.14×10^{-4}	2.96×10^{-3}
LJP006 BT20 24H-alvocidib-0.04	5/73	3.25×10^{-4}	3.06×10^{-3}
LJP006 SKBR3 3H-alvocidib-0.12	6/113	3.29×10^{-4}	3.08×10^{-3}
LJP006 MCF7 3H-alvocidib-0.12	6/121	4.75×10^{-4}	4.18×10^{-3}
LJP006 MDAMB231 24H-alvocidib-3.33	5/82	5.56×10^{-4}	4.77×10^{-3}
LJP006 HME1 24H-alvocidib-3.33	5/83	5.88×10^{-4}	4.99×10^{-3}
LJP006 LNCAP 3H-alvocidib-0.12	6/127	6.13×10^{-4}	5.19×10^{-3}
LJP006 MDAMB231 3H-alvocidib-0.04	5/87	7.29×10^{-4}	6.01×10^{-3}
LJP006 LNCAP 24H-alvocidib-0.04	8/238	7.64×10^{-4}	6.24×10^{-3}
LJP006 HS578T 24H-alvocidib-0.04	4/51	7.89×10^{-4}	6.40×10^{-3}
LJP006 HS578T 24H-alvocidib-0.12	5/89	8.09×10^{-4}	6.54×10^{-3}
LJP006 MDAMB231 3H-alvocidib-10	6/139	9.84×10^{-4}	7.70×10^{-3}
LJP006 HS578T 3H-alvocidib-0.37	6/141	1.06×10^{-3}	8.19×10^{-3}
LJP006 BT20 24H-alvocidib-1.11	5/95	1.09×10^{-3}	8.35×10^{-3}
LJP006 HME1 3H-alvocidib-0.04	6/142	1.10×10^{-3}	8.44×10^{-3}
LJP006 SKBR3 3H-alvocidib-0.04	4/59	1.37×10^{-3}	1.01×10^{-2}
LJP006 HME1 24H-alvocidib-0.04	5/108	1.92×10^{-3}	1.35×10^{-2}
LJP006 A375 24H-alvocidib-10	5/109	2.00×10^{-3}	1.39×10^{-2}
LJP006 BT20 3H-alvocidib-10	3/40	4.21×10^{-3}	2.55×10^{-2}
LJP006 HS578T 24H-alvocidib-10	4/86	5.38×10^{-3}	3.10×10^{-2}
LJP006 HME1 24H-alvocidib-1.11	4/88	5.83×10^{-3}	3.31×10^{-2}
LJP006 HA1E 24H-alvocidib-1.11	4/89	6.07×10^{-3}	3.40×10^{-2}
LJP006 MDAMB231 24H-alvocidib-0.37	4/92	6.82×10^{-3}	3.73×10^{-2}
LJP006 MDAMB231 24H-alvocidib-0.04	3/48	7.03×10^{-3}	3.82×10^{-2}
LJP006 MCF7 24H-alvocidib-0.12	2/17	8.29×10^{-3}	4.36×10^{-2}
LJP006 HS578T 3H-alvocidib-0.04	4/99	8.79×10^{-3}	4.55×10^{-2}
LJP006 LNCAP 3H-alvocidib-3.33	5/155	8.87×10^{-3}	4.59×10^{-2}
LJP006 BT20 3H-alvocidib-0.12	3/53	9.24×10^{-3}	4.75×10^{-2}
