# Supplementary material for: A new advanced in silico drug discovery method for novel coronavirus (SARS-CoV-2) with tensor decomposition-based unsupervised feature extraction
Source: PLoS One. 2020 Sep 11;15(9):e0238907. doi: 10.1371/journal.pone.0238907 (PMC7485840; doi:10.1371/journal.pone.0238907)
Supplement: S20 Table — Mitoxantrone significantly affects the expression of the selected 163 genes as evident in the “LINCS L1000 Chem Pert up” category in Enrichr. The last number after the—is dose density. (PDF) [file pone.0238907.s020.pdf]

S20 Table: Mitoxantrone significantly affects the expression of the selected 163 genes as evident in the "LINCS L1000 Chem Pert up" category in Enrichr. The last number after the - is dose density.

Term	Overlap	P-value	Adjusted P-value
LINCS L1000 Chem Pert up			
LJP005 HEPG2 24H-mitoxantrone-0.37	19/142	5.43×10^{-18}	1.06×10^{-14}
LJP005 HEPG2 24H-mitoxantrone-0.12	14/110	3.03×10^{-13}	9.65×10^{-11}
LJP006 MCF7 24H-mitoxantrone-0.37	12/91	1.03×10^{-11}	1.81×10^{-9}
LJP006 MCF7 24H-mitoxantrone-3.33	13/122	2.20×10^{-11}	3.30×10^{-9}
LJP005 HEPG2 24H-mitoxantrone-1.11	13/130	4.96×10^{-11}	6.44×10^{-9}
LJP006 SKBR3 24H-mitoxantrone-0.04	10/71	2.95×10^{-10}	2.72×10^{-8}
LJP006 MCF10A 24H-mitoxantrone-3.33	12/155	5.43×10^{-9}	3.04×10^{-7}
LJP005 MCF7 24H-mitoxantrone-0.12	8/60	2.92×10^{-8}	1.27×10^{-6}
LJP006 HEPG2 24H-mitoxantrone-0.37	9/94	7.48×10^{-8}	2.82×10^{-6}
LJP005 SKBR3 24H-mitoxantrone-0.12	8/78	2.37×10^{-7}	7.36×10^{-6}
LJP006 A375 24H-mitoxantrone-10	9/108	2.50×10^{-7}	7.71×10^{-6}
LJP005 MCF10A 24H-mitoxantrone-1.11	9/111	3.16×10^{-7}	9.41×10^{-6}
LJP005 MDAMB231 3H-mitoxantrone-1.11	7/56	3.48×10^{-7}	1.02×10^{-5}
LJP006 HEPG2 24H-mitoxantrone-3.33	11/189	4.41×10^{-7}	1.24×10^{-5}
LJP006 MCF7 24H-mitoxantrone-1.11	9/123	7.57×10^{-7}	1.97×10^{-5}
LJP006 SKBR3 24H-mitoxantrone-1.11	9/124	8.10×10^{-7}	2.08×10^{-5}
LJP006 SKBR3 24H-mitoxantrone-0.12	8/92	8.53×10^{-7}	2.17×10^{-5}
LJP006 HA1E 24H-mitoxantrone-0.12	10/163	9.26×10^{-7}	2.33×10^{-5}
LJP006 SKBR3 24H-mitoxantrone-0.37	8/94	1.01×10^{-6}	2.48×10^{-5}
LJP006 BT20 24H-mitoxantrone-0.37	10/165	1.04×10^{-6}	2.55×10^{-5}
LJP006 SKBR3 3H-mitoxantrone-1.11	7/66	1.09×10^{-6}	2.66×10^{-5}
LJP006 MDAMB231 3H-mitoxantrone-1.11	6/43	1.27×10^{-6}	3.01×10^{-5}
LJP005 MCF10A 24H-mitoxantrone-10	8/102	1.88×10^{-6}	4.16×10^{-5}
LJP007 MCF7 24H-mitoxantrone-10	8/103	2.02×10^{-6}	4.39×10^{-5}
LJP005 MCF7 24H-mitoxantrone-1.11	8/110	3.32×10^{-6}	6.67×10^{-5}
LJP006 SKBR3 3H-mitoxantrone-0.37	7/79	3.72×10^{-6}	7.29×10^{-5}
LJP005 HEPG2 24H-mitoxantrone-3.33	10/194	4.46×10^{-6}	8.52×10^{-5}
LJP006 PC3 24H-mitoxantrone-0.37	6/53	4.47×10^{-6}	8.52×10^{-5}
LJP005 HA1E 24H-mitoxantrone-0.37	9/155	5.17×10^{-6}	9.71×10^{-5}
LJP006 HCC515 24H-mitoxantrone-1.11	9/156	5.45×10^{-6}	1.02×10^{-4}
LJP006 BT20 24H-mitoxantrone-1.11	9/157	5.74×10^{-6}	1.06×10^{-4}
LJP005 HCC515 24H-mitoxantrone-0.37	8/123	7.63×10^{-6}	1.34×10^{-4}
LJP006 BT20 24H-mitoxantrone-3.33	8/137	1.68×10^{-5}	2.55×10^{-4}
LJP006 PC3 24H-mitoxantrone-10	8/139	1.87×10^{-5}	2.78×10^{-4}
LJP005 MCF7 24H-mitoxantrone-10	6/68	1.93×10^{-5}	2.86×10^{-4}
LJP005 HA1E 24H-mitoxantrone-0.04	6/70	2.28×10^{-5}	3.31×10^{-4}
LJP006 MCF7 24H-mitoxantrone-0.12	6/71	2.47×10^{-5}	3.55×10^{-4}
LJP005 SKBR3 3H-mitoxantrone-0.12	5/45	3.17×10^{-5}	4.41×10^{-4}
LJP006 A549 24H-mitoxantrone-3.33	8/152	3.56×10^{-5}	4.84×10^{-4}
LJP007 PC3 24H-mitoxantrone-10	7/115	4.40×10^{-5}	5.80×10^{-4}
LJP005 HCC515 24H-mitoxantrone-3.33	8/158	4.68×10^{-5}	6.13×10^{-4}
LJP005 MCF10A 3H-mitoxantrone-3.33	5/49	4.83×10^{-5}	6.29×10^{-4}
LJP006 A549 24H-mitoxantrone-1.11	7/119	5.47×10^{-5}	6.99×10^{-4}
LJP009 PC3 24H-mitoxantrone-10	8/162	5.59×10^{-5}	7.11×10^{-4}
LJP008 A375 24H-mitoxantrone-10	7/120	5.77×10^{-5}	7.32×10^{-4}
LJP005 SKBR3 24H-mitoxantrone-3.33	7/120	5.77×10^{-5}	7.31×10^{-4}
LJP006 HEPG2 24H-mitoxantrone-1.11	7/120	5.77×10^{-5}	7.30×10^{-4}
LJP005 HA1E 24H-mitoxantrone-0.12	7/121	6.09×10^{-5}	7.60×10^{-4}
LJP005 MDAMB231 24H-mitoxantrone-0.37	5/52	6.45×10^{-5}	7.92×10^{-4}
LJP005 PC3 24H-mitoxantrone-0.37	5/52	6.45×10^{-5}	7.91×10^{-4}
LJP005 SKBR3 3H-mitoxantrone-1.11	6/85	6.87×10^{-5}	8.38×10^{-4}
LJP006 LNCAP 24H-mitoxantrone-0.37	6/86	7.33×10^{-5}	8.85×10^{-4}
LJP006 HME1 24H-mitoxantrone-1.11	7/125	7.48×10^{-5}	8.99×10^{-4}
LJP006 SKBR3 3H-mitoxantrone-0.12	5/54	7.75×10^{-5}	9.26×10^{-4}

S20 Table: (Continued)

LJP005 HCC515 24H-mitoxantrone-1.11	7/126	7.87×10^{-5}	9.37×10^{-4}
CPC020 PC3 6H-mitoxantrone dihydrochloride-10.0	6/89	8.89×10^{-5}	1.04×10^{-3}
LJP005 SKBR3 3H-mitoxantrone-0.37	5/56	9.23×10^{-5}	1.07×10^{-3}
LJP005 A549 24H-mitoxantrone-3.33	8/182	1.26×10^{-4}	1.40×10^{-3}
LJP006 HA1E 24H-mitoxantrone-0.37	8/183	1.31×10^{-4}	1.43×10^{-3}
CPC020 PC3 24H-mitoxantrone dihydrochloride-10.0	8/189	1.64×10^{-4}	1.72×10^{-3}
LJP005 A375 24H-mitoxantrone-0.12	7/145	1.89×10^{-4}	1.93×10^{-3}
LJP005 MCF10A 24H-mitoxantrone-0.37	6/106	2.33×10^{-4}	2.31×10^{-3}
LJP006 HME1 3H-mitoxantrone-3.33	5/70	2.67×10^{-4}	2.60×10^{-3}
LJP006 A549 24H-mitoxantrone-0.12	6/109	2.71×10^{-4}	2.63×10^{-3}
LJP006 HT29 24H-mitoxantrone-0.37	4/39	2.80×10^{-4}	2.71×10^{-3}
LJP006 SKBR3 3H-mitoxantrone-3.33	4/39	2.80×10^{-4}	2.71×10^{-3}
LJP006 MCF10A 3H-mitoxantrone-0.12	4/40	3.09×10^{-4}	2.93×10^{-3}
LJP006 A549 24H-mitoxantrone-0.37	6/112	3.14×10^{-4}	2.97×10^{-3}
LJP006 HCC515 24H-mitoxantrone-3.33	7/160	3.45×10^{-4}	3.22×10^{-3}
LJP006 MDAMB231 3H-mitoxantrone-0.37	5/74	3.46×10^{-4}	3.21×10^{-3}
LJP006 MCF10A 3H-mitoxantrone-0.37	4/42	3.74×10^{-4}	3.44×10^{-3}
LJP005 SKBR3 24H-mitoxantrone-1.11	6/117	3.97×10^{-4}	3.62×10^{-3}
LJP006 PC3 24H-mitoxantrone-1.11	6/117	3.97×10^{-4}	3.62×10^{-3}
LJP005 MCF10A 3H-mitoxantrone-1.11	4/43	4.10×10^{-4}	3.72×10^{-3}
LJP006 HCC515 24H-mitoxantrone-0.37	6/121	4.75×10^{-4}	4.20×10^{-3}
LJP007 HT29 24H-mitoxantrone-10	6/123	5.18×10^{-4}	4.53×10^{-3}
LJP006 MDAMB231 3H-mitoxantrone-3.33	4/46	5.31×10^{-4}	4.62×10^{-3}
LJP005 HT29 24H-mitoxantrone-0.37	5/82	5.56×10^{-4}	4.82×10^{-3}
LJP005 MCF7 24H-mitoxantrone-0.37	5/82	5.56×10^{-4}	4.82×10^{-3}
LJP005 A549 24H-mitoxantrone-10	7/175	5.90×10^{-4}	5.03×10^{-3}
LJP006 HA1E 24H-mitoxantrone-0.04	6/127	6.13×10^{-4}	5.22×10^{-3}
LJP005 HS578T 3H-mitoxantrone-0.37	4/49	6.77×10^{-4}	5.68×10^{-3}
CPC020 A375 6H-mitoxantrone dihydrochloride-10.0	5/86	6.92×10^{-4}	5.79×10^{-3}
CPC005 HA1E 24H-mitoxantrone dihydrochloride-10.0	6/131	7.22×10^{-4}	6.02×10^{-3}
LJP005 MCF7 24H-mitoxantrone-3.33	6/131	7.22×10^{-4}	6.01×10^{-3}
LJP006 A375 24H-mitoxantrone-3.33	5/87	7.29×10^{-4}	6.06×10^{-3}
LJP006 A549 24H-mitoxantrone-10	7/183	7.69×10^{-4}	6.28×10^{-3}
LJP005 SKBR3 3H-mitoxantrone-3.33	4/55	1.05×10^{-3}	8.15×10^{-3}
LJP005 HS578T 24H-mitoxantrone-3.33	6/141	1.06×10^{-3}	8.20×10^{-3}
LJP006 HEPG2 24H-mitoxantrone-0.12	4/60	1.45×10^{-3}	1.06×10^{-2}
LJP006 HS578T 24H-mitoxantrone-0.12	4/60	1.45×10^{-3}	1.06×10^{-2}
CPC005 A375 24H-mitoxantrone dihydrochloride-10.0	7/209	1.66×10^{-3}	1.18×10^{-2}
LJP009 MCF7 24H-mitoxantrone-10	4/63	1.74×10^{-3}	1.23×10^{-2}
LJP008 MCF7 24H-mitoxantrone-10	4/63	1.74×10^{-3}	1.23×10^{-2}
LJP006 A375 24H-mitoxantrone-1.11	5/107	1.84×10^{-3}	1.29×10^{-2}
CPC005 PC3 24H-mitoxantrone dihydrochloride-10.0	6/158	1.89×10^{-3}	1.32×10^{-2}
LJP005 SKBR3 24H-mitoxantrone-0.04	4/65	1.96×10^{-3}	1.35×10^{-2}
LJP006 SKBR3 24H-mitoxantrone-10	5/109	2.00×10^{-3}	1.38×10^{-2}
LJP006 HT29 24H-mitoxantrone-3.33	6/167	2.50×10^{-3}	1.66×10^{-2}
LJP005 BT20 24H-mitoxantrone-10	4/70	2.57×10^{-3}	1.70×10^{-2}
CPD001 PC3 24H-mitoxantrone dihydrochloride-10.0	6/169	2.65×10^{-3}	1.74×10^{-2}
LJP005 A549 24H-mitoxantrone-1.11	5/117	2.72×10^{-3}	1.78×10^{-2}
LJP006 LNCAP 24H-mitoxantrone-0.04	4/72	2.84×10^{-3}	1.85×10^{-2}
LJP006 HEPG2 24H-mitoxantrone-10	6/172	2.90×10^{-3}	1.87×10^{-2}
LJP006 HME1 3H-mitoxantrone-0.37	3/36	3.11×10^{-3}	1.98×10^{-2}

S20 Table: (Continued)

LJP006 SKBR3 24H-mitoxantrone-3.33	5/121	3.15×10^{-3}	1.99×10^{-2}
LJP005 HA1E 24H-mitoxantrone-3.33	5/125	3.62×10^{-3}	2.24×10^{-2}
LJP009 A375 24H-mitoxantrone-10	4/77	3.63×10^{-3}	2.24×10^{-2}
LJP005 MCF7 3H-mitoxantrone-3.33	4/78	3.80×10^{-3}	2.32×10^{-2}
LJP007 A375 24H-mitoxantrone-10	5/127	3.87×10^{-3}	2.35×10^{-2}
LJP005 HS578T 3H-mitoxantrone-3.33	3/39	3.92×10^{-3}	2.38×10^{-2}
LJP008 A549 24H-mitoxantrone-10	5/129	4.14×10^{-3}	2.49×10^{-2}
LJP006 LNCAP 24H-mitoxantrone-1.11	5/131	4.41×10^{-3}	2.61×10^{-2}
LJP005 HT29 24H-mitoxantrone-10	5/134	4.86×10^{-3}	2.80×10^{-2}
CPC020 HT29 6H-mitoxantrone dihydrochloride-10.0	3/43	5.16×10^{-3}	2.94×10^{-2}
LJP006 MCF7 24H-mitoxantrone-10	4/87	5.60×10^{-3}	3.13×10^{-2}
LJP006 BT20 24H-mitoxantrone-0.12	4/88	5.83×10^{-3}	3.24×10^{-2}
LJP006 HS578T 3H-mitoxantrone-10	3/46	6.24×10^{-3}	3.40×10^{-2}
LJP006 MCF10A 3H-mitoxantrone-3.33	3/50	7.87×10^{-3}	4.09×10^{-2}
LJP006 MDAMB231 24H-mitoxantrone-0.12	3/50	7.87×10^{-3}	4.09×10^{-2}
CPC005 MCF7 6H-mitoxantrone dihydrochloride-10.0	4/96	7.90×10^{-3}	4.10×10^{-2}
LJP005 HT29 24H-mitoxantrone-1.11	4/96	7.90×10^{-3}	4.09×10^{-2}
LJP005 HS578T 24H-mitoxantrone-0.37	5/152	8.19×10^{-3}	4.22×10^{-2}
LJP005 MDAMB231 3H-mitoxantrone-3.33	2/17	8.29×10^{-3}	4.26×10^{-2}
LJP006 HT29 24H-mitoxantrone-10	4/98	8.49×10^{-3}	4.32×10^{-2}
LJP006 MCF10A 3H-mitoxantrone-1.11	3/52	8.77×10^{-3}	4.44×10^{-2}
LJP005 MCF10A 24H-mitoxantrone-3.33	4/100	9.10×10^{-3}	4.58×10^{-2}
