# Supplementary material for: A new advanced in silico drug discovery method for novel coronavirus (SARS-CoV-2) with tensor decomposition-based unsupervised feature extraction
Source: PLoS One. 2020 Sep 11;15(9):e0238907. doi: 10.1371/journal.pone.0238907 (PMC7485840; doi:10.1371/journal.pone.0238907)
Supplement: S21 Table — Mitoxantrone significantly affects the expression of the selected 163 genes as evident in the “LINCS L1000 Chem Pert down” category in Enrichr. The last number after the—is dose density. (PDF) [file pone.0238907.s021.pdf]

S21 Table: Mitoxantrone significantly affects the expression of the selected 163 genes as evident in the "LINCS L1000 Chem Pert down" category in Enrichr. The last number after the - is dose density.

Term	Overlap	P-value	Adjusted P-value
LINCS L1000 Chem Pert down			
LJP005 HCC515 24H-mitoxantrone-0.12	17/120	1.22×10^{-16}	2.02×10^{-13}
LJP007 HT29 24H-mitoxantrone-10	15/123	7.94×10^{-14}	3.13×10^{-11}
LJP005 HEPG2 24H-mitoxantrone-10	13/82	1.18×10^{-13}	4.40×10^{-11}
LJP005 SKBR3 24H-mitoxantrone-10	12/70	4.03×10^{-13}	1.24×10^{-10}
LJP005 BT20 3H-mitoxantrone-3.33	10/40	6.72×10^{-13}	1.89×10^{-10}
LJP008 PC3 24H-mitoxantrone-10	15/146	1.02×10^{-12}	2.69×10^{-10}
LJP006 SKBR3 24H-mitoxantrone-3.33	14/120	1.03×10^{-12}	2.68×10^{-10}
LJP005 A549 24H-mitoxantrone-3.33	16/185	2.53×10^{-12}	5.42×10^{-10}
LJP006 HCC515 24H-mitoxantrone-0.12	11/82	6.38×10^{-11}	7.36×10^{-9}
LJP006 HEPG2 24H-mitoxantrone-10	11/87	1.23×10^{-10}	1.24×10^{-8}
LJP005 SKBR3 24H-mitoxantrone-3.33	12/115	1.71×10^{-10}	1.63×10^{-8}
LJP006 HCC515 24H-mitoxantrone-0.37	11/92	2.28×10^{-10}	2.08×10^{-8}
LJP005 SKBR3 3H-mitoxantrone-10	9/52	3.46×10^{-10}	2.94×10^{-8}
LJP005 HA1E 24H-mitoxantrone-10	11/98	4.56×10^{-10}	3.66×10^{-8}
LJP006 BT20 24H-mitoxantrone-10	10/75	5.15×10^{-10}	4.03×10^{-8}
LJP007 A549 24H-mitoxantrone-10	7/25	8.91×10^{-10}	6.36×10^{-8}
LJP007 HCC515 24H-mitoxantrone-10	10/82	1.27×10^{-9}	8.48×10^{-8}
LJP005 HT29 24H-mitoxantrone-10	11/110	1.60×10^{-9}	1.03×10^{-7}
LJP005 HCC515 24H-mitoxantrone-10	11/111	1.76×10^{-9}	1.11×10^{-7}
LJP006 HS578T 24H-mitoxantrone-10	10/86	2.04×10^{-9}	1.27×10^{-7}
LJP005 PC3 24H-mitoxantrone-10	11/114	2.34×10^{-9}	1.43×10^{-7}
LJP006 HS578T 24H-mitoxantrone-3.33	11/115	2.57×10^{-9}	1.54×10^{-7}
LJP009 A375 24H-mitoxantrone-10	10/93	4.43×10^{-9}	2.44×10^{-7}
LJP006 HCC515 24H-mitoxantrone-10	10/94	4.92×10^{-9}	2.65×10^{-7}
LJP006 HS578T 3H-mitoxantrone-1.11	9/74	8.93×10^{-9}	4.42×10^{-7}
LJP009 HCC515 24H-mitoxantrone-10	9/75	1.01×10^{-8}	4.87×10^{-7}
LJP009 HT29 24H-mitoxantrone-10	10/104	1.32×10^{-8}	6.08×10^{-7}
LJP008 HCC515 24H-mitoxantrone-10	9/78	1.43×10^{-8}	6.50×10^{-7}
LJP009 HEPG2 24H-mitoxantrone-10	10/108	1.91×10^{-8}	8.23×10^{-7}
LJP006 MCF10A 3H-mitoxantrone-10	9/81	2.01×10^{-8}	8.50×10^{-7}
LJP006 MCF7 24H-mitoxantrone-10	8/61	3.34×10^{-8}	1.31×10^{-6}
LJP005 HS578T 24H-mitoxantrone-0.37	11/152	4.83×10^{-8}	1.82×10^{-6}
LJP008 HEPG2 24H-mitoxantrone-10	9/90	5.11×10^{-8}	1.91×10^{-6}
LJP006 HS578T 24H-mitoxantrone-0.37	10/122	6.17×10^{-8}	2.24×10^{-6}
LJP008 A549 24H-mitoxantrone-10	9/93	6.81×10^{-8}	2.42×10^{-6}
LJP005 HS578T 3H-mitoxantrone-3.33	9/93	6.81×10^{-8}	2.42×10^{-6}
LJP005 HCC515 24H-mitoxantrone-3.33	9/96	8.99×10^{-8}	3.05×10^{-6}
LJP005 BT20 24H-mitoxantrone-10	9/101	1.40×10^{-7}	4.48×10^{-6}
LJP005 HCC515 24H-mitoxantrone-1.11	9/101	1.40×10^{-7}	4.47×10^{-6}
LJP006 A375 24H-mitoxantrone-10	10/134	1.50×10^{-7}	4.75×10^{-6}
LJP005 HS578T 3H-mitoxantrone-1.11	9/102	1.52×10^{-7}	4.81×10^{-6}
LJP005 MCF7 24H-mitoxantrone-10	7/50	1.56×10^{-7}	4.92×10^{-6}
LJP008 HA1E 24H-mitoxantrone-10	9/103	1.66×10^{-7}	5.19×10^{-6}
LJP006 SKBR3 24H-mitoxantrone-10	8/78	2.37×10^{-7}	6.96×10^{-6}
LJP006 LNCAP 3H-mitoxantrone-3.33	7/54	2.69×10^{-7}	7.74×10^{-6}
LJP005 HA1E 24H-mitoxantrone-0.37	9/109	2.70×10^{-7}	7.75×10^{-6}
LJP005 A549 24H-mitoxantrone-10	10/143	2.76×10^{-7}	7.88×10^{-6}
LJP006 HT29 24H-mitoxantrone-10	8/80	2.89×10^{-7}	8.18×10^{-6}
LJP006 PC3 24H-mitoxantrone-10	9/110	2.92×10^{-7}	8.24×10^{-6}
LJP005 BT20 3H-mitoxantrone-1.11	7/55	3.07×10^{-7}	8.58×10^{-6}
LJP006 HA1E 24H-mitoxantrone-1.11	9/113	3.68×10^{-7}	9.95×10^{-6}
LJP005 HS578T 24H-mitoxantrone-0.12	8/85	4.63×10^{-7}	1.21×10^{-5}
LJP006 HS578T 3H-mitoxantrone-3.33	8/92	8.53×10^{-7}	2.02×10^{-5}
LJP005 MCF10A 3H-mitoxantrone-1.11	7/65	9.82×10^{-7}	2.28×10^{-5}

S21 Table: (Continued)

LJP006 LNCAP 24H-mitoxantrone-1.11	11/207	1.09×10^{-6}	2.49×10^{-5}
LJP006 LNCAP 24H-mitoxantrone-10	8/95	1.09×10^{-6}	2.50×10^{-5}
LJP006 SKBR3 3H-mitoxantrone-10	7/70	1.63×10^{-6}	3.50×10^{-5}
LJP006 HME1 24H-mitoxantrone-0.37	6/46	1.91×10^{-6}	3.97×10^{-5}
LJP005 MDAMB231 3H-mitoxantrone-3.33	7/72	1.98×10^{-6}	4.10×10^{-5}
LJP006 MCF10A 3H-mitoxantrone-0.12	6/48	2.47×10^{-6}	4.92×10^{-5}
LJP005 HA1E 24H-mitoxantrone-0.12	7/77	3.13×10^{-6}	6.01×10^{-5}
LJP006 HCC515 24H-mitoxantrone-3.33	6/51	3.55×10^{-6}	6.66×10^{-5}
LJP006 MCF7 3H-mitoxantrone-10	6/52	3.99×10^{-6}	7.40×10^{-5}
LJP006 PC3 24H-mitoxantrone-3.33	9/152	4.41×10^{-6}	8.06×10^{-5}
LJP009 MCF7 24H-mitoxantrone-10	8/117	5.26×10^{-6}	9.40×10^{-5}
LJP005 HA1E 24H-mitoxantrone-3.33	7/86	6.57×10^{-6}	1.13×10^{-4}
LJP005 A375 24H-mitoxantrone-10	8/121	6.76×10^{-6}	1.16×10^{-4}
LJP008 MCF7 24H-mitoxantrone-10	6/57	6.88×10^{-6}	1.18×10^{-4}
LJP009 PC3 24H-mitoxantrone-10	9/163	7.79×10^{-6}	1.30×10^{-4}
LJP006 MDAMB231 24H-mitoxantrone-3.33	8/124	8.10×10^{-6}	1.35×10^{-4}
LJP007 A375 24H-mitoxantrone-10	7/89	8.26×10^{-6}	1.37×10^{-4}
LJP006 A375 24H-mitoxantrone-3.33	6/59	8.42×10^{-6}	1.40×10^{-4}
LJP005 HT29 24H-mitoxantrone-0.37	6/60	9.30×10^{-6}	1.52×10^{-4}
LJP006 MDAMB231 3H-mitoxantrone-3.33	8/127	9.66×10^{-6}	1.57×10^{-4}
LJP005 HS578T 24H-mitoxantrone-1.11	7/92	1.03×10^{-5}	1.66×10^{-4}
LJP006 HME1 24H-mitoxantrone-1.11	6/62	1.13×10^{-5}	1.79×10^{-4}
LJP005 HEPG2 24H-mitoxantrone-3.33	8/132	1.28×10^{-5}	2.00×10^{-4}
LJP006 HA1E 24H-mitoxantrone-0.12	8/133	1.35×10^{-5}	2.11×10^{-4}
LJP006 BT20 24H-mitoxantrone-0.12	6/64	1.36×10^{-5}	2.11×10^{-4}
LJP005 MCF10A 3H-mitoxantrone-10	6/65	1.48×10^{-5}	2.27×10^{-4}
LJP005 HT29 24H-mitoxantrone-1.11	7/98	1.56×10^{-5}	2.36×10^{-4}
LJP006 HA1E 24H-mitoxantrone-10	5/40	1.77×10^{-5}	2.63×10^{-4}
LJP006 MCF7 3H-mitoxantrone-1.11	5/40	1.77×10^{-5}	2.63×10^{-4}
LJP005 A549 24H-mitoxantrone-1.11	9/183	1.97×10^{-5}	2.88×10^{-4}
LJP006 MCF10A 24H-mitoxantrone-0.12	7/103	2.16×10^{-5}	3.12×10^{-4}
LJP005 MDAMB231 3H-mitoxantrone-1.11	6/72	2.68×10^{-5}	3.77×10^{-4}
LJP006 MDAMB231 3H-mitoxantrone-1.11	6/73	2.90×10^{-5}	4.01×10^{-4}
LJP005 MDAMB231 3H-mitoxantrone-10	6/75	3.38×10^{-5}	4.59×10^{-4}
LJP005 SKBR3 24H-mitoxantrone-1.11	6/77	3.93×10^{-5}	5.20×10^{-4}
LJP006 MDAMB231 3H-mitoxantrone-10	7/114	4.16×10^{-5}	5.43×10^{-4}
LJP006 HEPG2 24H-mitoxantrone-3.33	7/117	4.91×10^{-5}	6.23×10^{-4}
LJP006 BT20 24H-mitoxantrone-1.11	6/81	5.24×10^{-5}	6.61×10^{-4}
LJP006 MCF10A 3H-mitoxantrone-3.33	6/82	5.61×10^{-5}	6.99×10^{-4}
LJP006 HS578T 24H-mitoxantrone-1.11	6/84	6.43×10^{-5}	7.85×10^{-4}
LJP005 HCC515 24H-mitoxantrone-0.37	6/85	6.87×10^{-5}	8.30×10^{-4}
LJP006 A549 24H-mitoxantrone-3.33	8/167	6.93×10^{-5}	8.34×10^{-4}
LJP007 HA1E 24H-mitoxantrone-10	6/87	7.83×10^{-5}	9.17×10^{-4}
LJP005 HA1E 24H-mitoxantrone-0.04	5/55	8.46×10^{-5}	9.85×10^{-4}
LJP005 HS578T 3H-mitoxantrone-10	5/55	8.46×10^{-5}	9.84×10^{-4}
LJP006 LNCAP 3H-mitoxantrone-10	5/57	1.01×10^{-4}	1.13×10^{-3}
LJP005 A375 24H-mitoxantrone-3.33	6/92	1.07×10^{-4}	1.19×10^{-3}
LJP006 MDAMB231 24H-mitoxantrone-10	4/31	1.13×10^{-4}	1.24×10^{-3}
LJP005 MCF7 3H-mitoxantrone-10	6/93	1.13×10^{-4}	1.25×10^{-3}
LJP006 MDAMB231 24H-mitoxantrone-0.37	5/59	1.19×10^{-4}	1.30×10^{-3}
LJP005 PC3 24H-mitoxantrone-1.11	6/94	1.20×10^{-4}	1.32×10^{-3}
LJP006 A549 24H-mitoxantrone-10	6/94	1.20×10^{-4}	1.31×10^{-3}
LJP005 HS578T 24H-mitoxantrone-3.33	6/96	1.35×10^{-4}	1.46×10^{-3}
LJP005 BT20 3H-mitoxantrone-10	5/61	1.39×10^{-4}	1.49×10^{-3}
LJP006 HME1 24H-mitoxantrone-10	5/61	1.39×10^{-4}	1.49×10^{-3}
LJP006 HA1E 24H-mitoxantrone-3.33	6/97	1.43×10^{-4}	1.53×10^{-3}
LJP006 HME1 24H-mitoxantrone-3.33	5/63	1.62×10^{-4}	1.70×10^{-3}

S21 Table: (Continued)

LJP006 MCF10A 3H-mitoxantrone-1.11	5/63	1.62×10^{-4}	1.70×10^{-3}
LJP007 HEPG2 24H-mitoxantrone-10	5/64	1.75×10^{-4}	1.80×10^{-3}
LJP006 LNCAP 24H-mitoxantrone-3.33	6/101	1.79×10^{-4}	1.84×10^{-3}
LJP006 LNCAP 24H-mitoxantrone-0.37	8/195	2.03×10^{-4}	2.04×10^{-3}
LJP005 MDAMB231 3H-mitoxantrone-0.04	4/37	2.28×10^{-4}	2.27×10^{-3}
LJP006 HT29 24H-mitoxantrone-3.33	7/150	2.33×10^{-4}	2.31×10^{-3}
LJP005 MCF10A 3H-mitoxantrone-3.33	4/38	2.53×10^{-4}	2.48×10^{-3}
LJP006 HEPG2 24H-mitoxantrone-1.11	6/108	2.58×10^{-4}	2.51×10^{-3}
LJP006 HS578T 3H-mitoxantrone-10	5/71	2.85×10^{-4}	2.73×10^{-3}
LJP005 MDAMB231 24H-mitoxantrone-10	3/17	3.32×10^{-4}	3.11×10^{-3}
LJP005 PC3 24H-mitoxantrone-0.12	4/41	3.40×10^{-4}	3.17×10^{-3}
LJP005 PC3 24H-mitoxantrone-3.33	6/116	3.79×10^{-4}	3.45×10^{-3}
LJP007 MCF7 24H-mitoxantrone-10	6/120	4.54×10^{-4}	4.02×10^{-3}
LJP006 MCF7 24H-mitoxantrone-1.11	6/121	4.75×10^{-4}	4.18×10^{-3}
LJP006 HME1 3H-mitoxantrone-3.33	5/81	5.26×10^{-4}	4.57×10^{-3}
LJP005 HA1E 24H-mitoxantrone-1.11	5/82	5.56×10^{-4}	4.78×10^{-3}
LJP005 HT29 24H-mitoxantrone-3.33	7/177	6.32×10^{-4}	5.30×10^{-3}
LJP006 A549 24H-mitoxantrone-1.11	6/130	6.93×10^{-4}	5.73×10^{-3}
LJP006 MCF7 3H-mitoxantrone-3.33	4/50	7.31×10^{-4}	5.98×10^{-3}
LJP008 HT29 24H-mitoxantrone-10	4/55	1.05×10^{-3}	8.16×10^{-3}
LJP006 HS578T 24H-mitoxantrone-0.12	4/56	1.12×10^{-3}	8.61×10^{-3}
LJP005 MDAMB231 24H-mitoxantrone-3.33	3/28	1.50×10^{-3}	1.10×10^{-2}
LJP006 SKBR3 3H-mitoxantrone-3.33	4/61	1.55×10^{-3}	1.12×10^{-2}
LJP008 A375 24H-mitoxantrone-10	5/103	1.56×10^{-3}	1.13×10^{-2}
LJP005 MCF10A 24H-mitoxantrone-0.04	4/62	1.64×10^{-3}	1.18×10^{-2}
LJP005 BT20 24H-mitoxantrone-3.33	4/64	1.85×10^{-3}	1.30×10^{-2}
LJP006 HME1 3H-mitoxantrone-10	4/64	1.85×10^{-3}	1.30×10^{-2}
LJP006 PC3 24H-mitoxantrone-0.37	4/64	1.85×10^{-3}	1.30×10^{-2}
LJP006 MDAMB231 24H-mitoxantrone-0.12	3/31	2.02×10^{-3}	1.40×10^{-2}
LJP005 MCF7 3H-mitoxantrone-1.11	4/66	2.07×10^{-3}	1.43×10^{-2}
LJP006 HA1E 24H-mitoxantrone-0.37	5/111	2.16×10^{-3}	1.49×10^{-2}
LJP006 BT20 24H-mitoxantrone-0.37	4/69	2.44×10^{-3}	1.63×10^{-2}
LJP005 SKBR3 24H-mitoxantrone-0.37	4/72	2.84×10^{-3}	1.86×10^{-2}
LJP005 HS578T 3H-mitoxantrone-0.37	3/36	3.11×10^{-3}	2.01×10^{-2}
LJP006 HEPG2 24H-mitoxantrone-0.37	5/125	3.62×10^{-3}	2.27×10^{-2}
LJP006 A549 24H-mitoxantrone-0.12	5/129	4.14×10^{-3}	2.52×10^{-2}
LJP006 MCF7 24H-mitoxantrone-3.33	4/81	4.35×10^{-3}	2.61×10^{-2}
CPC020 HT29 6H-mitoxantrone dihydrochloride-10.0	6/190	4.71×10^{-3}	2.80×10^{-2}
LJP005 A375 24H-mitoxantrone-1.11	4/83	4.74×10^{-3}	2.81×10^{-2}
LJP006 HME1 3H-mitoxantrone-1.11	3/43	5.16×10^{-3}	2.99×10^{-2}
LJP006 HT29 24H-mitoxantrone-0.37	3/43	5.16×10^{-3}	2.99×10^{-2}
LJP006 BT20 24H-mitoxantrone-3.33	4/86	5.38×10^{-3}	3.10×10^{-2}
LJP006 LNCAP 3H-mitoxantrone-1.11	3/46	6.24×10^{-3}	3.47×10^{-2}
LJP006 HCC515 24H-mitoxantrone-1.11	4/91	6.56×10^{-3}	3.62×10^{-2}
LJP006 MDAMB231 3H-mitoxantrone-0.37	3/47	6.63×10^{-3}	3.64×10^{-2}
LJP006 SKBR3 3H-mitoxantrone-1.11	3/47	6.63×10^{-3}	3.64×10^{-2}
CPC020 PC3 6H-mitoxantrone dihydrochloride-10.0	6/205	6.77×10^{-3}	3.71×10^{-2}
LJP005 BT20 24H-mitoxantrone-1.11	3/48	7.03×10^{-3}	3.84×10^{-2}
LJP009 A549 24H-mitoxantrone-10	3/48	7.03×10^{-3}	3.83×10^{-2}
LJP005 SKBR3 3H-mitoxantrone-3.33	3/48	7.03×10^{-3}	3.83×10^{-2}
LJP006 SKBR3 24H-mitoxantrone-1.11	4/94	7.35×10^{-3}	3.97×10^{-2}
LJP005 SKBR3 24H-mitoxantrone-0.04	3/49	7.44×10^{-3}	4.00×10^{-2}
LJP005 MCF10A 24H-mitoxantrone-1.11	5/149	7.55×10^{-3}	4.04×10^{-2}
LJP007 PC3 24H-mitoxantrone-10	4/97	8.19×10^{-3}	4.33×10^{-2}
