# Supplementary material for: A new advanced in silico drug discovery method for novel coronavirus (SARS-CoV-2) with tensor decomposition-based unsupervised feature extraction
Source: PLoS One. 2020 Sep 11;15(9):e0238907. doi: 10.1371/journal.pone.0238907 (PMC7485840; doi:10.1371/journal.pone.0238907)
Supplement: S22 Table — QL-XII-47 significantly affects the expression of the selected 163 genes as evident in the “LINCS L1000 Chem Pert up” category in Enrichr. The last number after the—is dose density. (PDF) [file pone.0238907.s022.pdf]

S22 Table: QL-XII-47 significantly affects the expression of the selected 163 genes as evident in the "LINCS L1000 Chem Pert up" category in Enrichr. The last number after the - is dose density.

Term	Overlap	P-value	Adjusted P-value
LINCS L1000 Chem Pert up			
LJP006 MCF7 24H-QL-XII-47-3.33	18/163	1.37×10^{-15}	1.13×10^{-12}
LJP006 A549 24H-QL-XII-47-10	19/219	1.89×10^{-14}	9.96×10^{-12}
LJP006 HME1 24H-QL-XII-47-1.11	15/153	2.04×10^{-12}	5.07×10^{-10}
LJP006 MCF7 24H-QL-XII-47-1.11	14/131	3.49×10^{-12}	7.65×10^{-10}
LJP006 LNCAP 24H-QL-XII-47-0.37	13/122	2.20×10^{-11}	3.32×10^{-9}
LJP006 SKBR3 24H-QL-XII-47-0.37	12/99	2.86×10^{-11}	4.12×10^{-9}
LJP006 MCF10A 3H-QL-XII-47-10	12/103	4.60×10^{-11}	6.10×10^{-9}
LJP006 HME1 24H-QL-XII-47-0.37	12/106	6.49×10^{-11}	7.87×10^{-9}
LJP006 HCC515 24H-QL-XII-47-10	13/143	1.66×10^{-10}	1.68×10^{-8}
LJP006 A375 24H-QL-XII-47-0.12	8/38	6.51×10^{-10}	5.27×10^{-8}
LJP006 BT20 24H-QL-XII-47-0.04	9/56	6.93×10^{-10}	5.58×10^{-8}
LJP006 HT29 24H-QL-XII-47-10	11/107	1.18×10^{-9}	8.48×10^{-8}
LJP006 MCF7 24H-QL-XII-47-10	14/204	1.34×10^{-9}	9.34×10^{-8}
LJP006 MDAMB231 3H-QL-XII-47-10	11/130	9.46×10^{-9}	4.91×10^{-7}
LJP006 MCF7 24H-QL-XII-47-0.37	10/103	1.21×10^{-8}	6.00×10^{-7}
LJP006 HEPG2 24H-QL-XII-47-1.11	11/134	1.30×10^{-8}	6.37×10^{-7}
LJP006 HME1 3H-QL-XII-47-3.33	11/135	1.41×10^{-8}	6.81×10^{-7}
LJP006 BT20 24H-QL-XII-47-0.12	12/172	1.76×10^{-8}	8.21×10^{-7}
LJP006 A549 24H-QL-XII-47-3.33	7/49	1.35×10^{-7}	4.65×10^{-6}
LJP006 HME1 3H-QL-XII-47-1.11	10/135	1.61×10^{-7}	5.37×10^{-6}
LJP006 A375 24H-QL-XII-47-3.33	11/172	1.71×10^{-7}	5.64×10^{-6}
LJP006 MCF7 3H-QL-XII-47-10	9/111	3.16×10^{-7}	9.38×10^{-6}
LJP006 LNCAP 24H-QL-XII-47-1.11	12/227	3.67×10^{-7}	1.07×10^{-5}
LJP006 HME1 3H-QL-XII-47-0.37	9/113	3.68×10^{-7}	1.07×10^{-5}
LJP006 HEPG2 24H-QL-XII-47-0.12	6/37	5.04×10^{-7}	1.39×10^{-5}
LJP006 MCF7 3H-QL-XII-47-3.33	8/86	5.07×10^{-7}	1.39×10^{-5}
LJP006 HEPG2 24H-QL-XII-47-3.33	9/120	6.14×10^{-7}	1.65×10^{-5}
LJP006 MCF10A 24H-QL-XII-47-3.33	9/121	6.59×10^{-7}	1.75×10^{-5}
LJP006 MCF7 3H-QL-XII-47-1.11	7/62	7.08×10^{-7}	1.86×10^{-5}
LJP006 MDAMB231 3H-QL-XII-47-0.37	7/64	8.83×10^{-7}	2.23×10^{-5}
LJP006 MDAMB231 3H-QL-XII-47-1.11	8/99	1.50×10^{-6}	3.44×10^{-5}
LJP006 BT20 3H-QL-XII-47-1.11	7/71	1.80×10^{-6}	4.02×10^{-5}
LJP006 HT29 24H-QL-XII-47-0.12	5/27	2.36×10^{-6}	5.04×10^{-5}
LJP006 SKBR3 24H-QL-XII-47-10	10/191	3.88×10^{-6}	7.56×10^{-5}
LJP006 BT20 3H-QL-XII-47-0.12	4/16	7.17×10^{-6}	1.27×10^{-4}
LJP006 BT20 3H-QL-XII-47-3.33	6/59	8.42×10^{-6}	1.44×10^{-4}
LJP006 MDAMB231 3H-QL-XII-47-3.33	8/127	9.66×10^{-6}	1.61×10^{-4}
LJP006 BT20 3H-QL-XII-47-0.37	6/64	1.36×10^{-5}	2.14×10^{-4}
LJP006 SKBR3 3H-QL-XII-47-10	6/66	1.62×10^{-5}	2.47×10^{-4}
LJP006 HME1 24H-QL-XII-47-3.33	8/146	2.66×10^{-5}	3.79×10^{-4}
LJP006 MCF10A 3H-QL-XII-47-3.33	6/76	3.65×10^{-5}	4.95×10^{-4}
LJP006 BT20 3H-QL-XII-47-0.04	4/24	3.98×10^{-5}	5.33×10^{-4}
LJP006 LNCAP 24H-QL-XII-47-3.33	9/203	4.45×10^{-5}	5.87×10^{-4}
LJP006 SKBR3 24H-QL-XII-47-3.33	9/203	4.45×10^{-5}	5.87×10^{-4}
LJP006 MCF10A 24H-QL-XII-47-10	8/158	4.68×10^{-5}	6.12×10^{-4}
LJP006 SKBR3 24H-QL-XII-47-1.11	8/163	5.84×10^{-5}	7.38×10^{-4}
LJP006 HME1 3H-QL-XII-47-0.12	5/52	6.45×10^{-5}	7.91×10^{-4}
LJP006 MDAMB231 24H-QL-XII-47-1.11	4/28	7.47×10^{-5}	8.99×10^{-4}
LJP006 SKBR3 3H-QL-XII-47-3.33	6/92	1.07×10^{-4}	1.21×10^{-3}
LJP006 BT20 24H-QL-XII-47-0.37	8/180	1.17×10^{-4}	1.30×10^{-3}
LJP006 HEPG2 24H-QL-XII-47-10	6/95	1.28×10^{-4}	1.41×10^{-3}
LJP006 SKBR3 3H-QL-XII-47-1.11	6/95	1.28×10^{-4}	1.41×10^{-3}
LJP006 HS578T 24H-QL-XII-47-10	9/233	1.28×10^{-4}	1.40×10^{-3}
LJP006 MCF7 3H-QL-XII-47-0.04	4/33	1.45×10^{-4}	1.56×10^{-3}

S22 Table: (Continued)

LJP006 A375 24H-QL-XII-47-1.11	6/103	1.99×10^{-4}	2.03×10^{-3}
LJP006 MCF7 24H-QL-XII-47-0.12	4/36	2.04×10^{-4}	2.07×10^{-3}
LJP006 MCF7 3H-QL-XII-47-0.37	4/37	2.28×10^{-4}	2.27×10^{-3}
LJP006 LNCAP 24H-QL-XII-47-0.12	5/83	5.88×10^{-4}	5.02×10^{-3}
LJP006 HS578T 3H-QL-XII-47-1.11	4/48	6.26×10^{-4}	5.30×10^{-3}
LJP006 A375 24H-QL-XII-47-10	6/131	7.22×10^{-4}	6.01×10^{-3}
LJP006 LNCAP 3H-QL-XII-47-1.11	4/51	7.89×10^{-4}	6.42×10^{-3}
LJP006 MDAMB231 24H-QL-XII-47-3.33	4/51	7.89×10^{-4}	6.41×10^{-3}
LJP006 BT20 24H-QL-XII-47-10	7/199	1.25×10^{-3}	9.39×10^{-3}
LJP006 HS578T 3H-QL-XII-47-3.33	4/59	1.37×10^{-3}	1.00×10^{-2}
LJP006 LNCAP 24H-QL-XII-47-10	7/212	1.80×10^{-3}	1.26×10^{-2}
LJP006 LNCAP 3H-QL-XII-47-3.33	4/66	2.07×10^{-3}	1.41×10^{-2}
LJP006 A549 24H-QL-XII-47-0.37	3/35	2.87×10^{-3}	1.85×10^{-2}
LJP006 LNCAP 3H-QL-XII-47-10	4/78	3.80×10^{-3}	2.32×10^{-2}
LJP006 BT20 24H-QL-XII-47-1.11	6/195	5.34×10^{-3}	3.02×10^{-2}
LJP006 HME1 24H-QL-XII-47-0.12	3/45	5.87×10^{-3}	3.24×10^{-2}
LJP006 MDAMB231 24H-QL-XII-47-10	4/95	7.62×10^{-3}	3.99×10^{-2}
LJP006 SKBR3 24H-QL-XII-47-0.12	3/53	9.24×10^{-3}	4.62×10^{-2}
