# Supplementary material for: A new advanced in silico drug discovery method for novel coronavirus (SARS-CoV-2) with tensor decomposition-based unsupervised feature extraction
Source: PLoS One. 2020 Sep 11;15(9):e0238907. doi: 10.1371/journal.pone.0238907 (PMC7485840; doi:10.1371/journal.pone.0238907)
Supplement: S23 Table — QL-XII-47 significantly affects the expression of the selected 163 genes as evident in the “LINCS L1000 Chem Pert down” category in Enrichr. The last number after the—is dose density. (PDF) [file pone.0238907.s023.pdf]

S23 Table: QL-XII-47 significantly affects the expression of the selected 163 genes as evident in the "LINCS L1000 Chem Pert down" category in Enrichr. The last number after the - is dose density.

Term	Overlap	P-value	Adjusted P-value
LINCS L1000 Chem Pert down			
LJP006 BT20 24H-QL-XII-47-10	19/169	1.52×10^{-16}	2.29×10^{-13}
LJP006 SKBR3 24H-QL-XII-47-3.33	19/180	4.98×10^{-16}	5.69×10^{-13}
LJP006 HA1E 24H-QL-XII-47-1.11	16/178	1.40×10^{-12}	3.48×10^{-10}
LJP006 HS578T 24H-QL-XII-47-10	15/184	2.95×10^{-11}	3.91×10^{-9}
LJP006 HA1E 24H-QL-XII-47-0.37	12/106	6.49×10^{-11}	7.46×10^{-9}
LJP006 HA1E 24H-QL-XII-47-3.33	15/195	6.76×10^{-11}	7.72×10^{-9}
LJP006 LNCAP 24H-QL-XII-47-10	13/151	3.28×10^{-10}	2.80×10^{-8}
LJP006 SKBR3 24H-QL-XII-47-1.11	14/185	3.72×10^{-10}	3.10×10^{-8}
LJP006 LNCAP 24H-QL-XII-47-3.33	13/168	1.23×10^{-9}	8.37×10^{-8}
LJP006 HS578T 24H-QL-XII-47-3.33	13/175	2.03×10^{-9}	1.27×10^{-7}
LJP006 BT20 24H-QL-XII-47-0.37	12/164	1.03×10^{-8}	4.95×10^{-7}
LJP006 HME1 3H-QL-XII-47-10	9/90	5.11×10^{-8}	1.91×10^{-6}
LJP006 BT20 24H-QL-XII-47-3.33	11/165	1.12×10^{-7}	3.71×10^{-6}
LJP006 HME1 24H-QL-XII-47-0.12	7/52	2.06×10^{-7}	6.21×10^{-6}
LJP006 A549 24H-QL-XII-47-10	10/146	3.35×10^{-7}	9.26×10^{-6}
LJP006 MCF7 24H-QL-XII-47-3.33	9/116	4.60×10^{-7}	1.20×10^{-5}
LJP006 LNCAP 3H-QL-XII-47-0.37	6/43	1.27×10^{-6}	2.83×10^{-5}
LJP006 HA1E 24H-QL-XII-47-10	8/98	1.38×10^{-6}	3.03×10^{-5}
LJP006 BT20 24H-QL-XII-47-1.11	9/135	1.65×10^{-6}	3.53×10^{-5}
LJP006 HEPG2 24H-QL-XII-47-3.33	10/176	1.86×10^{-6}	3.89×10^{-5}
LJP006 MCF10A 24H-QL-XII-47-10	8/107	2.69×10^{-6}	5.29×10^{-5}
LJP006 HS578T 24H-QL-XII-47-1.11	6/49	2.80×10^{-6}	5.47×10^{-5}
LJP006 HME1 24H-QL-XII-47-1.11	9/152	4.41×10^{-6}	8.07×10^{-5}
LJP006 LNCAP 3H-QL-XII-47-1.11	5/32	5.70×10^{-6}	1.00×10^{-4}
LJP006 HCC515 24H-QL-XII-47-10	8/123	7.63×10^{-6}	1.28×10^{-4}
LJP006 MDAMB231 24H-QL-XII-47-3.33	6/60	9.30×10^{-6}	1.52×10^{-4}
LJP006 LNCAP 24H-QL-XII-47-1.11	9/170	1.09×10^{-5}	1.75×10^{-4}
LJP006 HEPG2 24H-QL-XII-47-1.11	10/219	1.29×10^{-5}	2.02×10^{-4}
LJP006 MCF7 24H-QL-XII-47-0.12	5/38	1.37×10^{-5}	2.11×10^{-4}
LJP006 LNCAP 3H-QL-XII-47-10	5/41	2.00×10^{-5}	2.92×10^{-4}
LJP006 HME1 24H-QL-XII-47-10	7/113	3.93×10^{-5}	5.18×10^{-4}
LJP006 HME1 24H-QL-XII-47-0.37	8/157	4.48×10^{-5}	5.77×10^{-4}
LJP006 LNCAP 3H-QL-XII-47-3.33	4/27	6.45×10^{-5}	7.86×10^{-4}
LJP006 SKBR3 24H-QL-XII-47-10	8/167	6.93×10^{-5}	8.34×10^{-4}
LJP006 HME1 24H-QL-XII-47-3.33	7/125	7.48×10^{-5}	8.86×10^{-4}
LJP006 HEPG2 24H-QL-XII-47-0.12	6/90	9.46×10^{-5}	1.07×10^{-3}
LJP006 HME1 3H-QL-XII-47-0.37	5/58	1.09×10^{-4}	1.21×10^{-3}
LJP006 MCF7 24H-QL-XII-47-0.37	5/58	1.09×10^{-4}	1.21×10^{-3}
LJP006 MCF7 24H-QL-XII-47-1.11	6/94	1.20×10^{-4}	1.31×10^{-3}
LJP006 LNCAP 3H-QL-XII-47-0.12	4/32	1.28×10^{-4}	1.39×10^{-3}
LJP006 BT20 3H-QL-XII-47-0.37	4/33	1.45×10^{-4}	1.53×10^{-3}
LJP006 HS578T 3H-QL-XII-47-1.11	4/41	3.40×10^{-4}	3.17×10^{-3}
LJP006 LNCAP 24H-QL-XII-47-0.37	6/115	3.62×10^{-4}	3.33×10^{-3}
LJP006 SKBR3 24H-QL-XII-47-0.12	4/60	1.45×10^{-3}	1.07×10^{-2}
LJP006 HS578T 3H-QL-XII-47-3.33	3/30	1.84×10^{-3}	1.30×10^{-2}
LJP006 MDAMB231 24H-QL-XII-47-10	4/65	1.96×10^{-3}	1.37×10^{-2}
LJP006 SKBR3 3H-QL-XII-47-1.11	4/72	2.84×10^{-3}	1.86×10^{-2}
LJP006 MCF7 24H-QL-XII-47-10	5/124	3.49×10^{-3}	2.19×10^{-2}
LJP006 BT20 24H-QL-XII-47-0.12	5/141	6.01×10^{-3}	3.38×10^{-2}
LJP006 BT20 3H-QL-XII-47-0.04	2/15	6.47×10^{-3}	3.57×10^{-2}
LJP006 A549 24H-QL-XII-47-0.37	4/98	8.49×10^{-3}	4.43×10^{-2}
LJP006 MCF10A 24H-QL-XII-47-1.11	3/53	9.24×10^{-3}	4.75×10^{-2}
LJP006 SKBR3 24H-QL-XII-47-0.37	4/101	9.42×10^{-3}	4.81×10^{-2}
