# Supplementary material for: A new advanced in silico drug discovery method for novel coronavirus (SARS-CoV-2) with tensor decomposition-based unsupervised feature extraction
Source: PLoS One. 2020 Sep 11;15(9):e0238907. doi: 10.1371/journal.pone.0238907 (PMC7485840; doi:10.1371/journal.pone.0238907)
Supplement: S24 Table — Geldanamycin significantly affects the expression of the selected 163 genes as evident in the “LINCS L1000 Chem Pert up” category in Enrichr. The last number after the—is dose density. (PDF) [file pone.0238907.s024.pdf]

S24 Table: Geldanamycin significantly affects the expression of the selected 163 genes as evident in the "LINCS L1000 Chem Pert up" category in Enrichr. The last number after the - is dose density.

| Term | Overlap | P-value | Adjusted P-value |
|---|---|---|---|
| LINCS L1000 Chem Pert up | | | |
| LJP006 LNCAP 3H-geldanamycin-0.12 | 9/34 | $5.59 \times 10^{-12}$ | $1.10 \times 10^{-9}$ |
| LJP006 HS578T 3H-geldanamycin-10 | 10/63 | $8.63 \times 10^{-11}$ | $1.01 \times 10^{-8}$ |
| LJP005 PC3 24H-geldanamycin-1.11 | 11/86 | $1.08 \times 10^{-10}$ | $1.22 \times 10^{-8}$ |
| LJP006 MCF7 24H-geldanamycin-0.12 | 10/73 | $3.91 \times 10^{-10}$ | $3.44 \times 10^{-8}$ |
| LJP006 PC3 24H-geldanamycin-0.37 | 8/45 | $2.73 \times 10^{-9}$ | $1.69 \times 10^{-7}$ |
| LJP005 BT20 3H-geldanamycin-10 | 9/65 | $2.75 \times 10^{-9}$ | $1.69 \times 10^{-7}$ |
| LJP006 HME1 24H-geldanamycin-1.11 | 13/185 | $4.00 \times 10^{-9}$ | $2.31 \times 10^{-7}$ |
| LJP006 MCF10A 24H-geldanamycin-10 | 12/157 | $6.28 \times 10^{-9}$ | $3.46 \times 10^{-7}$ |
| LJP005 MCF7 3H-geldanamycin-0.37 | 9/72 | $6.97 \times 10^{-9}$ | $3.80 \times 10^{-7}$ |
| LJP006 HME1 3H-geldanamycin-3.33 | 9/76 | $1.13 \times 10^{-8}$ | $5.70 \times 10^{-7}$ |
| LJP005 MCF7 3H-geldanamycin-1.11 | 8/56 | $1.67 \times 10^{-8}$ | $7.88 \times 10^{-7}$ |
| LJP005 HS578T 3H-geldanamycin-0.12 | 7/41 | $3.74 \times 10^{-8}$ | $1.56 \times 10^{-6}$ |
| LJP006 HME1 3H-geldanamycin-1.11 | 9/90 | $5.11 \times 10^{-8}$ | $2.04 \times 10^{-6}$ |
| LJP005 PC3 24H-geldanamycin-3.33 | 9/95 | $8.20 \times 10^{-8}$ | $3.04 \times 10^{-6}$ |
| LJP005 HS578T 3H-geldanamycin-3.33 | 8/78 | $2.37 \times 10^{-7}$ | $7.37 \times 10^{-6}$ |
| LJP006 BT20 3H-geldanamycin-10 | 7/54 | $2.69 \times 10^{-7}$ | $8.26 \times 10^{-6}$ |
| LJP006 MCF10A 3H-geldanamycin-10 | 9/113 | $3.68 \times 10^{-7}$ | $1.07 \times 10^{-5}$ |
| LJP006 HS578T 3H-geldanamycin-3.33 | 8/84 | $4.22 \times 10^{-7}$ | $1.20 \times 10^{-5}$ |
| LJP005 HS578T 3H-geldanamycin-0.37 | 7/58 | $4.45 \times 10^{-7}$ | $1.25 \times 10^{-5}$ |
| LJP006 LNCAP 3H-geldanamycin-1.11 | 7/59 | $5.02 \times 10^{-7}$ | $1.39 \times 10^{-5}$ |
| LJP005 MCF10A 3H-geldanamycin-10 | 8/86 | $5.07 \times 10^{-7}$ | $1.40 \times 10^{-5}$ |
| LJP005 MDAMB231 3H-geldanamycin-10 | 8/88 | $6.06 \times 10^{-7}$ | $1.63 \times 10^{-5}$ |
| LJP005 MCF7 24H-geldanamycin-3.33 | 10/158 | $6.96 \times 10^{-7}$ | $1.84 \times 10^{-5}$ |
| LJP006 MCF7 24H-geldanamycin-0.37 | 9/125 | $8.67 \times 10^{-7}$ | $2.20 \times 10^{-5}$ |
| LJP006 LNCAP 24H-geldanamycin-1.11 | 8/97 | $1.28 \times 10^{-6}$ | $3.02 \times 10^{-5}$ |
| LJP006 MCF7 24H-geldanamycin-1.11 | 9/135 | $1.65 \times 10^{-6}$ | $3.74 \times 10^{-5}$ |
| LJP005 MDAMB231 3H-geldanamycin-0.37 | 6/47 | $2.18 \times 10^{-6}$ | $4.69 \times 10^{-5}$ |
| LJP006 MDAMB231 3H-geldanamycin-0.12 | 6/47 | $2.18 \times 10^{-6}$ | $4.68 \times 10^{-5}$ |
| LJP006 MCF10A 3H-geldanamycin-0.37 | 8/107 | $2.69 \times 10^{-6}$ | $5.62 \times 10^{-5}$ |
| LJP006 HME1 3H-geldanamycin-0.37 | 7/77 | $3.13 \times 10^{-6}$ | $6.34 \times 10^{-5}$ |
| LJP006 HME1 3H-geldanamycin-10 | 7/78 | $3.41 \times 10^{-6}$ | $6.81 \times 10^{-5}$ |
| LJP005 BT20 3H-geldanamycin-0.04 | 5/31 | $4.84 \times 10^{-6}$ | $9.18 \times 10^{-5}$ |
| LJP006 BT20 24H-geldanamycin-0.37 | 7/83 | $5.18 \times 10^{-6}$ | $9.69 \times 10^{-5}$ |
| LJP005 A549 24H-geldanamycin-3.33 | 8/119 | $5.97 \times 10^{-6}$ | $1.10 \times 10^{-4}$ |
| LJP005 SKBR3 3H-geldanamycin-0.37 | 6/56 | $6.19 \times 10^{-6}$ | $1.13 \times 10^{-4}$ |
| LJP005 SKBR3 3H-geldanamycin-10 | 6/56 | $6.19 \times 10^{-6}$ | $1.13 \times 10^{-4}$ |
| LJP005 MCF10A 24H-geldanamycin-10 | 8/121 | $6.76 \times 10^{-6}$ | $1.21 \times 10^{-4}$ |
| LJP005 MCF7 24H-geldanamycin-10 | 6/57 | $6.88 \times 10^{-6}$ | $1.23 \times 10^{-4}$ |
| LJP006 MCF7 3H-geldanamycin-0.37 | 6/57 | $6.88 \times 10^{-6}$ | $1.23 \times 10^{-4}$ |
| LJP005 MCF7 24H-geldanamycin-1.11 | 8/122 | $7.18 \times 10^{-6}$ | $1.27 \times 10^{-4}$ |
| LJP005 HS578T 24H-geldanamycin-0.12 | 7/88 | $7.66 \times 10^{-6}$ | $1.33 \times 10^{-4}$ |
| LJP005 HS578T 3H-geldanamycin-0.04 | 5/34 | $7.77 \times 10^{-6}$ | $1.35 \times 10^{-4}$ |
| LJP005 SKBR3 3H-geldanamycin-0.12 | 5/34 | $7.77 \times 10^{-6}$ | $1.34 \times 10^{-4}$ |
| LJP006 A549 24H-geldanamycin-10 | 8/125 | $8.59 \times 10^{-6}$ | $1.47 \times 10^{-4}$ |
| LJP005 HS578T 3H-geldanamycin-1.11 | 6/60 | $9.30 \times 10^{-6}$ | $1.57 \times 10^{-4}$ |
| LJP006 LNCAP 24H-geldanamycin-3.33 | 9/168 | $9.95 \times 10^{-6}$ | $1.66 \times 10^{-4}$ |
| LJP006 MDAMB231 3H-geldanamycin-3.33 | 6/61 | $1.02 \times 10^{-5}$ | $1.69 \times 10^{-4}$ |
| LJP006 MCF10A 24H-geldanamycin-3.33 | 9/175 | $1.38 \times 10^{-5}$ | $2.16 \times 10^{-4}$ |
| LJP006 BT20 24H-geldanamycin-10 | 8/134 | $1.43 \times 10^{-5}$ | $2.24 \times 10^{-4}$ |
| LJP005 MCF7 24H-geldanamycin-0.37 | 6/65 | $1.48 \times 10^{-5}$ | $2.31 \times 10^{-4}$ |
| LJP005 BT20 3H-geldanamycin-3.33 | 5/39 | $1.56 \times 10^{-5}$ | $2.40 \times 10^{-4}$ |
| CPC015 A375 6H-geldanamycin-10.0 | 8/139 | $1.87 \times 10^{-5}$ | $2.79 \times 10^{-4}$ |
| LJP006 HS578T 24H-geldanamycin-0.37 | 8/141 | $2.07 \times 10^{-5}$ | $3.04 \times 10^{-4}$ |
| LJP005 HS578T 3H-geldanamycin-10 | 6/70 | $2.28 \times 10^{-5}$ | $3.31 \times 10^{-4}$ |

| | | | |
|---|---|---|---|
| LJP006 MCF10A 24H-geldanamycin-0.37 | 9/191 | $2.76 \times 10^{-5}$ | $3.91 \times 10^{-4}$ |
| LJP006 SKBR3 3H-geldanamycin-1.11 | 5/45 | $3.17 \times 10^{-5}$ | $4.41 \times 10^{-4}$ |
| LJP006 MDAMB231 24H-geldanamycin-3.33 | 7/112 | $3.71 \times 10^{-5}$ | $5.03 \times 10^{-4}$ |
| LJP005 MCF10A 24H-geldanamycin-0.12 | 9/202 | $4.29 \times 10^{-5}$ | $5.69 \times 10^{-4}$ |
| LJP006 LNCAP 3H-geldanamycin-0.37 | 5/48 | $4.36 \times 10^{-5}$ | $5.77 \times 10^{-4}$ |
| LJP006 MDAMB231 24H-geldanamycin-0.37 | 6/79 | $4.54 \times 10^{-5}$ | $5.96 \times 10^{-4}$ |
| LJP006 HME1 24H-geldanamycin-0.12 | 9/204 | $4.63 \times 10^{-5}$ | $6.05 \times 10^{-4}$ |
| CPC011 HT29 6H-geldanamycin-10.0 | 6/83 | $6.01 \times 10^{-5}$ | $7.52 \times 10^{-4}$ |
| LJP006 MDAMB231 3H-geldanamycin-10 | 5/52 | $6.45 \times 10^{-5}$ | $7.90 \times 10^{-4}$ |
| LJP006 HT29 24H-geldanamycin-3.33 | 6/85 | $6.87 \times 10^{-5}$ | $8.37 \times 10^{-4}$ |
| LJP006 BT20 24H-geldanamycin-0.12 | 6/86 | $7.33 \times 10^{-5}$ | $8.85 \times 10^{-4}$ |
| LJP006 MCF10A 3H-geldanamycin-0.04 | 5/54 | $7.75 \times 10^{-5}$ | $9.26 \times 10^{-4}$ |
| LJP005 HS578T 24H-geldanamycin-1.11 | 7/128 | $8.69 \times 10^{-5}$ | $1.02 \times 10^{-3}$ |
| LJP006 MDAMB231 3H-geldanamycin-1.11 | 5/57 | $1.01 \times 10^{-4}$ | $1.15 \times 10^{-3}$ |
| CPC006 DV90 6H-geldanamycin-10.0 | 6/91 | $1.01 \times 10^{-4}$ | $1.15 \times 10^{-3}$ |
| LJP006 MDAMB231 24H-geldanamycin-1.11 | 6/91 | $1.01 \times 10^{-4}$ | $1.15 \times 10^{-3}$ |
| LJP006 HME1 24H-geldanamycin-10 | 8/177 | $1.04 \times 10^{-4}$ | $1.18 \times 10^{-3}$ |
| LJP005 SKBR3 24H-geldanamycin-1.11 | 6/93 | $1.13 \times 10^{-4}$ | $1.27 \times 10^{-3}$ |
| LJP006 MCF10A 3H-geldanamycin-1.11 | 6/95 | $1.28 \times 10^{-4}$ | $1.41 \times 10^{-3}$ |
| LJP006 HME1 3H-geldanamycin-0.04 | 4/32 | $1.28 \times 10^{-4}$ | $1.41 \times 10^{-3}$ |
| LJP005 HCC515 24H-geldanamycin-3.33 | 6/99 | $1.60 \times 10^{-4}$ | $1.70 \times 10^{-3}$ |
| LJP005 BT20 24H-geldanamycin-1.11 | 5/68 | $2.33 \times 10^{-4}$ | $2.31 \times 10^{-3}$ |
| LJP006 BT20 24H-geldanamycin-1.11 | 5/68 | $2.33 \times 10^{-4}$ | $2.30 \times 10^{-3}$ |
| LJP006 MCF10A 3H-geldanamycin-3.33 | 6/108 | $2.58 \times 10^{-4}$ | $2.51 \times 10^{-3}$ |
| LJP005 HS578T 24H-geldanamycin-0.37 | 6/109 | $2.71 \times 10^{-4}$ | $2.63 \times 10^{-3}$ |
| LJP006 HCC515 24H-geldanamycin-1.11 | 6/109 | $2.71 \times 10^{-4}$ | $2.63 \times 10^{-3}$ |
| LJP005 MCF10A 24H-geldanamycin-0.37 | 8/205 | $2.84 \times 10^{-4}$ | $2.74 \times 10^{-3}$ |
| LJP006 A549 24H-geldanamycin-3.33 | 6/110 | $2.85 \times 10^{-4}$ | $2.74 \times 10^{-3}$ |
| LJP006 PC3 24H-geldanamycin-10 | 5/73 | $3.25 \times 10^{-4}$ | $3.06 \times 10^{-3}$ |
| LJP005 PC3 24H-geldanamycin-10 | 4/41 | $3.40 \times 10^{-4}$ | $3.19 \times 10^{-3}$ |
| LJP005 BT20 24H-geldanamycin-10 | 5/74 | $3.46 \times 10^{-4}$ | $3.22 \times 10^{-3}$ |
| LJP005 MCF10A 3H-geldanamycin-0.12 | 5/74 | $3.46 \times 10^{-4}$ | $3.22 \times 10^{-3}$ |
| LJP005 HEPG2 24H-geldanamycin-1.11 | 6/115 | $3.62 \times 10^{-4}$ | $3.35 \times 10^{-3}$ |
| LJP005 MCF10A 3H-geldanamycin-3.33 | 6/117 | $3.97 \times 10^{-4}$ | $3.63 \times 10^{-3}$ |
| LJP006 MCF7 3H-geldanamycin-10 | 4/43 | $4.10 \times 10^{-4}$ | $3.72 \times 10^{-3}$ |
| CPC019 VCAP 6H-geldanamycin-10.0 | 6/120 | $4.54 \times 10^{-4}$ | $4.05 \times 10^{-3}$ |
| LJP006 HME1 24H-geldanamycin-3.33 | 7/168 | $4.63 \times 10^{-4}$ | $4.12 \times 10^{-3}$ |
| LJP005 HCC515 24H-geldanamycin-1.11 | 5/81 | $5.26 \times 10^{-4}$ | $4.60 \times 10^{-3}$ |
| LJP006 SKBR3 3H-geldanamycin-0.04 | 4/46 | $5.31 \times 10^{-4}$ | $4.62 \times 10^{-3}$ |
| LJP006 LNCAP 24H-geldanamycin-10 | 6/125 | $5.64 \times 10^{-4}$ | $4.86 \times 10^{-3}$ |
| LJP006 HME1 3H-geldanamycin-0.12 | 4/47 | $5.77 \times 10^{-4}$ | $4.96 \times 10^{-3}$ |
| LJP006 MDAMB231 24H-geldanamycin-0.12 | 5/84 | $6.21 \times 10^{-4}$ | $5.28 \times 10^{-3}$ |
| LJP005 HA1E 24H-geldanamycin-0.12 | 4/48 | $6.26 \times 10^{-4}$ | $5.31 \times 10^{-3}$ |
| CPC020 HA1E 6H-geldanamycin-10.0 | 5/85 | $6.56 \times 10^{-4}$ | $5.53 \times 10^{-3}$ |
| LJP005 HS578T 24H-geldanamycin-3.33 | 5/85 | $6.56 \times 10^{-4}$ | $5.52 \times 10^{-3}$ |
| LJP006 HCC515 24H-geldanamycin-0.12 | 4/49 | $6.77 \times 10^{-4}$ | $5.68 \times 10^{-3}$ |
| CPC009 A549 6H-geldanamycin-10.0 | 5/87 | $7.29 \times 10^{-4}$ | $6.06 \times 10^{-3}$ |
| LJP006 HS578T 24H-geldanamycin-0.12 | 5/89 | $8.09 \times 10^{-4}$ | $6.56 \times 10^{-3}$ |
| LJP006 HT29 24H-geldanamycin-1.11 | 5/91 | $8.94 \times 10^{-4}$ | $7.15 \times 10^{-3}$ |
| LJP005 MCF10A 24H-geldanamycin-3.33 | 7/188 | $9.01 \times 10^{-4}$ | $7.19 \times 10^{-3}$ |
| CPC016 A375 6H-geldanamycin-10.0 | 4/53 | $9.12 \times 10^{-4}$ | $7.27 \times 10^{-3}$ |
| LJP005 MCF7 3H-geldanamycin-10 | 4/54 | $9.79 \times 10^{-4}$ | $7.69 \times 10^{-3}$ |
| LJP006 MDAMB231 24H-geldanamycin-10 | 5/93 | $9.86 \times 10^{-4}$ | $7.71 \times 10^{-3}$ |
| CPC005 HT29 6H-geldanamycin-10.0 | 5/94 | $1.04 \times 10^{-3}$ | $8.08 \times 10^{-3}$ |
| LJP005 SKBR3 3H-geldanamycin-0.04 | 3/25 | $1.07 \times 10^{-3}$ | $8.27 \times 10^{-3}$ |
| LJP006 SKBR3 24H-geldanamycin-0.37 | 5/95 | $1.09 \times 10^{-3}$ | $8.35 \times 10^{-3}$ |
| LJP006 HME1 24H-geldanamycin-0.04 | 6/145 | $1.22 \times 10^{-3}$ | $9.20 \times 10^{-3}$ |

S24 Table: (Continued)

| | | | |
|---|---|---|---|
| LJP006 HA1E 24H-geldanamycin-0.12 | 4/58 | $1.28 \times 10^{-3}$ | $9.54 \times 10^{-3}$ |
| LJP006 PC3 24H-geldanamycin-1.11 | 4/58 | $1.28 \times 10^{-3}$ | $9.53 \times 10^{-3}$ |
| CPC010 A375 6H-geldanamycin-10.0 | 5/99 | $1.31 \times 10^{-3}$ | $9.70 \times 10^{-3}$ |
| CPC006 NOMO1 6H-geldanamycin-10.0 | 5/100 | $1.37 \times 10^{-3}$ | $1.01 \times 10^{-2}$ |
| LJP006 LNCAP 3H-geldanamycin-3.33 | 4/59 | $1.37 \times 10^{-3}$ | $1.00 \times 10^{-2}$ |
| LJP006 HS578T 24H-geldanamycin-10 | 6/154 | $1.66 \times 10^{-3}$ | $1.18 \times 10^{-2}$ |
| LJP006 HT29 24H-geldanamycin-10 | 5/105 | $1.70 \times 10^{-3}$ | $1.20 \times 10^{-2}$ |
| CPC019 HCC515 6H-geldanamycin-10.0 | 5/106 | $1.77 \times 10^{-3}$ | $1.24 \times 10^{-2}$ |
| LJP005 MDAMB231 3H-geldanamycin-1.11 | 4/64 | $1.85 \times 10^{-3}$ | $1.29 \times 10^{-2}$ |
| LJP006 HCC515 24H-geldanamycin-10 | 4/64 | $1.85 \times 10^{-3}$ | $1.29 \times 10^{-2}$ |
| CPC006 SW620 6H-geldanamycin-10.0 | 4/65 | $1.96 \times 10^{-3}$ | $1.36 \times 10^{-2}$ |
| LJP005 SKBR3 3H-geldanamycin-3.33 | 4/67 | $2.19 \times 10^{-3}$ | $1.48 \times 10^{-2}$ |
| LJP006 HME1 24H-geldanamycin-0.37 | 6/163 | $2.22 \times 10^{-3}$ | $1.49 \times 10^{-2}$ |
| LJP005 A375 24H-geldanamycin-3.33 | 5/112 | $2.25 \times 10^{-3}$ | $1.52 \times 10^{-2}$ |
| LJP006 MCF7 24H-geldanamycin-3.33 | 4/70 | $2.57 \times 10^{-3}$ | $1.70 \times 10^{-2}$ |
| LJP006 SKBR3 24H-geldanamycin-0.04 | 4/72 | $2.84 \times 10^{-3}$ | $1.84 \times 10^{-2}$ |
| LJP006 MCF7 3H-geldanamycin-1.11 | 3/37 | $3.37 \times 10^{-3}$ | $2.11 \times 10^{-2}$ |
| LJP006 MCF10A 24H-geldanamycin-0.12 | 6/182 | $3.82 \times 10^{-3}$ | $2.33 \times 10^{-2}$ |
| CPC006 VCAP 6H-geldanamycin-10.0 | 7/244 | $3.93 \times 10^{-3}$ | $2.38 \times 10^{-2}$ |
| CPC004 VCAP 6H-geldanamycin-10.0 | 4/80 | $4.16 \times 10^{-3}$ | $2.50 \times 10^{-2}$ |
| LJP006 HT29 24H-geldanamycin-0.37 | 3/40 | $4.21 \times 10^{-3}$ | $2.50 \times 10^{-2}$ |
| CPC001 PC3 6H-geldanamycin-10.0 | 5/130 | $4.27 \times 10^{-3}$ | $2.54 \times 10^{-2}$ |
| LJP006 SKBR3 3H-geldanamycin-3.33 | 3/41 | $4.51 \times 10^{-3}$ | $2.65 \times 10^{-2}$ |
| LJP005 SKBR3 24H-geldanamycin-0.12 | 4/82 | $4.54 \times 10^{-3}$ | $2.66 \times 10^{-2}$ |
| LJP006 HCC515 24H-geldanamycin-3.33 | 4/84 | $4.95 \times 10^{-3}$ | $2.84 \times 10^{-2}$ |
| CPC008 A549 6H-geldanamycin-10.0 | 5/137 | $5.33 \times 10^{-3}$ | $3.02 \times 10^{-2}$ |
| LJP006 SKBR3 3H-geldanamycin-0.37 | 3/44 | $5.51 \times 10^{-3}$ | $3.08 \times 10^{-2}$ |
| CPC004 HT29 6H-geldanamycin-10.0 | 4/87 | $5.60 \times 10^{-3}$ | $3.13 \times 10^{-2}$ |
| LJP006 BT20 24H-geldanamycin-3.33 | 4/88 | $5.83 \times 10^{-3}$ | $3.24 \times 10^{-2}$ |
| LJP005 SKBR3 24H-geldanamycin-0.04 | 3/46 | $6.24 \times 10^{-3}$ | $3.40 \times 10^{-2}$ |
| LJP006 MCF7 24H-geldanamycin-10 | 3/46 | $6.24 \times 10^{-3}$ | $3.40 \times 10^{-2}$ |
| CPC006 SKLU1 6H-geldanamycin-10.0 | 4/90 | $6.31 \times 10^{-3}$ | $3.43 \times 10^{-2}$ |
| LJP005 MCF10A 3H-geldanamycin-0.37 | 4/90 | $6.31 \times 10^{-3}$ | $3.43 \times 10^{-2}$ |
| LJP006 HA1E 24H-geldanamycin-10 | 4/90 | $6.31 \times 10^{-3}$ | $3.43 \times 10^{-2}$ |
| LJP006 SKBR3 3H-geldanamycin-10 | 3/49 | $7.44 \times 10^{-3}$ | $3.91 \times 10^{-2}$ |
| LJP006 A549 24H-geldanamycin-1.11 | 4/95 | $7.62 \times 10^{-3}$ | $3.99 \times 10^{-2}$ |
| LJP005 MCF10A 3H-geldanamycin-0.04 | 3/50 | $7.87 \times 10^{-3}$ | $4.10 \times 10^{-2}$ |
| LJP005 A549 24H-geldanamycin-1.11 | 4/99 | $8.79 \times 10^{-3}$ | $4.45 \times 10^{-2}$ |
| CPC019 HA1E 6H-geldanamycin-10.0 | 4/100 | $9.10 \times 10^{-3}$ | $4.58 \times 10^{-2}$ |
| LJP006 LNCAP 24H-geldanamycin-0.37 | 4/101 | $9.42 \times 10^{-3}$ | $4.68 \times 10^{-2}$ |
