# Supplementary material for: A new advanced in silico drug discovery method for novel coronavirus (SARS-CoV-2) with tensor decomposition-based unsupervised feature extraction
Source: PLoS One. 2020 Sep 11;15(9):e0238907. doi: 10.1371/journal.pone.0238907 (PMC7485840; doi:10.1371/journal.pone.0238907)
Supplement: S25 Table — Geldanamycin significantly affects the expression of the selected 163 genes as evident in the “LINCS L1000 Chem Pert down” category in Enrichr. The last number after the—is dose density. (PDF) [file pone.0238907.s025.pdf]

S25 Table: Geldanamycin significantly affects the expression of the selected 163 genes as evident in the "LINCS L1000 Chem Pert down" category in Enrichr. The last number after the - is dose density.

Term	Overlap	P-value	Adjusted P-value
LINCS L1000 Chem Pert down			
LJP005 HA1E 24H-geldanamycin-3.33	18/148	2.41×10^{-16}	3.06×10^{-13}
LJP005 HS578T 24H-geldanamycin-10	16/131	1.11×10^{-14}	6.92×10^{-12}
LJP006 SKBR3 24H-geldanamycin-0.37	14/91	2.01×10^{-14}	1.07×10^{-11}
LJP006 HA1E 24H-geldanamycin-3.33	16/138	2.55×10^{-14}	1.26×10^{-11}
LJP005 HS578T 24H-geldanamycin-3.33	15/118	4.24×10^{-14}	1.98×10^{-11}
LJP006 HA1E 24H-geldanamycin-0.37	15/121	6.20×10^{-14}	2.53×10^{-11}
LJP006 HA1E 24H-geldanamycin-1.11	16/157	1.97×10^{-13}	6.87×10^{-11}
LJP006 MDAMB231 24H-geldanamycin-1.11	14/123	1.45×10^{-12}	3.54×10^{-10}
LJP006 SKBR3 24H-geldanamycin-0.12	12/78	1.56×10^{-12}	3.71×10^{-10}
LJP005 A375 24H-geldanamycin-3.33	16/183	2.14×10^{-12}	4.73×10^{-10}
LJP005 HA1E 24H-geldanamycin-10	14/137	6.46×10^{-12}	1.14×10^{-9}
LJP006 A375 24H-geldanamycin-0.37	15/166	6.69×10^{-12}	1.16×10^{-9}
LJP005 HA1E 24H-geldanamycin-1.11	14/142	1.06×10^{-11}	1.68×10^{-9}
LJP005 SKBR3 24H-geldanamycin-0.37	11/82	6.38×10^{-11}	7.41×10^{-9}
LJP005 SKBR3 24H-geldanamycin-1.11	10/63	8.63×10^{-11}	9.22×10^{-9}
LJP005 HS578T 24H-geldanamycin-1.11	12/109	9.04×10^{-11}	9.60×10^{-9}
LJP005 BT20 24H-geldanamycin-0.12	10/64	1.02×10^{-10}	1.05×10^{-8}
LJP005 HA1E 24H-geldanamycin-0.37	13/141	1.39×10^{-10}	1.37×10^{-8}
LJP006 A375 24H-geldanamycin-1.11	14/182	3.00×10^{-10}	2.60×10^{-8}
LJP006 MDAMB231 24H-geldanamycin-0.37	11/96	3.64×10^{-10}	3.05×10^{-8}
LJP006 BT20 24H-geldanamycin-1.11	10/75	5.15×10^{-10}	4.04×10^{-8}
LJP005 HEPG2 24H-geldanamycin-3.33	13/159	6.23×10^{-10}	4.77×10^{-8}
LJP006 HS578T 24H-geldanamycin-10	12/132	8.57×10^{-10}	6.20×10^{-8}
LJP005 MCF10A 3H-geldanamycin-0.04	9/61	1.53×10^{-9}	9.92×10^{-8}
LJP006 SKBR3 24H-geldanamycin-0.04	10/89	2.86×10^{-9}	1.68×10^{-7}
LJP006 HS578T 24H-geldanamycin-0.37	12/148	3.20×10^{-9}	1.85×10^{-7}
LJP006 SKBR3 24H-geldanamycin-1.11	8/50	6.57×10^{-9}	3.39×10^{-7}
LJP006 HEPG2 24H-geldanamycin-3.33	10/98	7.41×10^{-9}	3.75×10^{-7}
LJP006 BT20 24H-geldanamycin-0.12	9/74	8.93×10^{-9}	4.44×10^{-7}
LJP005 SKBR3 24H-geldanamycin-0.04	9/78	1.43×10^{-8}	6.49×10^{-7}
LJP005 A375 24H-geldanamycin-0.04	9/81	2.01×10^{-8}	8.55×10^{-7}
LJP006 HS578T 24H-geldanamycin-1.11	11/145	2.97×10^{-8}	1.18×10^{-6}
LJP006 HA1E 24H-geldanamycin-10	9/86	3.42×10^{-8}	1.34×10^{-6}
LJP006 SKBR3 24H-geldanamycin-3.33	9/86	3.42×10^{-8}	1.33×10^{-6}
LJP006 HME1 24H-geldanamycin-0.04	10/121	5.70×10^{-8}	2.08×10^{-6}
LJP006 HS578T 24H-geldanamycin-3.33	10/122	6.17×10^{-8}	2.24×10^{-6}
LJP006 MCF10A 3H-geldanamycin-0.37	8/66	6.30×10^{-8}	2.25×10^{-6}
LJP005 MDAMB231 24H-geldanamycin-3.33	7/46	8.60×10^{-8}	2.95×10^{-6}
LJP006 A549 24H-geldanamycin-0.37	6/28	8.68×10^{-8}	2.96×10^{-6}
LJP006 HS578T 24H-geldanamycin-0.12	9/97	9.84×10^{-8}	3.31×10^{-6}
LJP006 HME1 24H-geldanamycin-3.33	11/164	1.05×10^{-7}	3.51×10^{-6}
LJP006 A375 24H-geldanamycin-0.04	9/104	1.80×10^{-7}	5.55×10^{-6}
LJP005 SKBR3 24H-geldanamycin-3.33	8/78	2.37×10^{-7}	6.98×10^{-6}
LJP006 MCF10A 3H-geldanamycin-3.33	8/80	2.89×10^{-7}	8.17×10^{-6}
LJP006 MDAMB231 24H-geldanamycin-3.33	8/84	4.22×10^{-7}	1.12×10^{-5}
LJP005 MCF10A 3H-geldanamycin-0.37	8/85	4.63×10^{-7}	1.20×10^{-5}
LJP005 MCF10A 3H-geldanamycin-0.12	8/86	5.07×10^{-7}	1.29×10^{-5}
LJP006 HME1 24H-geldanamycin-10	10/153	5.17×10^{-7}	1.31×10^{-5}
LJP006 HEPG2 24H-geldanamycin-1.11	8/88	6.06×10^{-7}	1.50×10^{-5}
LJP005 HS578T 24H-geldanamycin-0.12	8/89	6.61×10^{-7}	1.62×10^{-5}
LJP006 MDAMB231 24H-geldanamycin-0.12	8/89	6.61×10^{-7}	1.61×10^{-5}
LJP006 MCF10A 3H-geldanamycin-0.04	7/62	7.08×10^{-7}	1.72×10^{-5}
LJP006 BT20 24H-geldanamycin-3.33	8/94	1.01×10^{-6}	2.32×10^{-5}

S25 Table: (Continued)

LJP006 MDAMB231 24H-geldanamycin-10	8/94	1.01×10^{-6}	2.32×10^{-5}
LJP005 BT20 24H-geldanamycin-0.37	8/96	1.18×10^{-6}	2.67×10^{-5}
LJP006 PC3 24H-geldanamycin-10	8/96	1.18×10^{-6}	2.67×10^{-5}
LJP005 BT20 24H-geldanamycin-1.11	7/67	1.21×10^{-6}	2.73×10^{-5}
LJP006 MCF10A 3H-geldanamycin-0.12	7/67	1.21×10^{-6}	2.72×10^{-5}
LJP005 HEPG2 24H-geldanamycin-1.11	9/131	1.29×10^{-6}	2.85×10^{-5}
LJP006 A375 24H-geldanamycin-3.33	9/131	1.29×10^{-6}	2.85×10^{-5}
LJP005 MCF10A 3H-geldanamycin-3.33	7/68	1.34×10^{-6}	2.96×10^{-5}
LJP005 MCF7 3H-geldanamycin-10	6/45	1.67×10^{-6}	3.56×10^{-5}
LJP006 HA1E 24H-geldanamycin-0.12	6/45	1.67×10^{-6}	3.56×10^{-5}
LJP005 HCC515 24H-geldanamycin-3.33	9/140	2.24×10^{-6}	4.54×10^{-5}
LJP006 HCC515 24H-geldanamycin-1.11	9/144	2.83×10^{-6}	5.51×10^{-5}
LJP006 LNCAP 24H-geldanamycin-3.33	9/163	7.79×10^{-6}	1.30×10^{-4}
LJP006 HME1 24H-geldanamycin-0.12	9/164	8.19×10^{-6}	1.37×10^{-4}
LJP006 MCF10A 3H-geldanamycin-1.11	7/89	8.26×10^{-6}	1.37×10^{-4}
LJP006 PC3 24H-geldanamycin-3.33	7/90	8.89×10^{-6}	1.46×10^{-4}
LJP005 SKBR3 24H-geldanamycin-10	7/92	1.03×10^{-5}	1.66×10^{-4}
LJP005 HS578T 3H-geldanamycin-0.37	5/36	1.04×10^{-5}	1.67×10^{-4}
LJP006 MCF7 3H-geldanamycin-1.11	5/38	1.37×10^{-5}	2.10×10^{-4}
LJP006 BT20 24H-geldanamycin-10	7/97	1.46×10^{-5}	2.24×10^{-4}
LJP005 SKBR3 24H-geldanamycin-0.12	6/69	2.10×10^{-5}	3.05×10^{-4}
LJP006 BT20 24H-geldanamycin-0.37	6/69	2.10×10^{-5}	3.04×10^{-4}
LJP006 HME1 3H-geldanamycin-1.11	5/42	2.25×10^{-5}	3.24×10^{-4}
LJP005 MCF10A 3H-geldanamycin-10	5/43	2.53×10^{-5}	3.59×10^{-4}
LJP005 MCF10A 3H-geldanamycin-1.11	6/72	2.68×10^{-5}	3.77×10^{-4}
LJP006 HCC515 24H-geldanamycin-3.33	7/110	3.31×10^{-5}	4.51×10^{-4}
LJP006 LNCAP 24H-geldanamycin-1.11	7/110	3.31×10^{-5}	4.51×10^{-4}
LJP006 A549 24H-geldanamycin-1.11	6/76	3.65×10^{-5}	4.88×10^{-4}
LJP006 HME1 24H-geldanamycin-0.37	8/154	3.90×10^{-5}	5.18×10^{-4}
LJP006 SKBR3 24H-geldanamycin-10	6/77	3.93×10^{-5}	5.19×10^{-4}
LJP005 A549 24H-geldanamycin-1.11	6/78	4.23×10^{-5}	5.52×10^{-4}
LJP005 MDAMB231 24H-geldanamycin-0.12	6/78	4.23×10^{-5}	5.51×10^{-4}
LJP006 A375 24H-geldanamycin-0.12	8/163	5.84×10^{-5}	7.25×10^{-4}
LJP006 HS578T 3H-geldanamycin-1.11	5/52	6.45×10^{-5}	7.82×10^{-4}
LJP005 A549 24H-geldanamycin-3.33	7/123	6.75×10^{-5}	8.18×10^{-4}
LJP006 MDAMB231 3H-geldanamycin-1.11	5/53	7.07×10^{-5}	8.48×10^{-4}
LJP005 BT20 24H-geldanamycin-0.04	6/87	7.83×10^{-5}	9.20×10^{-4}
LJP006 A549 24H-geldanamycin-3.33	6/87	7.83×10^{-5}	9.19×10^{-4}
LJP005 PC3 24H-geldanamycin-3.33	6/89	8.89×10^{-5}	1.02×10^{-3}
LJP006 HEPG2 24H-geldanamycin-10	6/94	1.20×10^{-4}	1.31×10^{-3}
LJP005 HS578T 3H-geldanamycin-1.11	4/32	1.28×10^{-4}	1.39×10^{-3}
LJP006 HME1 24H-geldanamycin-1.11	7/145	1.89×10^{-4}	1.92×10^{-3}
LJP005 HS578T 3H-geldanamycin-3.33	4/36	2.04×10^{-4}	2.06×10^{-3}
LJP005 MCF10A 24H-geldanamycin-10	5/67	2.17×10^{-4}	2.17×10^{-3}
LJP006 MDAMB231 3H-geldanamycin-0.12	3/16	2.75×10^{-4}	2.66×10^{-3}
LJP006 PC3 24H-geldanamycin-1.11	5/72	3.05×10^{-4}	2.90×10^{-3}
LJP005 HS578T 3H-geldanamycin-10	4/40	3.09×10^{-4}	2.93×10^{-3}
LJP005 BT20 24H-geldanamycin-3.33	5/73	3.25×10^{-4}	3.06×10^{-3}
LJP005 HCC515 24H-geldanamycin-1.11	6/113	3.29×10^{-4}	3.09×10^{-3}
LJP006 BT20 3H-geldanamycin-10	4/44	4.48×10^{-4}	3.98×10^{-3}
LJP006 HEPG2 24H-geldanamycin-0.37	4/44	4.48×10^{-4}	3.97×10^{-3}
LJP005 MDAMB231 24H-geldanamycin-1.11	4/46	5.31×10^{-4}	4.61×10^{-3}
LJP006 SKBR3 3H-geldanamycin-0.12	3/20	5.47×10^{-4}	4.71×10^{-3}
LJP006 LNCAP 24H-geldanamycin-10	7/173	5.51×10^{-4}	4.74×10^{-3}
LJP006 HT29 24H-geldanamycin-1.11	5/85	6.56×10^{-4}	5.47×10^{-3}
LJP005 SKBR3 3H-geldanamycin-0.04	4/49	6.77×10^{-4}	5.62×10^{-3}
LJP005 HS578T 3H-geldanamycin-0.04	3/22	7.30×10^{-4}	6.01×10^{-3}

S25 Table: (Continued)

LJP006 SKBR3 3H-geldanamycin-1.11	4/51	7.89×10^{-4}	6.39×10^{-3}
LJP005 MCF10A 24H-geldanamycin-3.33	6/134	8.13×10^{-4}	6.56×10^{-3}
LJP005 BT20 24H-geldanamycin-10	4/52	8.49×10^{-4}	6.82×10^{-3}
LJP006 MCF10A 24H-geldanamycin-0.12	6/137	9.12×10^{-4}	7.26×10^{-3}
LJP005 SKBR3 3H-geldanamycin-3.33	4/53	9.12×10^{-4}	7.25×10^{-3}
LJP006 HS578T 3H-geldanamycin-3.33	4/53	9.12×10^{-4}	7.24×10^{-3}
LJP005 MDAMB231 24H-geldanamycin-0.37	4/54	9.79×10^{-4}	7.70×10^{-3}
CPC014 NPC 24H-geldanamycin-10.0	6/139	9.84×10^{-4}	7.71×10^{-3}
LJP006 HS578T 3H-geldanamycin-0.37	4/56	1.12×10^{-3}	8.60×10^{-3}
LJP006 MCF10A 24H-geldanamycin-0.37	6/144	1.18×10^{-3}	9.01×10^{-3}
LJP005 MCF10A 24H-geldanamycin-0.12	6/147	1.31×10^{-3}	9.80×10^{-3}
LJP006 MCF10A 24H-geldanamycin-0.04	5/102	1.49×10^{-3}	1.09×10^{-2}
LJP005 SKBR3 3H-geldanamycin-0.12	3/28	1.50×10^{-3}	1.09×10^{-2}
CPC018 A549 24H-geldanamycin-10.0	5/104	1.62×10^{-3}	1.17×10^{-2}
CPC009 MCF7 24H-geldanamycin-10.0	5/108	1.92×10^{-3}	1.35×10^{-2}
LJP005 MDAMB231 3H-geldanamycin-3.33	3/31	2.02×10^{-3}	1.40×10^{-2}
LJP005 MCF10A 24H-geldanamycin-1.11	5/110	2.08×10^{-3}	1.43×10^{-2}
LJP006 MCF7 24H-geldanamycin-0.04	3/33	2.42×10^{-3}	1.63×10^{-2}
LJP005 MDAMB231 3H-geldanamycin-1.11	3/34	2.64×10^{-3}	1.75×10^{-2}
LJP006 MDAMB231 3H-geldanamycin-3.33	3/34	2.64×10^{-3}	1.75×10^{-2}
LJP005 MCF10A 24H-geldanamycin-0.04	5/119	2.93×10^{-3}	1.90×10^{-2}
CPC006 SNUC4 6H-geldanamycin-10.0	4/75	3.30×10^{-3}	2.10×10^{-2}
LJP006 HT29 24H-geldanamycin-0.37	3/37	3.37×10^{-3}	2.13×10^{-2}
LJP005 MDAMB231 3H-geldanamycin-0.04	3/38	3.64×10^{-3}	2.27×10^{-2}
LJP005 MDAMB231 3H-geldanamycin-10	3/38	3.64×10^{-3}	2.27×10^{-2}
LJP006 MCF10A 24H-geldanamycin-1.11	5/129	4.14×10^{-3}	2.52×10^{-2}
LJP005 MCF7 24H-geldanamycin-1.11	5/130	4.27×10^{-3}	2.58×10^{-2}
LJP006 MCF10A 24H-geldanamycin-3.33	5/130	4.27×10^{-3}	2.58×10^{-2}
LJP006 HCC515 24H-geldanamycin-10	4/83	4.74×10^{-3}	2.81×10^{-2}
LJP006 HME1 3H-geldanamycin-0.12	3/42	4.83×10^{-3}	2.84×10^{-2}
LJP006 LNCAP 3H-geldanamycin-1.11	3/43	5.16×10^{-3}	2.99×10^{-2}
CPC006 NCIH1694 6H-geldanamycin-10.0	3/44	5.51×10^{-3}	3.17×10^{-2}
LJP006 HME1 3H-geldanamycin-0.37	3/44	5.51×10^{-3}	3.16×10^{-2}
LJP005 HS578T 24H-geldanamycin-0.37	4/88	5.83×10^{-3}	3.31×10^{-2}
CPC006 THP1 6H-geldanamycin-10.0	4/90	6.31×10^{-3}	3.51×10^{-2}
CPC013 A549 6H-geldanamycin-10.0	4/93	7.08×10^{-3}	3.84×10^{-2}
LJP005 PC3 24H-geldanamycin-1.11	4/93	7.08×10^{-3}	3.84×10^{-2}
CPC005 HT29 24H-geldanamycin-10.0	5/147	7.14×10^{-3}	3.87×10^{-2}
CPC004 A375 6H-geldanamycin-10.0	3/49	7.44×10^{-3}	4.01×10^{-2}
CPC004 VCAP 24H-geldanamycin-10.0	4/95	7.62×10^{-3}	4.07×10^{-2}
CPC016 MCF7 24H-geldanamycin-10.0	5/150	7.76×10^{-3}	4.14×10^{-2}
LJP005 HT29 24H-geldanamycin-3.33	5/150	7.76×10^{-3}	4.14×10^{-2}
CPC018 ASC 24H-geldanamycin-10.0	3/51	8.31×10^{-3}	4.37×10^{-2}
LJP006 BT20 24H-geldanamycin-0.04	3/51	8.31×10^{-3}	4.36×10^{-2}
CPC006 U937 6H-geldanamycin-10.0	4/98	8.49×10^{-3}	4.44×10^{-2}
LJP005 MDAMB231 3H-geldanamycin-0.37	3/52	8.77×10^{-3}	4.56×10^{-2}
CPC005 A549 24H-geldanamycin-10.0	5/156	9.11×10^{-3}	4.70×10^{-2}
LJP005 BT20 3H-geldanamycin-3.33	2/18	9.27×10^{-3}	4.75×10^{-2}
