# Supplementary material for: A new advanced in silico drug discovery method for novel coronavirus (SARS-CoV-2) with tensor decomposition-based unsupervised feature extraction
Source: PLoS One. 2020 Sep 11;15(9):e0238907. doi: 10.1371/journal.pone.0238907 (PMC7485840; doi:10.1371/journal.pone.0238907)
Supplement: S26 Table — Genes whose expression is altered by SARS-CoV-2-related viruses that significantly interact with the 163 genes selected by TD-based unsupervised FE and enriched by “Drug Perturbations from GEO up/down” in Enrichr. (PDF) [file pone.0238907.s026.pdf]

S26 Table: Genes whose expression is altered by SARS-CoV-2-related viruses that significantly interact with the 163 genes selected by TD-based unsupervised FE and enriched by "Drug Perturbations from GEO up/down" in Enrichr

Term	Overlap	P-value	Adjusted P-value
Drug Perturbations from GEO up			
MK-886 CID 3651377 human GSE3202 sample 3193	54/368	3.90×10^{-53}	3.53×10^{-50}
fluticasone 5311101 human GSE15823 sample 3090	53/351	8.70×10^{-53}	3.94×10^{-50}
1-Naphthyl isothiocyanate 11080 rat GSE5509 sample 3568	50/301	7.71×10^{-52}	2.33×10^{-49}
quercetin 5280343 human GSE7259 sample 3416	50/327	6.03×10^{-50}	1.36×10^{-47}
N-METHYLFORMAMIDE 31254 rat GSE5509 sample 3570	46/283	4.37×10^{-47}	7.93×10^{-45}
NICKEL 935 human GSE6907 sample 3531	46/288	1.02×10^{-46}	1.54×10^{-44}
apratoxin A 6326668 human GSE2742 sample 3070	43/246	2.30×10^{-45}	2.98×10^{-43}
quercetin 5280343 human GSE7259 sample 3415	47/336	6.05×10^{-45}	6.85×10^{-43}
sapphyrin PCI-2050 (1.25 Î¼M) 9855235 human GSE6400 sample 3101	48/367	1.71×10^{-44}	1.72×10^{-42}
rosiglitazone DB00412 human GSE7035 sample 2810	43/281	9.52×10^{-43}	8.63×10^{-41}
Drug Perturbations from GEO down			
gatifloxacin 5379 human GSE9166 sample 2626	48/266	1.61×10^{-51}	1.46×10^{-48}
atorvastatin DB01076 human GSE2450 sample 2484	46/250	1.02×10^{-49}	4.62×10^{-47}
bexarotene DB00307 human GSE12791 sample 2681	46/253	1.84×10^{-49}	5.54×10^{-47}
clinafloxacin 60063 human GSE9166 sample 2625	55/470	1.30×10^{-48}	2.95×10^{-46}
motexafin gadolinium (12 h) DB05428 human GSE2189 sample 3127	48/320	1.89×10^{-47}	3.41×10^{-45}
BPDE 41322 human GSE19510 sample 3379	47/300	2.36×10^{-47}	3.55×10^{-45}
trovafloxacin 62959 human GSE9166 sample 2629	53/459	1.98×10^{-46}	2.55×10^{-44}
HYPOCHLOROUS ACID 24341 human GSE11630 sample 3199	40/204	2.85×10^{-44}	3.21×10^{-42}
trovafloxacin DB00685 human GSE9166 sample 3036	51/451	4.05×10^{-44}	4.06×10^{-42}
doxycycline DB00254 human GSE2624 sample 3077	48/391	3.82×10^{-43}	3.45×10^{-41}
