# Supplementary material for: A new advanced in silico drug discovery method for novel coronavirus (SARS-CoV-2) with tensor decomposition-based unsupervised feature extraction
Source: PLoS One. 2020 Sep 11;15(9):e0238907. doi: 10.1371/journal.pone.0238907 (PMC7485840; doi:10.1371/journal.pone.0238907)
Supplement: S27 Table — Genes whose expression is altered by SARS-CoV-2-related viruses that significantly interact with the 163 genes selected by TD-based unsupervised FE and enriched by “Drug Matrix” in Enrichr. (PDF) [file pone.0238907.s027.pdf]

S27 Table: Genes whose expression is altered by SARS-CoV-2-related viruses that significantly interact with the 163 genes selected by TD-based unsupervised FE and enriched by "Drug Matrix" in Enrichr

Term	Overlap	P-value	Adjusted P-value
2-Amino-4-Nitrophenol-625 mg/kg in CMC-Rat-Kidney-1d-up	26/300	2.01×10^{-19}	1.59×10^{-15}
Allyl Alcohol-32 mg/kg in Saline-Rat-Liver-1d-up	25/291	1.30×10^{-18}	5.12×10^{-15}
Meloxicam-33 mg/kg in Corn Oil-Rat-Kidney-5d-up	23/261	1.96×10^{-17}	5.14×10^{-14}
Lipopolysaccharide E. Coli O55:B5-1.25 mg/kg in Saline-Rat-Kidney-1d-up	24/295	2.36×10^{-17}	4.64×10^{-14}
44'-Methylenedianiline-81 mg/kg in Corn Oil-Rat-Liver-3d-up	25/333	3.27×10^{-17}	5.16×10^{-14}
Gentamicin-40 mg/kg in Saline-Rat-Kidney-14d-up	24/309	6.83×10^{-17}	8.96×10^{-14}
Lead(IV) Acetate-600 mg/kg in Saline-Rat-Kidney-5d-up	24/309	6.83×10^{-17}	7.68×10^{-14}
Dibromochloromethane-325 mg/kg in CMC-Rat-Kidney-3d-up	24/312	8.51×10^{-17}	8.38×10^{-14}
Allopurinol-175 mg/kg in Corn Oil-Rat-Kidney-3d-up	24/329	2.84×10^{-16}	2.49×10^{-13}
Benzyl Acetate-1868 mg/kg in CMC-Rat-Kidney-3d-up	24/330	3.05×10^{-16}	2.40×10^{-13}
