# Supplementary material for: A new advanced in silico drug discovery method for novel coronavirus (SARS-CoV-2) with tensor decomposition-based unsupervised feature extraction
Source: PLoS One. 2020 Sep 11;15(9):e0238907. doi: 10.1371/journal.pone.0238907 (PMC7485840; doi:10.1371/journal.pone.0238907)
Supplement: S28 Table — List of in silico screened drugs [33] whose target genes were also enriched in the 163 genes selected by TD-based unsupervised FE. (PDF) [file pone.0238907.s028.pdf]

S28 Table: List of *in silico* screened drugs [31] whose target genes were also enriched in the 163 genes selected by TD-based unsupervised FE.

Term	Overlap	P-value	Adjusted P-value
Drug Perturbations from GEO up			
doxycycline DB00254 human GSE2624 sample 3076	38/272	3.93×10^{-36}	1.32×10^{-34}
doxycycline DB00254 human GSE2624 sample 3075	28/242	2.49×10^{-24}	2.02×10^{-23}
doxycycline DB00254 human GSE2624 sample 3077	23/209	1.30×10^{-19}	6.43×10^{-19}
doxycycline DB00254 mouse GSE29848 sample 3208	25/291	1.30×10^{-18}	5.84×10^{-18}
doxycycline DB00254 mouse GSE29848 sample 3209	24/267	2.35×10^{-18}	1.03×10^{-17}
doxycycline DB00254 human GSE2624 sample 3074	16/175	1.07×10^{-12}	2.89×10^{-12}
doxycycline DB00254 mouse GSE29848 sample 3207	17/225	4.54×10^{-12}	1.16×10^{-11}
ascorbic acid 54670067 human GSE11919 sample 3190	15/313	4.42×10^{-8}	8.64×10^{-8}
isotretinoin DB00982 human GSE10432 sample 2772	19/308	8.45×10^{-12}	2.10×10^{-11}
isotretinoin 5282379 human GSE10433 sample 2498	10/245	3.39×10^{-5}	5.51×10^{-5}
pioglitazone DB01132 rat GSE21329 sample 2843	40/400	3.44×10^{-32}	7.08×10^{-31}
pioglitazone DB01132 rat GSE21329 sample 2842	20/349	8.84×10^{-12}	2.18×10^{-11}
pioglitazone 4829 mouse GSE1458 sample 2587	19/318	1.47×10^{-11}	3.55×10^{-11}
pioglitazone DB01132 rat GSE20219 sample 2794	18/292	3.13×10^{-11}	7.40×10^{-11}
pioglitazone DB01132 human GSE8157 sample 2796	13/331	3.36×10^{-6}	5.89×10^{-6}
pioglitazone DB01132 rat GSE21329 sample 2841	11/279	1.88×10^{-5}	3.11×10^{-5}
pioglitazone DB01132 rat GSE20219 sample 2795	9/330	1.58×10^{-3}	2.31×10^{-3}
Drug Perturbations from GEO down			
doxycycline DB00254 human GSE2624 sample 3077	48/391	3.82×10^{-43}	3.45×10^{-41}
doxycycline DB00254 human GSE2624 sample 3074	39/425	6.14×10^{-30}	9.09×10^{-29}
doxycycline DB00254 human GSE2624 sample 3076	30/328	5.30×10^{-23}	4.02×10^{-22}
doxycycline DB00254 human GSE2624 sample 3075	27/358	1.40×10^{-18}	6.83×10^{-18}
doxycycline DB00254 mouse GSE29848 sample 3207	21/375	3.98×10^{-12}	1.21×10^{-11}
doxycycline DB00254 mouse GSE29848 sample 3208	16/309	5.14×10^{-9}	1.21×10^{-8}
doxycycline DB00254 mouse GSE29848 sample 3209	14/333	6.21×10^{-7}	1.28×10^{-6}
ascorbic acid 54670067 human GSE11919 sample 3190	40/287	5.09×10^{-38}	1.84×10^{-36}
isotretinoin DB00982 human GSE10432 sample 2772	7/292	1.02×10^{-2}	1.57×10^{-2}
pioglitazone DB01132 rat GSE21329 sample 2841	43/321	3.57×10^{-40}	1.90×10^{-38}
pioglitazone 4829 mouse GSE1458 sample 2587	24/282	8.34×10^{-18}	3.77×10^{-17}
pioglitazone DB01132 rat GSE21329 sample 2842	18/251	2.50×10^{-12}	7.64×10^{-12}
pioglitazone DB01132 rat GSE20219 sample 2794	17/308	6.28×10^{-10}	1.62×10^{-9}
pioglitazone DB01132 human GSE8157 sample 2796	14/269	4.58×10^{-8}	1.02×10^{-7}
pioglitazone DB01132 rat GSE20219 sample 2795	12/270	2.29×10^{-6}	4.52×10^{-6}
pioglitazone DB01132 rat GSE21329 sample 2843	7/200	1.29×10^{-3}	2.14×10^{-3}
tibolone 444008 human GSE12446 sample 3204	30/313	1.34×10^{-23}	1.14×10^{-22}
