# Supplementary material for: A new advanced in silico drug discovery method for novel coronavirus (SARS-CoV-2) with tensor decomposition-based unsupervised feature extraction
Source: PLoS One. 2020 Sep 11;15(9):e0238907. doi: 10.1371/journal.pone.0238907 (PMC7485840; doi:10.1371/journal.pone.0238907)
Supplement: S29 Table — List of in silico screened drugs [27] whose target genes are also among the 163 genes selected by TD based unsupervised FE. (PDF) [file pone.0238907.s029.pdf]

S29 Table: List of *in silico* screened drugs [25] whose target genes are also among the 163 genes selected by TD based unsupervised FE.

Term	Overlap	P-value	Adjusted P-value
Drug Perturbations from GEO up			
quercetin 5280343 human GSE7259 sample 3416	50/327	6.03×10^{-50}	1.36×10^{-47}
quercetin 5280343 human GSE7259 sample 3415	47/336	6.05×10^{-45}	6.85×10^{-43}
quercetin 5280343 rat GSE7479 sample 3409	38/394	5.73×10^{-30}	9.80×10^{-29}
quercetin 5280343 human GSE13899 sample 3182	19/307	7.99×10^{-12}	1.99×10^{-11}
quercetin DB04216 mouse GSE38136 sample 3436	17/297	3.59×10^{-10}	7.85×10^{-10}
quercetin DB04216 mouse GSE38141 sample 3435	16/280	1.25×10^{-9}	2.67×10^{-9}
quercetin DB04216 mouse GSE38136 sample 3438	15/254	2.69×10^{-9}	5.66×10^{-9}
quercetin DB04216 mouse GSE38067 sample 3440	13/227	4.62×10^{-8}	9.01×10^{-8}
quercetin DB04216 mouse GSE38136 sample 3437	16/472	1.66×10^{-6}	2.96×10^{-6}
quercetin DB04216 mouse GSE38067 sample 3441	7/114	4.16×10^{-5}	6.73×10^{-5}
quercetin DB04216 mouse GSE4262 sample 3428	11/360	1.85×10^{-4}	2.86×10^{-4}
quercetin DB04216 mouse GSE4262 sample 3429	8/229	5.94×10^{-4}	8.90×10^{-4}
quercetin DB04216 mouse GSE4262 sample 3427	9/360	2.84×10^{-3}	4.06×10^{-3}
quercetin DB04216 mouse GSE4262 sample 3433	8/323	5.09×10^{-3}	7.12×10^{-3}
quercetin DB04216 human GSE15162 sample 3444	7/323	1.69×10^{-2}	2.25×10^{-2}
quercetin DB04216 mouse GSE4262 sample 3434	7/324	1.71×10^{-2}	2.27×10^{-2}
Drug Perturbations from GEO down			
quercetin DB04216 mouse GSE38067 sample 3441	35/486	2.68×10^{-23}	2.11×10^{-22}
quercetin 5280343 human GSE13899 sample 3182	28/293	5.05×10^{-22}	3.40×10^{-21}
quercetin 5280343 rat GSE7479 sample 3409	16/206	1.31×10^{-11}	3.90×10^{-11}
quercetin DB04216 mouse GSE38141 sample 3435	17/320	1.13×10^{-9}	2.79×10^{-9}
quercetin DB04216 mouse GSE38136 sample 3436	16/303	3.89×10^{-9}	9.26×10^{-9}
quercetin DB04216 mouse GSE38067 sample 3440	15/373	4.27×10^{-7}	8.83×10^{-7}
quercetin 5280343 human GSE7259 sample 3415	12/264	1.81×10^{-6}	3.59×10^{-6}
quercetin DB04216 mouse GSE38136 sample 3438	13/346	5.44×10^{-6}	1.05×10^{-5}
quercetin DB04216 mouse GSE38136 sample 3437	8/128	1.02×10^{-5}	1.92×10^{-5}
quercetin DB04216 mouse GSE4262 sample 3430	11/312	5.22×10^{-5}	9.45×10^{-5}
quercetin DB04216 mouse GSE4262 sample 3431	10/348	5.87×10^{-4}	9.96×10^{-4}
quercetin 5280343 human GSE7259 sample 3416	8/273	1.83×10^{-3}	3.02×10^{-3}
quercetin DB04216 mouse GSE4262 sample 3428	7/240	3.59×10^{-3}	5.74×10^{-3}
quercetin DB04216 mouse GSE4262 sample 3429	7/371	3.27×10^{-2}	4.73×10^{-2}
