# Supplementary material for: A new advanced in silico drug discovery method for novel coronavirus (SARS-CoV-2) with tensor decomposition-based unsupervised feature extraction
Source: PLoS One. 2020 Sep 11;15(9):e0238907. doi: 10.1371/journal.pone.0238907 (PMC7485840; doi:10.1371/journal.pone.0238907)
Supplement: S30 Table — Five Drugs ranked within top 10 in the previous study but not in the present study in “DrugMatrix” category in Enrichr. They were still significantly enriched for the selected 163 genes. If there were more than ten hits, they were omitted. (PDF) [file pone.0238907.s030.pdf]

S30 Table: Five Drugs ranked within top 10 in the previous study but not in the present study in "DrugMatrix" category in Enrichr. They were still significantly enriched for the selected 163 genes. If there were more than ten hits, they were omitted.

Term	Overlap	P-value	Adjusted P-value
Primaquine-45 mg/kg in CMC-Rat-Liver-5d-up	18/315	1.09×10^{-10}	3.34×10^{-9}
Primaquine-45 mg/kg in CMC-Rat-Liver-1d-up	15/337	1.16×10^{-7}	7.37×10^{-7}
Primaquine-45 mg/kg in CMC-Rat-Liver-3d-up	14/316	3.31×10^{-7}	1.66×10^{-6}
Primaquine-45 mg/kg in CMC-Rat-Liver-3d-dn	9/284	5.51×10^{-4}	8.62×10^{-4}
Primaquine-45 mg/kg in CMC-Rat-Liver-5d-dn	7/285	8.98×10^{-3}	1.06×10^{-2}
Cytarabine-487 mg/kg in Saline-Rat-Bone marrow-1d-up	17/326	1.49×10^{-9}	2.47×10^{-8}
Cytarabine-23 mg/kg in Saline-Rat-Liver-0.25d-up	16/313	6.17×10^{-9}	7.17×10^{-8}
Cytarabine-487 mg/kg in Saline-Rat-Liver-1d-dn	14/237	9.28×10^{-9}	9.81×10^{-8}
Cytarabine-23 mg/kg in Saline-Rat-Bone marrow-0.25d-up	17/385	1.79×10^{-8}	1.66×10^{-7}
Cytarabine-23 mg/kg in Saline-Rat-Spleen-3d-up	15/299	2.42×10^{-8}	2.12×10^{-7}
Cytarabine-487 mg/kg in Saline-Rat-Liver-5d-dn	14/291	1.21×10^{-7}	7.59×10^{-7}
Cytarabine-23 mg/kg in Saline-Rat-Liver-5d-dn	14/307	2.33×10^{-7}	1.26×10^{-6}
Cytarabine-487 mg/kg in Saline-Rat-Kidney-5d-dn	14/319	3.71×10^{-7}	1.84×10^{-6}
Cytarabine-487 mg/kg in Saline-Rat-Kidney-3d-dn	14/327	4.99×10^{-7}	2.35×10^{-6}
Cytarabine-487 mg/kg in Saline-Rat-Spleen-1d-up	14/329	5.37×10^{-7}	2.49×10^{-6}
Cytarabine-23 mg/kg in Saline-Rat-Spleen-0.25d-up	14/344	9.14×10^{-7}	3.83×10^{-6}
(additional 31 hits with less significance are omitted)			
Pyrogallol-1000 mg/kg in Water-Rat-Liver-5d-up	14/304	2.07×10^{-7}	1.14×10^{-6}
Pyrogallol-1000 mg/kg in Water-Rat-Liver-1d-up	15/409	1.35×10^{-6}	5.23×10^{-6}
Pyrogallol-1000 mg/kg in Water-Rat-Liver-5d-dn	12/296	5.88×10^{-6}	1.76×10^{-5}
Pyrogallol-1000 mg/kg in Water-Rat-Liver-3d-up	13/349	5.97×10^{-6}	1.78×10^{-5}
Pyrogallol-1000 mg/kg in Water-Rat-Liver-3d-dn	7/251	4.59×10^{-3}	5.69×10^{-3}
Pyrogallol-1000 mg/kg in Water-Rat-Liver-1d-dn	5/191	2.03×10^{-2}	2.26×10^{-2}
Catechol-195 mg/kg in Saline-Rat-Liver-0.25d-up	19/290	2.94×10^{-12}	2.41×10^{-10}
Catechol-40 mg/kg in Saline-Rat-Liver-0.25d-up	19/305	7.13×10^{-12}	4.16×10^{-10}
Catechol-195 mg/kg in Saline-Rat-Bone marrow-1d-dn	16/305	4.27×10^{-9}	5.49×10^{-8}
Catechol-40 mg/kg in Saline-Rat-Kidney-0.25d-dn	16/319	8.08×10^{-9}	8.82×10^{-8}
Catechol-40 mg/kg in Saline-Rat-Kidney-3d-dn	15/294	1.93×10^{-8}	1.77×10^{-7}
Catechol-40 mg/kg in Saline-Rat-Bone marrow-0.25d-dn	15/306	3.28×10^{-8}	2.67×10^{-7}
Catechol-40 mg/kg in Saline-Rat-Bone marrow-1d-dn	15/307	3.43×10^{-8}	2.76×10^{-7}
Catechol-195 mg/kg in Saline-Rat-Spleen-1d-up	15/320	5.91×10^{-8}	4.31×10^{-7}
Catechol-195 mg/kg in Saline-Rat-Kidney-5d-dn	14/281	7.87×10^{-8}	5.39×10^{-7}
Catechol-195 mg/kg in Saline-Rat-Bone marrow-5d-dn	14/310	2.62×10^{-7}	1.38×10^{-6}
(additional 27 hits with less significance are omitted)			
Neomycin-877 mg/kg in Corn Oil-Rat-Kidney-1d-dn	14/264	3.62×10^{-8}	2.88×10^{-7}
Neomycin-877 mg/kg in Corn Oil-Rat-Liver-5d-up	14/323	4.31×10^{-7}	2.08×10^{-6}
Neomycin-56 mg/kg in Corn Oil-Rat-Kidney-0.25d-dn	12/256	1.31×10^{-6}	5.12×10^{-6}
Neomycin-877 mg/kg in Corn Oil-Rat-Kidney-3d-up	13/311	1.69×10^{-6}	6.23×10^{-6}
Neomycin-56 mg/kg in Corn Oil-Rat-Kidney-5d-dn	12/270	2.29×10^{-6}	7.99×10^{-6}
Neomycin-56 mg/kg in Corn Oil-Rat-Liver-5d-up	12/279	3.21×10^{-6}	1.06×10^{-5}
Neomycin-56 mg/kg in Corn Oil-Rat-Kidney-3d-dn	11/233	3.43×10^{-6}	1.12×10^{-5}
Neomycin-877 mg/kg in Corn Oil-Rat-Kidney-3d-dn	12/289	4.60×10^{-6}	1.44×10^{-5}
Neomycin-877 mg/kg in Corn Oil-Rat-Liver-0.25d-dn	12/296	5.88×10^{-6}	1.76×10^{-5}
Neomycin-56 mg/kg in Corn Oil-Rat-Liver-3d-dn	12/309	9.07×10^{-6}	2.50×10^{-5}
(additional 20 hits with less significance are omitted)			
