# Supplementary material for: A new advanced in silico drug discovery method for novel coronavirus (SARS-CoV-2) with tensor decomposition-based unsupervised feature extraction
Source: PLoS One. 2020 Sep 11;15(9):e0238907. doi: 10.1371/journal.pone.0238907 (PMC7485840; doi:10.1371/journal.pone.0238907)
Supplement: S31 Table — Four Drugs ranked within top 10 in the previous study but not in the present study in “Drug Pert from GEO up/down” category in Enrichr. They were still significantly enriched toward the selected 163 genes. (PDF) [file pone.0238907.s031.pdf]

S31 Table: Four Drugs ranked within top 10 in the previous study but not in the present study in "Drug Pert from GEO up/down" category in Enrichr. They were still significantly enriched toward the selected 163 genes.

Term	Overlap	P-value	Adjusted P-value
Drug Perturbations from GEO up			
fenretinide 5288209 rat GSE3952 sample 3561	35/397	2.98×10^{-26}	2.81×10^{-25}
fenretinide 5288209 rat GSE3952 sample 3559	7/160	3.45×10^{-4}	5.30×10^{-4}
pioglitazone DB01132 rat GSE21329 sample 2843	40/400	3.44×10^{-32}	7.08×10^{-31}
pioglitazone DB01132 rat GSE21329 sample 2842	20/349	8.84×10^{-12}	2.18×10^{-11}
pioglitazone 4829 mouse GSE1458 sample 2587	19/318	1.47×10^{-11}	3.55×10^{-11}
pioglitazone DB01132 rat GSE20219 sample 2794	18/292	3.13×10^{-11}	7.40×10^{-11}
pioglitazone DB01132 human GSE8157 sample 2796	13/331	3.36×10^{-6}	5.89×10^{-6}
pioglitazone DB01132 rat GSE21329 sample 2841	11/279	1.88×10^{-5}	3.11×10^{-5}
pioglitazone DB01132 rat GSE20219 sample 2795	9/330	1.58×10^{-3}	2.31×10^{-3}
decitabine DB01262 human GSE29077 sample 2546	31/243	3.22×10^{-28}	3.99×10^{-27}
decitabine DB01262 human GSE29077 sample 2538	31/263	3.84×10^{-27}	4.05×10^{-26}
decitabine DB01262 human GSE9118 sample 2703	31/271	9.77×10^{-27}	9.73×10^{-26}
decitabine DB01262 human GSE29077 sample 2539	26/279	3.22×10^{-20}	1.69×10^{-19}
decitabine 451668 mouse GSE4768 sample 3103	25/251	3.55×10^{-20}	1.85×10^{-19}
decitabine 451668 mouse GSE4768 sample 3105	26/287	6.59×10^{-20}	3.36×10^{-19}
decitabine DB01262 human GSE29077 sample 2540	19/300	5.34×10^{-12}	1.35×10^{-11}
decitabine DB01262 human GSE29077 sample 2547	19/304	6.73×10^{-12}	1.69×10^{-11}
decitabine DB01262 human GSE29077 sample 2548	19/316	1.32×10^{-11}	3.21×10^{-11}
decitabine 451668 mouse GSE4768 sample 3108	12/374	5.91×10^{-5}	9.43×10^{-5}
troglitazone DB00197 rat GSE21329 sample 2833	36/408	5.13×10^{-27}	5.34×10^{-26}
troglitazone DB00197 rat GSE21329 sample 2834	28/198	8.28×10^{-27}	8.42×10^{-26}
troglitazone 5591 mouse GSE1458 sample 2589	26/305	3.05×10^{-19}	1.45×10^{-18}
troglitazone DB00197 rat GSE21329 sample 2832	10/245	3.39×10^{-5}	5.52×10^{-5}
Drug Perturbations from GEO down			
fenretinide 5288209 rat GSE3952 sample 3559	38/440	3.49×10^{-28}	4.56×10^{-27}
fenretinide 5288209 rat GSE3952 sample 3561	22/203	1.18×10^{-18}	5.84×10^{-18}
pioglitazone DB01132 rat GSE21329 sample 2841	43/321	3.57×10^{-40}	1.90×10^{-38}
pioglitazone 4829 mouse GSE1458 sample 2587	24/282	8.34×10^{-18}	3.77×10^{-17}
pioglitazone DB01132 rat GSE21329 sample 2842	18/251	2.50×10^{-12}	7.64×10^{-12}
pioglitazone DB01132 rat GSE20219 sample 2794	17/308	6.28×10^{-10}	1.62×10^{-9}
pioglitazone DB01132 human GSE8157 sample 2796	14/269	4.58×10^{-8}	1.02×10^{-7}
pioglitazone DB01132 rat GSE20219 sample 2795	12/270	2.29×10^{-6}	4.52×10^{-6}
pioglitazone DB01132 rat GSE21329 sample 2843	7/200	1.29×10^{-3}	2.14×10^{-3}
decitabine DB01262 human GSE29077 sample 2540	44/300	6.35×10^{-43}	5.21×10^{-41}
decitabine DB01262 human GSE29077 sample 2539	41/321	2.15×10^{-37}	7.19×10^{-36}
decitabine 451668 mouse GSE4768 sample 3108	35/226	6.98×10^{-35}	1.91×10^{-33}
decitabine DB01262 human GSE29077 sample 2538	37/337	3.06×10^{-31}	5.22×10^{-30}
decitabine DB01262 human GSE9118 sample 2703	29/329	8.47×10^{-22}	5.54×10^{-21}
decitabine DB01262 human GSE29077 sample 2548	25/284	7.22×10^{-19}	3.62×10^{-18}
decitabine DB01262 human GSE29077 sample 2547	21/296	4.08×10^{-14}	1.42×10^{-13}
decitabine 451668 mouse GSE4768 sample 3105	20/313	1.20×10^{-12}	3.76×10^{-12}
decitabine 451668 mouse GSE4768 sample 3103	16/349	2.85×10^{-8}	6.43×10^{-8}
decitabine DB01262 human GSE29077 sample 2546	14/357	1.42×10^{-6}	2.85×10^{-6}
troglitazone DB00197 rat GSE21329 sample 2832	37/355	2.09×10^{-30}	3.31×10^{-29}
troglitazone 5591 mouse GSE1458 sample 2589	17/295	3.24×10^{-10}	8.55×10^{-10}
troglitazone DB00197 rat GSE21329 sample 2834	16/402	1.98×10^{-7}	4.20×10^{-7}
troglitazone DB00197 rat GSE21329 sample 2833	11/192	5.16×10^{-7}	1.07×10^{-6}
