# Supplementary material for: A new advanced in silico drug discovery method for novel coronavirus (SARS-CoV-2) with tensor decomposition-based unsupervised feature extraction
Source: PLoS One. 2020 Sep 11;15(9):e0238907. doi: 10.1371/journal.pone.0238907 (PMC7485840; doi:10.1371/journal.pone.0238907)
Supplement: S32 Table — The number of human proteins reported to interact with listed SARS-CoV-2 proteins [36]. (PDF) [file pone.0238907.s032.pdf]

S32 Table: The number of human proteins reported to interact with listed SARS-CoV-2 proteins [34]

SARS-CoV2 E	SARS-CoV2 M	SARS-CoV2 N
859	1245	584
SARS-CoV2 nsp1	SARS-CoV2 nsp10	SARS-CoV2 nsp11
592	443	925
SARS-CoV2 nsp12	SARS-CoV2 nsp13	SARS-CoV2 nsp14
620	994	475
SARS-CoV2 nsp15	SARS-CoV2 nsp2	SARS-CoV2 nsp4
559	918	952
SARS-CoV2 nsp5	SARS-CoV2 nsp5_C145A	SARS-CoV2 nsp6
540	365	1019
SARS-CoV2 nsp7	SARS-CoV2 nsp8	SARS-CoV2 nsp9
893	1017	684
SARS-CoV2 orf10	SARS-CoV2 orf3a	SARS-CoV2 orf3b
872	974	753
SARS-CoV2 orf6	SARS-CoV2 orf7a	SARS-CoV2 orf8
779	971	1106
SARS-CoV2 orf9b	SARS-CoV2 orf9c	SARS-CoV2 Spike
732	1462	820
