# Supplementary material for: A new advanced in silico drug discovery method for novel coronavirus (SARS-CoV-2) with tensor decomposition-based unsupervised feature extraction
Source: PLoS One. 2020 Sep 11;15(9):e0238907. doi: 10.1371/journal.pone.0238907 (PMC7485840; doi:10.1371/journal.pone.0238907)
Supplement: S33 Table — Coincidence between 163 genes and human proteins whose numbers are reported in S32 Table. (PDF) [file pone.0238907.s033.pdf]

S33 Table: Coincidence between 163 genes and human proteins whose numbers are reported in S32 Table

SARS-CoV-2 proteins	P values	Odds Ratio
SARS-CoV2 E	6.55×10^{-27}	10.16
SARS-CoV2 M	1.38×10^{-26}	8.42
SARS-CoV2 N	4.61×10^{-24}	11.43
SARS-CoV2 nsp1	1.06×10^{-20}	10.00
SARS-CoV2 nsp10	3.40×10^{-20}	11.52
SARS-CoV2 nsp11	1.13×10^{-29}	10.66
SARS-CoV2 nsp12	4.87×10^{-20}	9.48
SARS-CoV2 nsp13	6.04×10^{-33}	11.17
SARS-CoV2 nsp14	1.75×10^{-22}	12.05
SARS-CoV2 nsp15	1.85×10^{-20}	10.23
SARS-CoV2 nsp2	4.81×10^{-33}	11.79
SARS-CoV2 nsp4	5.79×10^{-29}	10.26
SARS-CoV2 nsp5	3.78×10^{-25}	12.36
SARS-CoV2 nsp5_C145A	3.75×10^{-17}	11.39
SARS-CoV2 nsp6	9.47×10^{-26}	9.00
SARS-CoV2 nsp7	1.93×10^{-29}	10.81
SARS-CoV2 nsp8	1.11×10^{-29}	10.14
SARS-CoV2 nsp9	5.54×10^{-29}	12.24
SARS-CoV2 orf10	5.29×10^{-34}	12.37
SARS-CoV2 orf3a	2.06×10^{-28}	9.95
SARS-CoV2 orf3b	1.89×10^{-29}	11.80
SARS-CoV2 orf6	8.81×10^{-26}	10.37
SARS-CoV2 orf7a	1.69×10^{-28}	10.00
SARS-CoV2 orf8	5.94×10^{-28}	9.25
SARS-CoV2 orf9b	6.54×10^{-30}	12.12
SARS-CoV2 orf9c	1.11×10^{-28}	8.35
SARS-CoV2 Spike	8.22×10^{-26}	10.08
