# Supplementary material for: A new advanced in silico drug discovery method for novel coronavirus (SARS-CoV-2) with tensor decomposition-based unsupervised feature extraction
Source: PLoS One. 2020 Sep 11;15(9):e0238907. doi: 10.1371/journal.pone.0238907 (PMC7485840; doi:10.1371/journal.pone.0238907)
Supplement: S34 Table — Number of experiments associated with adjusted P-values in various Enrichr categories for the drugs identified in another study [37]. (PDF) [file pone.0238907.s034.pdf]

S34 Table: Number of experiments associated with adjusted P-values in various Enrichr categories for the drugs identified in another study[35]

	DrugMatrix	GEO down	GEO up	LINCS down	LINCS up
Mestranol	6				
Methotrexate	40	2			
Fluorouracil	4	4	6	1	
Hexestrol	4				
Mercaptopurine	17				
Paroxetine	10			1	
Vinblastine	17			1	3
Phenylbutazone	6				
Naloxone	5				
Hydralazine	14				
Vinorelbine	13				1
Carvedilol	20				1
Colchicine	13		1		
Amitriptyline	13			1	
Epinephrine	17				
Dactinomycin	6	2	1	5	1
Hydrocortisone	21				
Melatonin	7				
Methyltestosterone	6				
Omeprazole	20				
Testosterone	12		1		
Oxymetholone	5				
Progesterone	20	2	2		
Permethrin		1			
Mesalazine		1			
Menadione		1	1		3
Stanolone		1	2		
Methotrexate			1		
Sirolimus				47	50
Cladribine				2	1
Regorafenib				9	14
Temsirolimus				1	
Quinacrine				1	3
